# Impact of Perioperative Immunonutrition on Postoperative Outcomes in Patients with Upper Gastrointestinal Cancer: A Systematic Review and Meta-Analysis of Randomized Controlled Trials

**DOI:** 10.3390/nu16050577

**Published:** 2024-02-20

**Authors:** Ryota Matsui, Masano Sagawa, Noriyuki Inaki, Tetsu Fukunaga, Souya Nunobe

**Affiliations:** 1 Department of Gastroenterological Surgery, The Cancer Institute Hospital of Japanese Foundation for Cancer Research, Tokyo 135-8550, Japan; supreme0818@gmail.com; 2 Department of Upper Gastrointestinal Surgery, Juntendo University Hospital, Tokyo 113-8431, Japan; t2fukunaga@juntendo.ac.jp; 3 Department of Gastrointestinal Surgery/Breast Surgery, Graduate School of Medical Science, Kanazawa University, Kanazawa 920-8530, Japan; 4 Department of Surgery, Tokyo Women’s Medical University Adachi Medical Center, Tokyo 123-8558, Japan; masanosagawa@yahoo.co.jp

**Keywords:** gastrointestinal cancer, immunonutrition, nutritional intervention, perioperative nutrition

## Abstract

There is no consensus on the efficacy of perioperative immunonutrition in patients with upper gastrointestinal (GI) cancer surgery. We clarified the impact of perioperative immunonutrition on postoperative outcomes in patients with upper GI cancers. We searched MEDLINE (PubMed), MEDLINE (OVID), EMBASE, Cochrane Central Register of Controlled Trials, Web of Science Core Selection, and Emcare from 1981–2022 using search terms related to immunonutrition and upper GI cancer. We included randomized controlled trials. Intervention was defined as immunonutritional therapy, including arginine, n-3 omega fatty acids, or glutamine during the perioperative period. The control was defined as standard nutritional therapy. The primary outcomes were infectious complications, defined as events with a Clavien–Dindo classification grade ≥ II that occurred within 30 days after surgery. After screening, 23 studies were included in the qualitative synthesis and in the quantitative synthesis. The meta-analysis showed that immunonutrition reduced infectious complications (relative risk ratio: 0.72; 95% confidence interval: 0.57–0.92; certainty of evidence: Moderate) compared with standard nutritional therapy. In conclusion, nutritional intervention with perioperative immunonutrition in patients with upper GI cancers significantly reduced infectious complications. The effect of immunonutrition for upper GI cancers in reducing the risk of infectious complications was about 30%.

## 1. Introduction

For cancer patients, surgical resection is the main treatment, and one of the risk factors for problems after surgery is preoperative malnutrition [1,2]. Patients with gastrointestinal cancers often have worsened nutritional status, which ranges from 20–70% [3,4]. The immune system and tissue repair are impacted by malnutrition [3]. Furthermore, the surgical invasion-induced catabolic processes result in the loss of vital nutrients, which might induce immune response dysregulation and increase the risk of infectious complications [1,3]. To decrease infection and total postoperative complications, nutritional interventions are necessary to reduce preoperative malnutrition.

There is no consensus on the efficacy of perioperative immunonutrition in patients with upper GI cancer after surgery. An immunonutrition therapy using either arginine, n-3 omega fatty acids, or glutamine has been developed and used clinically to reduce infectious complications and postoperative length of stay [5]. A postoperative inflammatory response is modulated by immunonutrition, which reduces immunosuppression caused by inflammatory cytokines [6]. While standard nutritional therapy is used for malnutrition, immunonutrition is used not only for malnutrition but also to reduce invasion or restore immunity. However, its efficacy and optimal timing are clinically unresolved, especially in upper GI cancer surgeries. Therefore, we planned a systematic review and meta-analysis of perioperative immunonutrition in patients with upper GI cancers.

This study aimed to clarify the effectiveness of perioperative immunonutrition in patients undergoing elective surgery for upper GI cancer. We also investigated whether the recommendations for patients who are malnourished differed from those who are not malnourished. We hypothesized that perioperative immunonutrition reduces infectious complications in patients with and without malnourishment.

## 2. Materials and Methods

We conducted a systematic review of the relevant literature in accordance with the Cochrane Handbook, Preferred Reporting Items for Systematic Reviews and Meta-Analysis 2020 guidelines (PRISMA-2020), and the Minds Manual for Guideline Development 2020 [7,8,9]. The protocol was published in PROSPERO (CRD42023471825).

### 2.1. Eligibility Criteria and Study Selection

We included randomized controlled trials (RCTs) of patients aged over 18 years who underwent elective upper GI cancer surgery with perioperative immunonutrition. Intervention was defined as perioperative immunonutritional therapy, including arginine, n-3 omega fatty acids, or glutamine was given preoperatively, postoperatively, or both. The control was defined as standard oral or intravenous nutritional therapy without immunonutrition. The intervention and control groups received the same amount of nutrition. We excluded studies in which more than 25% of patients had benign disease or cancer at other sites, review articles, case reports, crossover trials, and cluster-, quasi-, and non-randomized trials.

### 2.2. Search Strategy

Appendix A provides the search formulae. We searched MEDLINE (PubMed), MEDLINE (OVID), Embase (OVID), Cochrane Central Register of Controlled Trials (CENTRAL), Web of Science Core Selection, and Emcare (OVID). The period covered by the RCT was 2000–2022.

### 2.3. Study Selection and Data Collection

Two independent reviewers screened the titles and abstracts, assessing their eligibility based on the full texts. The same reviewers performed independent data extraction from the included studies using a standardized data collection form. Reviewer disagreements were resolved through discussion or with a mediating third reviewer. The original authors were contacted for missing data.

### 2.4. Risk-of-Bias Assessment

Two of the three researchers carried out risk-of-bias (ROB) assessments using the Cochrane Collaboration ROB tool, which has five domains: randomization, deviation from intervention, missing data, measurement of outcome, and selective reporting [7]. The ratings “high risk”, “some concerns”, and “low risk” were assigned to each domain and overall. The resolution of the disagreement was decided by a third person.

### 2.5. Outcomes

The primary outcomes were the total postoperative and infectious complications. The secondary outcomes were severe complications, anastomotic leakage, postoperative pneumonia, postoperative mortality, nutritional intervention adverse events, and postoperative hospitalization. Postoperative complications were defined as events with a Clavien–Dindo (CD) classification grade of ≥II that occurred within 30 days after surgery. Severe complications were defined as those with a CD grade of ≥III.

### 2.6. Synthesis of Results

We pooled the relative risk ratios (RRs) and 95% confidence intervals (CIs) for postoperative complications, postoperative mortality, and nutritional intervention adverse events, and the mean differences (MDs) and 95% CIs for postoperative hospitalization in patients with upper GI cancer. An intention-to-treat analysis was performed for dichotomous data where possible. We used Review Manager software 5.4.2 and performed meta-analyses with a random-effects model, assuming that the true effect would be low owing to many unmeasured or unknown factors and individual differences between studies in accordance with the Cochrane Handbook [7].

Statistical heterogeneity was evaluated by visually inspecting forest plots and calculating the I^2^ statistic (I^2^ values of 0–40% may not be important; 30–60% may represent moderate heterogeneity; 50–90% may represent substantial heterogeneity; 75–100%, considerable heterogeneity) [7]. When there was substantial heterogeneity (I^2^ > 50%), we assessed the reason.

To elucidate the influence of effect modifiers, subgroup analyses according to malnutrition status (with or without malnutrition), intervention timing (preoperative, postoperative, or perioperative), cancer site (esophageal or gastric), and difference in ingredient (arginine absent or arginine present) were performed when sufficient data were available. We also performed a sensitivity analysis for the frequency of malnourishment (>50% or >75%). In one of these analyses, studies using imputed statistics were excluded, while the other included only participants who completed the study with complete data [7]. Potential publication bias was assessed by visual inspection of the funnel plots for outcomes in more than 10 studies [7].

### 2.7. Certainty Assessment

Based on the Cochrane Handbook [7], we summarized the findings for total postoperative complications, infectious complications, severe complications, anastomotic leakage, postoperative pneumonia, postoperative mortality, nutritional intervention adverse events, and postoperative hospitalization. The summary included grading of certainty of evidence (COE) according to the Grading of Recommendations Assessment, Development, and Evaluation (GRADE) approach [10]. We started with “high” COE [10]. If there were any serious concerns in any domain, we lowered the grade from “high” COE. The effect estimates displayed in the Summary of Findings table were created using RRs and MD. To determine the inconsistency domain of the GRADE ratings, we examined the consistency of the RR and MD.

## 3. Results

### 3.1. Study Selection

**Figure 1:**
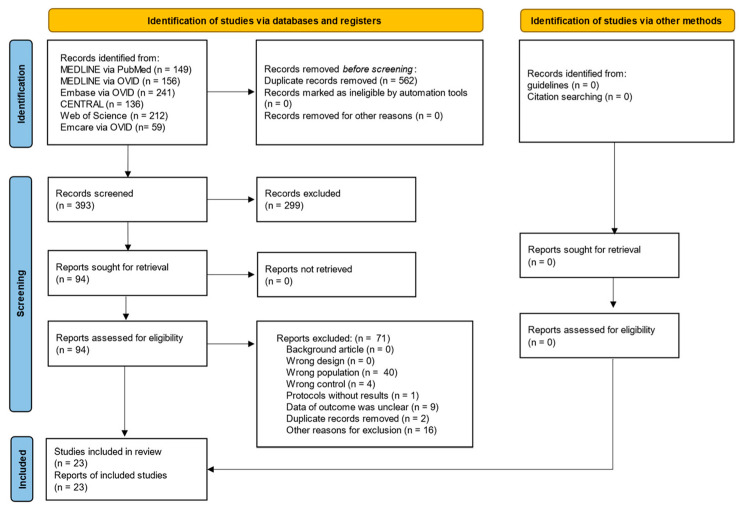
PRISMA 2020 flow diagram of this study.

The PRISMA flowchart is shown in Figure 1. A total of 391 records were searched on 16 October 2023. After screening, 23 studies (2249 patients) were included in the qualitative synthesis [11,12,13,14,15,16,17,18,19,20,21,22,23,24,25,26,27,28,29,30,31,32,33] and in the quantitative synthesis [11,12,13,14,15,16,17,18,19,20,21,22,23,24,25,26,27,28,29,30,31,32,33]. No unpublished data or ongoing studies were identified. The reasons for exclusion were incorrect population (*n* = 40), incorrect control (*n* = 4), protocol without results (*n* = 1), insufficient outcome data (*n* = 9), duplicate records (*n* = 2), and other reasons (*n* = 16).

### 3.2. Study and Patient Characteristics

Table 1 summarizes the characteristics of the 23 studies included in the quantitative synthesis. Of these studies, 10 were for esophageal cancer [11,12,13,16,17,21,29,31,32,33], 7 were for gastric cancer [14,15,18,19,20,23], and 6 were for mixed upper GI [22,24,25,27,28,30]. Regarding the nutritional intervention timing, 5 studies were conducted preoperatively [16,20,23,24,33], 8 were postoperatively [15,17,19,22,25,26,27,31], and 10 were preoperatively and postoperatively [11,12,13,14,18,21,28,29,31,32]. We did not find any literature that included only patients with malnutrition. Table 1 summarizes the findings using the GRADE approach.

### 3.3. Risk of Bias

**Figure 2:**
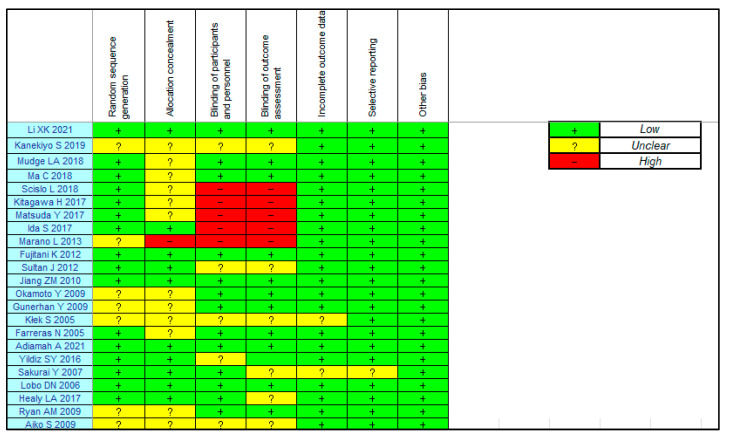
Risk of bias for the eligibility studies.

Figure 2 summarizes the ROB in the included studies. Regarding postoperative complications, there was a low ROB for incomplete outcome data and selective reporting and a low ROB or “some concerns” for random sequence generation and allocation concealment. The ROB for the participant and personnel blinding and outcome assessment were low”, some concerns” or high.

### 3.4. Meta-Analysis Results

**Figure 3:**
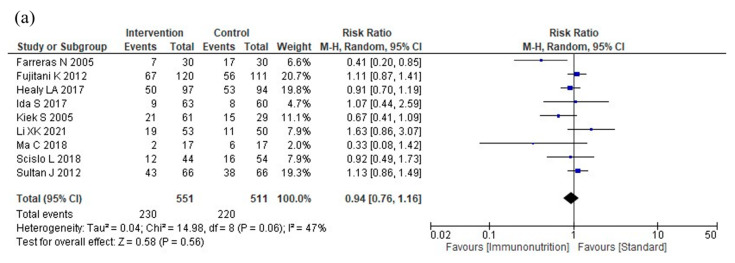

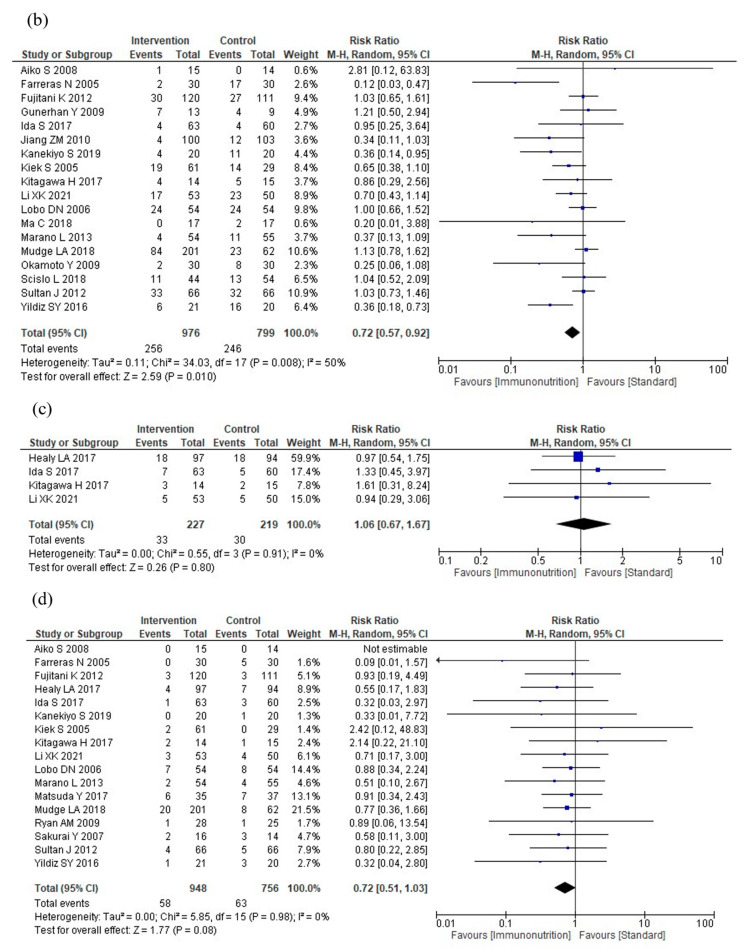

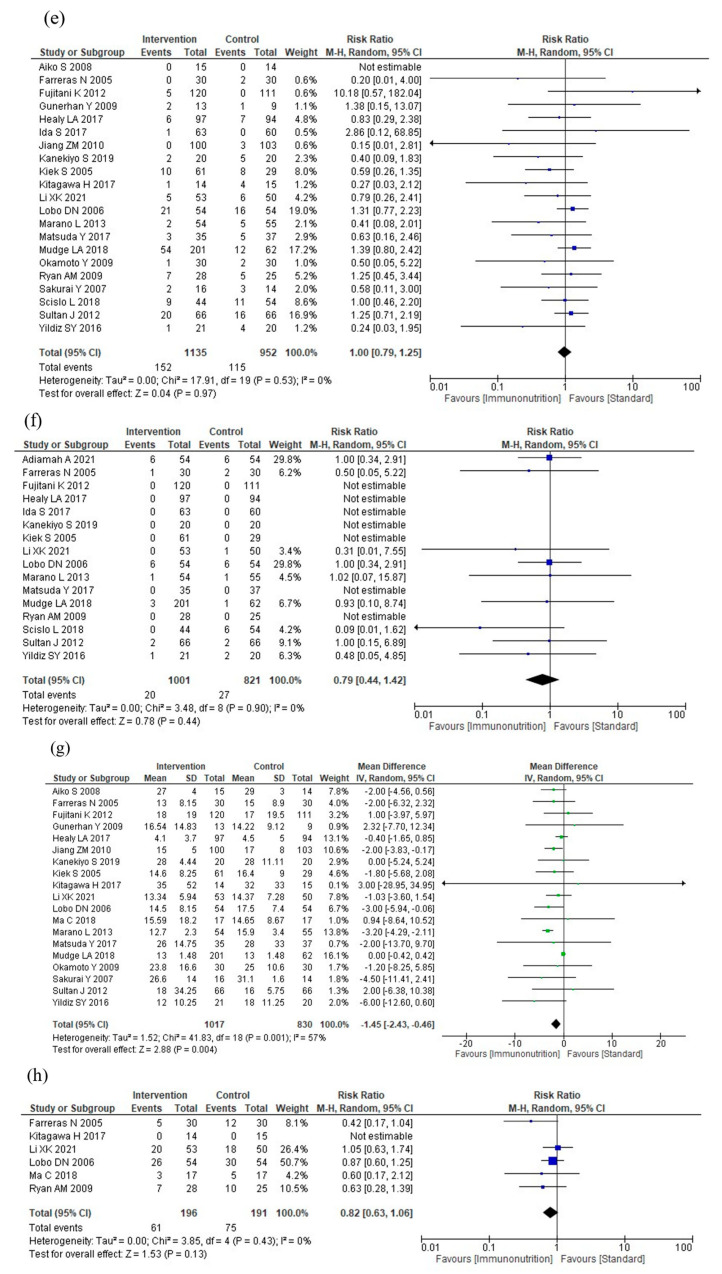
Results of meta-analysis: (**a**) total postoperative complications, (**b**) infectious complications, (**c**) severe complications, (**d**) anastomotic leakage, (**e**) postoperative pneumonia, (**f**) mortality, (**g**) postoperative hospital stay, and (**h**) nutritional intervention adverse events.

Table 2 shows a summary of the findings of this study. The results of the meta-analysis are shown in Figure 3.

Nine studies reported total postoperative complications. Immunonutrition does not reduce total postoperative complications compared to standard nutritional therapy (RR: 0.94, 95% CI: 0.76–1.16, I^2^ = 47%, *n* = 9, COE: moderate; Figure 3a).

Eighteen studies reported infectious complications. Immunonutrition reduces infectious complications compared to standard nutritional therapy (RR: 0.72, 95% CI: 0.57–0.92, I^2^ = 50%, *n* = 18, COE: moderate, Figure 3b).

Four studies reported severe complications. Immunonutrition is unlikely to reduce severe complications compared to standard nutritional therapy (RR: 1.06, 95% CI: 0.67–1.67, I^2^ = 0%, *n* = 4; COE: moderate; Figure 3c).

Seventeen studies reported anastomotic leakage. Immunonutrition probably reduces anastomotic leakage compared to standard nutritional therapy (RR: 0.72, 95% CI: 0.51–1.03, I^2^ = 0%, *n* = 17, COE: moderate, Figure 3d).

Twenty-one studies reported postoperative pneumonia. Immunonutrition is unlikely to reduce postoperative pneumonia compared to standard nutritional therapy (RR: 1.00, 95% CI: 0.79–1.25, I^2^ = 0%, *n* = 21, COE: moderate; Figure 3e).

Sixteen studies reported mortality rates. Immunonutrition is unlikely to reduce mortality compared to standard nutritional therapy (RR: 0.79, 95% CI: 0.44–1.42, I^2^ = 0%, *n* = 16, COE: moderate; Figure 3f).

Nineteen studies reported postoperative hospital stays. Immunonutrition reduces postoperative hospital stay compared to standard nutritional therapy (MD: −1.45, 95% CI: −2.43, −0.46, I^2^ = 57%, *n* = 19; COE: high, Figure 3g).

Six studies reported nutritional intervention adverse events. Immunonutrition is unlikely to increase nutritional intervention adverse events compared to standard nutritional therapy (RR: 0.82, 95% CI: 0.63–1.06, I^2^ = 0%, *n* = 6, COE: moderate, Figure 3h).

### 3.5. Subgroup Analyses

We performed subgroup analyses according to intervention timing (preoperative, postoperative, or perioperative), cancer site (esophageal or gastric), and difference in ingredient (arginine absent or arginine present). Subgroup and sensitivity analysis for malnutrition were not performed because of the paucity of studies that included patients with malnourishment.

#### 3.5.1. Subgroup Analyses According to Intervention Timing

**Figure 4:**
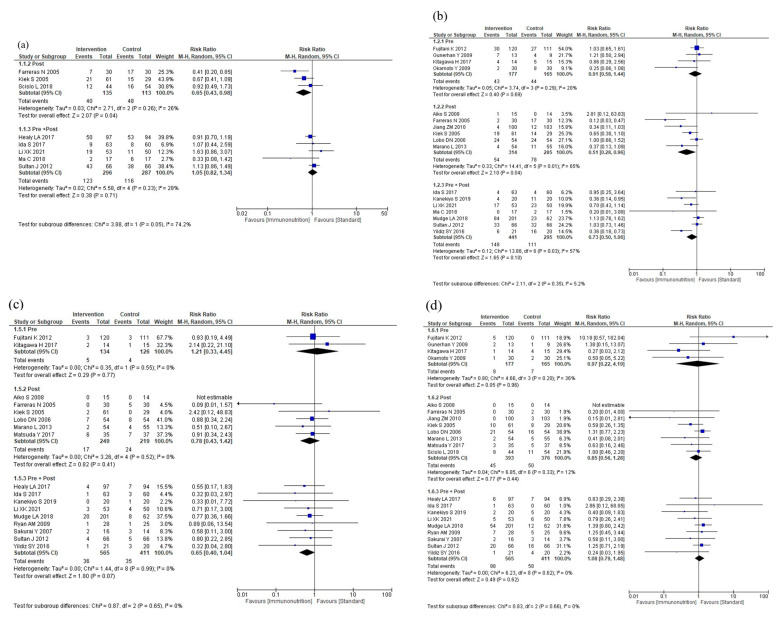

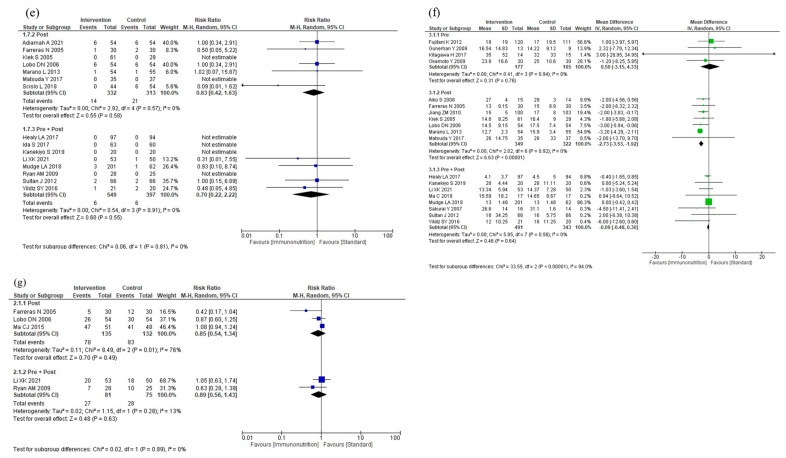
Results of subgroup analyses according to intervention timing: (**a**) total postoperative complications, (**b**) infectious complications, (**c**) anastomotic leakage, (**d**) postoperative pneumonia, (**e**) mortality, (**f**) postoperative hospital stay, and (**g**) nutritional intervention adverse event.

The results of the subgroup analyses according to intervention timing are shown in Figure 4. In the subgroup analysis, total postoperative complications were reduced for postoperative administration compared to preoperative plus postoperative administration (*p* = 0.05, Figure 4a). The subgroup analyses showed no efficacy differences between the groups according to the intervention timing for infectious complications (*p* = 0.42, Figure 4b), anastomotic leakage (*p* = 0.65, Figure 4c), postoperative pneumonia (*p* = 0.66, Figure 4d), and mortality (*p* = 0.81, Figure 4e). Postoperative hospital stay was shorter with postoperative administration compared to preoperative or preoperative plus postoperative administration (*p* < 0.001, Figure 4f). The subgroup analysis for adverse events showed no efficacy difference between the groups (*p* = 0.89, Figure 4g). The subgroup analysis for severe complications was not performed due to the small number of studies.

#### 3.5.2. Subgroup Analyses According to Cancer Site

**Figure 5:**
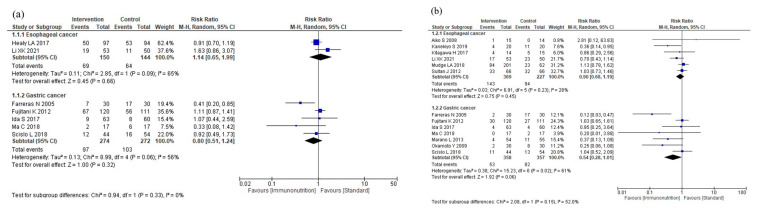

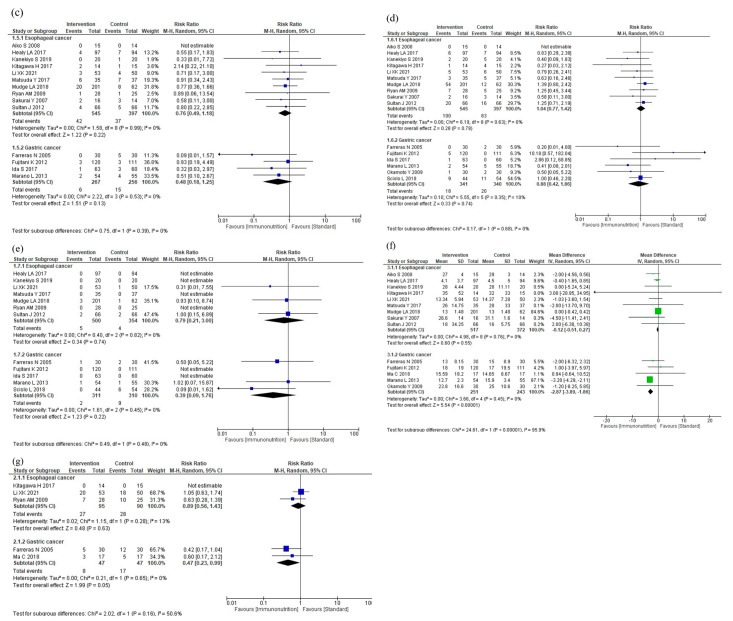
Results of subgroup analyses according to cancer site: (**a**) total postoperative complications, (**b**) infectious complications, (**c**) anastomotic leakage, (**d**) postoperative pneumonia, (**e**) mortality, (**f**) postoperative hospital stay, and (**g**) nutritional intervention adverse event.

The results of the subgroup analyses according to cancer site are shown in Figure 5. The subgroup analyses showed no efficacy differences between the groups according to the cancer site for total postoperative complications (*p* = 0.33, Figure 5a), infectious complications (*p* = 0.17, Figure 5b), anastomotic leakage (*p* = 0.39, Figure 5c), postoperative pneumonia (*p* = 0.68, Figure 5d), and mortality (*p* = 0.48, Figure 5e). Postoperative hospital stay was shorter in the gastric cancer group than in the esophageal cancer group (*p* < 0.001, Figure 5f). The subgroup analysis for adverse events showed no efficacy difference between the groups (*p* = 0.16, Figure 5g). The subgroup analysis for severe complications was not performed due to the small number of studies.

#### 3.5.3. Subgroup Analyses for Ingredient Difference

**Figure 6:**
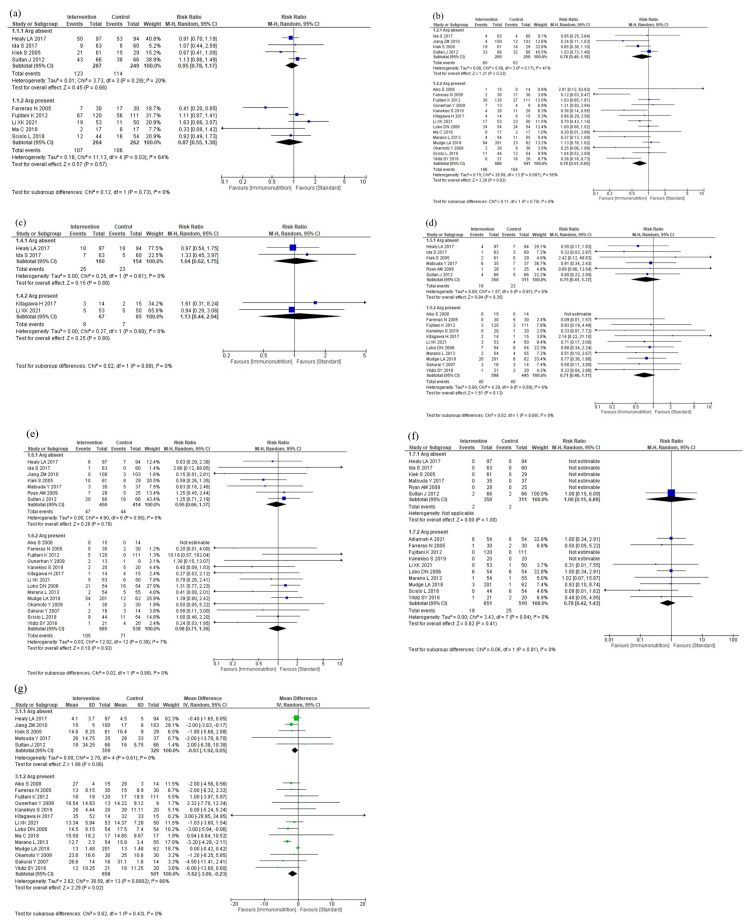
Results of subgroup analyses for ingredient difference: (**a**) total postoperative complications, (**b**) infectious complications, (**c**) severe complications, (**d**) anastomotic leakage, (**e**) postoperative pneumonia, (**f**) mortality, and (**g**) postoperative hospital stay.

The results of the subgroup analyses according to ingredient differences are shown in Figure 6. The subgroup analyses showed no efficacy differences between the groups according to the ingredient difference for total postoperative complications (*p* = 0.73, Figure 6a), infectious complications (*p* = 0.70, Figure 6b), severe complications (*p* = 0.88, Figure 6c), anastomotic leakage (*p* = 0.88, Figure 6d), postoperative pneumonia (*p* = 0.88, Figure 6e), mortality (*p* = 0.81, Figure 6f), and postoperative hospital stay (*p* = 0.49, Figure 6g). The subgroup analysis for adverse events was not performed due to the small number of studies.

#### 3.5.4. Subgroup Analyses for Total Duration

**Figure 7:**
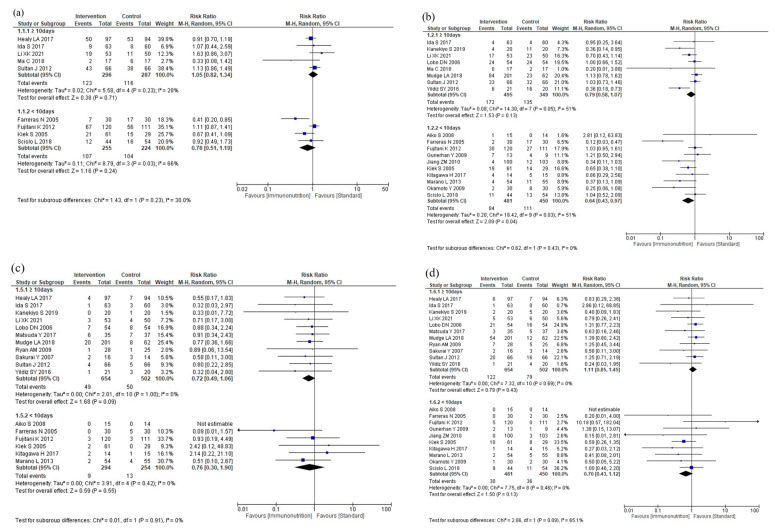

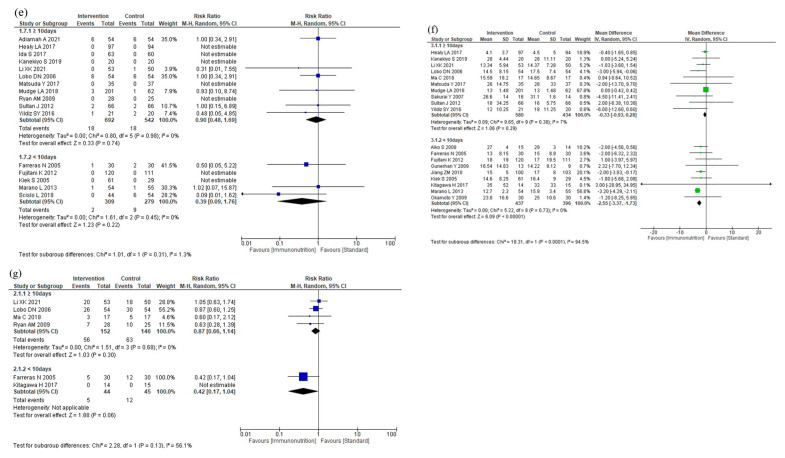
Results of subgroup analyses for total duration: (**a**) total postoperative complications, (**b**) infectious complications, (**c**) anastomotic leakage, (**d**) postoperative pneumonia, (**e**) mortality, (**f**) postoperative hospital stay, and (**g**) nutritional intervention adverse event.

The results of the subgroup analyses according to total duration are shown in Figure 7. The subgroup analyses showed no efficacy differences between the groups according to the total duration for total postoperative complications (*p* = 0.23, Figure 7a), infectious complications (*p* = 0.43, Figure 7b), anastomotic leakage (*p* = 0.91, Figure 7c), postoperative pneumonia (*p* = 0.09, Figure 7d), and mortality (*p* = 0.31, Figure 7e). Postoperative hospital stay was shorter in the <10 days group than in the ≥10 days group (*p* < 0.001, Figure 7f). The subgroup analysis for adverse events showed no efficacy difference between the groups (*p* = 0.13, Figure 7g). The subgroup analysis for severe complications was not performed due to the small number of studies.

#### 3.5.5. Subgroup Analyses for Malnutrition

**Figure 8:**
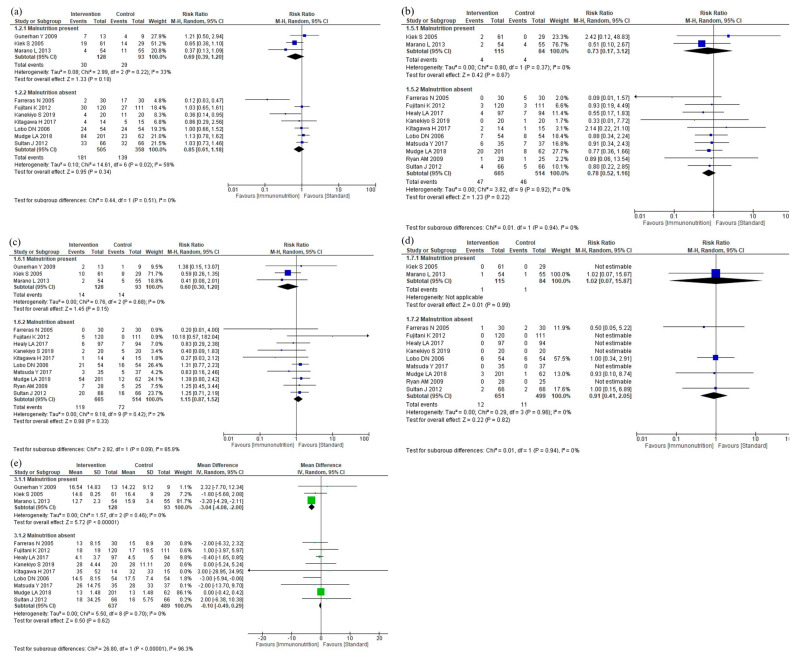
Results of subgroup analyses for malnutrition: (**a**) infectious complications, (**b**) anastomotic leakage, (**c**) postoperative pneumonia, (**d**) mortality, and (**e**) postoperative hospital stay.

The results of the subgroup analyses according to malnutrition are shown in Figure 8. The subgroup analyses showed no efficacy differences between the groups according to the malnutrition for infectious complications (*p* = 0.51, Figure 8a), anastomotic leakage (*p* = 0.94, Figure 8b), postoperative pneumonia (*p* = 0.09, Figure 8c), and mortality (*p* = 0.94, Figure 8d). Postoperative hospital stay was shorter in the malnutrition group than in the no-malnutrition group (*p* < 0.001, Figure 8e). The subgroup analyses for total complications, severe complications, and adverse events were not performed due to the small number of studies.

### 3.6. Funnel Plots

**Figure 9:**
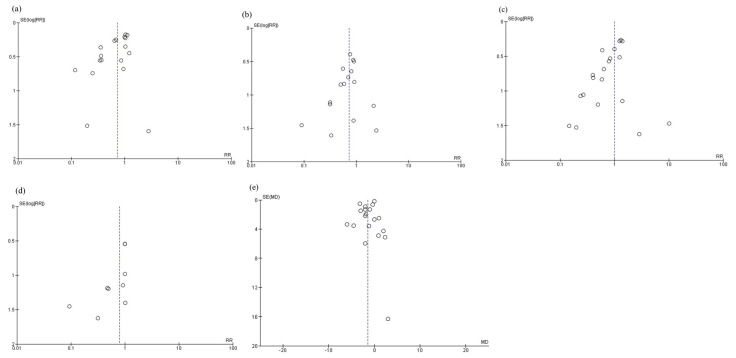
Funnel plots: (**a**) infectious complications, (**b**) anastomotic leakage, (**c**) postoperative pneumonia, (**d**) mortality, and (**e**) postoperative hospital stay.

The funnel plots are shown in Figure 9. Funnel plots were visualized as symmetrical, indicating minimal publication bias in the reporting of infectious complications (Figure 9a), anastomotic leakage (Figure 9b), postoperative pneumonia (Figure 9c), mortality (Figure 9d), and postoperative hospital stay (Figure 9e). For total postoperative complications, severe complications, and adverse events, funnel plots were not prepared because there were fewer than 10 references, as per the Cochrane Handbook [7].

## 4. Discussion

The results of the present systematic review and meta-analysis of 23 studies and 2249 patients revealed that immunonutrition probably reduces infectious complications in patients with upper GI cancers after surgery. In addition, immunonutrition probably reduces the rates of anastomotic leakage and the postoperative hospital stay. However, immunonutrition is unlikely to reduce the rate of total complications, severe complications, postoperative pneumonia, and postoperative mortality. Compared to standard nutrition, immunonutrition is unlikely to increase nutritional intervention adverse events. In a subgroup analysis, postoperative administration decreased the total number of postoperative complications and shortened the length of hospital stay. The subgroup analyses showed no efficacy differences between the groups according to the ingredient difference. We did not perform sensitivity analysis because of the paucity of studies that included patients with malnutrition. This study shows that compared to standard nutritional therapy, nutritional intervention with immunonutrition can reduce postoperative complications, especially infectious complications, without increasing nutrition-related adverse events.

There was no consensus on the impact of immunonutrition on postoperative outcomes in patients with upper GI cancers because different results have been reported; however, this may be due to the different numbers of studies included in the meta-analysis. A summary of the results of previous meta-analyses is presented in Table 3. Mingliang et al. [34] and Zhuo et al. [35] reported that immunonutrition does not reduce infectious complications for esophageal cancer. On the other hand, Tian et al. [36] reported a decrease in infectious complications (odds ratio: 0.48, 95% CI: 0.20–0.98). The difference between them is the number of RCTs included in the meta-analysis; the former may have been underpowered. Also, Song et al. [37] reported a reduction in infectious complications in patients with gastric cancer (OR: 0.56, 95% CI: 0.36–0.86). This study has the strength of summarizing a much larger number of studies compared to previous reports and showing a possible reduction in infectious complications.

Subgroup analysis by the timing of immunonutrition administration showed the differences in total postoperative complications and postoperative hospital stay for postoperative administration. Tian et al. [36] reported that postoperative administration in patients with esophageal cancer is associated with lower rates of infectious complications and postoperative pneumonia. Osland et al. [40] reported that perioperative and postoperative dosing in patients with GI cancer can reduce postoperative complications. Similarly, this present SR showed that postoperative administration may have been effective in patients with upper GI cancers.

The mechanisms by which immunonutrition improves postoperative outcomes include improved nutritional status for malnutrition and resistance to infection by modulating immune function. High levels of postoperative inflammation from invasive surgery cause immunodeficiency, although immunonutrition has the opposite consequence of excessive inflammation suppression [6]. According to one study, preoperative immunonutrition decreased inflammatory cytokines following pancreaticoduodenectomy, which may be responsible for the decrease in postoperative complications [41]. Ates et al. [42] reported that postoperative administration of immunonutrition resulted in lower postoperative CRP. These results suggest that pre- and postoperative administration may reduce inflammation. Additionally, it functions as an immunostimulation against inflammation following surgical invasion, which decreases immunity to infection and induces immunosuppression [3]. The former should be used preoperatively as modulating and the latter postoperatively as stimulating. In this study, subgroup analysis showed that immunonutrition with arginine was not effective in preventing postoperative complications and reducing hospital stays. This may mean that immune-enhancing nutrients are not effective in patients with upper GI cancer. These may have different results depending on the degree of surgical invasiveness and the extent of resection.

We performed subgroup analysis with a cutoff value of >50% malnutrition, but there was no difference in postoperative complications between patients with and without malnutrition, and only postoperative hospital stay was significantly different. There were no RCTs in which all patients were malnourished. Riso et al. [43] compared postoperative outcomes in patients with malnutrition in head and neck cancer and reported no severe complications in the immunonutrition group. In this subgroup analysis, the reason for the absence of significant differences in postoperative complications was due to the small number of RCTs that included malnutrition. Immunomodulation by administration of immunonutrition may be necessary in patients with malnutrition because of the possibility of immune impairment, but this was not evident in this study. Further studies on patients with malnutrition are required.

This study had several limitations. First, there are few RCTs in patients with malnutrition. Further studies are required in patients with malnutrition. Second, the mechanism by which immunonutrition may improve postoperative outcomes in upper GI cancers remains unclear. One hypothesis is that it suppresses postoperative inflammation. However, further studies are required. Third, the optimal immunonutritional dose remains unknown. Further RCTs with different dosage designs are required. Despite these limitations, the meta-analysis includes large numbers of RCTs, which addresses the problem of previous meta-analyses that were unable to show statistical differences despite clinical differences owing to the small number of RCTs. Our findings have several significant clinical implications.

## 5. Conclusions

Nutritional intervention with perioperative immunonutrition in patients with upper GI cancer significantly reduced infectious complications without increasing nutritional intervention adverse events. The effect of immunonutrition for upper GI cancers in reducing the risk of infectious complications was about 30%. Further studies with different dosage designs are required in patients with malnutrition.

## Figures and Tables

**Table 1:** Summary of included studies.

Author (Year)	Country	Cancer Type	Sample Size	Age (Mean)	BMI (Mean)	Male (%)	Malnutrition (%)	Intervention Timing
Li XK (2021) [11]	China	Esophageal	103	62.1	23.6	68.9	NA	Pre + Post
Kanekiyo S (2019) [12]	Japan	Esophageal	40	65.0	21.9	80.0	25.0	Pre + Post
Mudge LA (2018) [13]	Denmark	Esophageal	276	62.5	26.9	80.8	16.0	Pre + Post
Ma C (2018) [14]	Taiwan	Gastric	34	60.2	24.2	64.7	NA	Pre + Post
Scislo L (2018) [15]	Poland	Gastric	98	62.9	24.7	72.5	NA	Post
Kitagawa H (2017) [16]	Japan	Esophageal	29	67.1	21.0	75.9	24.1	Pre
Matsuda Y (2017) [17]	Japan	Esophageal	72	64.1	21.3	75.0	34.7	Post
Ida S (2017) [18]	Japan	Gastric	123	65.0	22.7	72.4	NA	Pre + Post
Marano L (2013) [19]	Italy	Gastric	109	66.6	23.1	65.1	57.8	Post
Fujitani K (2012) [20]	Japan	Gastric	231	64.0	22.7	72.4	2.2	Pre
Sultan J (2012) [21]	UK	Esophageal	132	67.0	25.8	75.8	8.3	Pre + Post
Jiang ZM (2010) [22]	China	Upper GI	203	58.2	23.1	64.5	NA	Post
Okamoto Y (2009) [23]	Japan	Gastric	60	66.9	NA	70.0	NA	Pre
Gunerhan Y (2009) [24]	Turkey	Upper GI	29	64.6	24.1	41.4	55.2	Pre
Kłek S (2005) [25]	Poland	Upper GI	90	61.9	NA	56.7	54.4	Post
Farreras N (2005) [26]	Spain	Gastric	60	68.0	NA	53.3	21.7	Post
Adiamah A (2021) [27]	UK	Upper GI	108	66.6	NA	76.9	NA	Post
Yildiz SY (2016) [28]	Turkey	Upper GI	41	64.1	22.0	70.7	NA	Pre + Post
Sakurai Y (2007) [29]	Japan	Esophageal	30	63.0	NA	43.3	NA	Pre + Post
Lobo DN (2006) [30]	UK	Upper GI	108	66.2	NA	76.9	8.3	Post
Healy LA (2017) [31]	Ireland	Esophageal	191	62.0	28.1	79.1	4.2	Pre + Post
Ryan AM (2009) [32]	Ireland	Esophageal	53	63.9	25.9	71.7	4.0	Pre + Post
Aiko S (2009) [33]	Japan	Esophageal	29	62.1	NA	90.0	NA	Post

BMI—body mass index, GI—gastrointestinal, NA—not applicable, UK—United Kingdom.

**Table 2:** Summary of findings.

Outcomes	Relative Effect (95% CI)	Sample Size (Studies)	Certainty of the Evidence (GRADE)	Comments
Total postoperative complications	RR 0.94 (0.76 to 1.16)	1062 (9 RCTs)	⊕⊕⊕⊝ Moderate ^a^	Immunonutrition probably does not reduce total postoperative complications.
Infectious complications	RR 0.72 (0.57 to 0.92)	1775 (18 RCTs)	⊕⊕⊕⊝ Moderate ^b^	Immunonutrition probably reduces infectious complications.
Severe complications	RR 1.06 (0.67 to 1.67)	446 (4 RCTs)	⊕⊕⊕⊝ Moderate ^a^	Immunonutrition probably does not reduce severe complications.
Anastomotic leakage	RR 0.72 (0.51 to 1.03)	1704 (17 RCTs)	⊕⊕⊕⊝ Moderate ^a^	Immunonutrition probably reduces anastomotic leakage.
Postoperative pneumonia	RR 1.00 (0.79 to 1.25)	2087 (21 RCTs)	⊕⊕⊕⊝ Moderate ^a^	Immunonutrition probably does not reduce postoperative pneumonia.
Mortality	RR 0.79 (0.44 to 1.42)	1822 (16 RCTs)	⊕⊕⊕⊝ Moderate ^a^	Immunonutrition probably does not reduce mortality.
Postoperative hospital stay	MD −1.45 (−2.43 to −0.46)	1847 (19 RCTs)	⊕⊕⊕⊕ High	Immunonutrition reduces postoperative hospital stays.
Adverse events	RR 0.82 (0.63 to 1.06)	387 (6 RCTs)	⊕⊕⊕⊝ Moderate ^a^	Immunonutrition probably does not increase adverse events.

CI—confidence interval; MD—mean difference; RCT—randomized control trials; RR—risk ratio. ^a^ Downgraded one point because of the inconsistency of the forest plot. ^b^ Downgraded one point because of inconsistency due to substantial heterogeneity.

**Table 3:** Summary of meta-analyses.

Author (Year)	Type of Surgery	Number of Studies	Number of Patients	Intervention	Control	Outcome and Effect
Wong, C.S. (2016) [38]	Upper GI Surgery	19 RCTs	2016	Enteral IMN	Standard EN	Wound infection (RR 0.69, 95%CI: 0.50, 0.94) Length of hospital stay (MD: −2.51, 95%CI: −3.47, −1.55) Anastomotic leakage (RR: 0.68, 95%CI: 0.42, 1.10) Pneumonia (RR: 0.81, 95%CI: 0.65, 1.01) Mortality (RR: 0.77, 95%CI: 0.43, 1.37)
Mingliang, W. (2020) [34]	Esophagectomy	7 RCTs	606	Enteral IMN	Standard EN	Infectious complications (RR: 0.97, 95%CI: 0.78, 1.20) Pneumonia (RR: 0.97, 95%CI: 0.71, 1.33) Wound infection (RR 0.80, 95%CI: 0.46, 1.40) Anastomotic leakage (RR: 0.59, 95%CI: 0.33, 1.04)
Fu, H. (2022) [39]	Total gastrectomy	10 RCTs	1056	Enteral IMN	Standard EN	Wound infection (OR 0.77, 95%CI: 0.50, 1.19) Infectious complications (OR: 0.72, 95%CI: 0.48, 1.09)
Zhuo, Z.G. (2021) [35]	Esophagectomy	6 RCTs	638	Enteral IMN	Standard EN	Anastomotic leakage (OR: 0.75, 95%CI: 0.43, 1.31) Wound infection (OR 0.99, 95%CI: 0.49, 2.00) Pneumonia (OR: 1.04, 95%CI: 0.65, 1.65)
Song, G.M. (2017) [37]	Gastrectomy	11 RCTs	840	Enteral IMN	Standard EN	Infectious complications (OR: 0.56, 95%CI: 0.36, 0.86) Non-infectious complications (OR: 1.04, 95%CI: 0.52, 2.10) Length of hospital stay (MD: −0.42, 95%CI: −0.74, −0.10)
Tian, X. (2022) [36]	Esophagectomy	14 RCTs	1071	Enteral IMN	Standard EN	Infectious complications (OR: 0.44, 95%CI: 0.20, 0.98) Length of hospital stay (MD: −1.06, 95%CI: −2.56, −0.10) Anastomotic leakage (OR: 0.69, 95%CI: 0.20, 2.13) Pneumonia (OR: 0.45, 95%CI: 0.19, 0.99) Wound infection (OR 0.82, 95%CI: 0.17, 3.61)

EN—enteral nutrition; IMN—immunonutrition; MD—mean difference; OR—odds ratio; RCT—randomized controlled trials; RR—relative risk ratio.

## Data Availability

The datasets generated and/or analyzed during the current study are available upon reasonable request from the corresponding author.

## Appendix A. The Electronic Database Search Strategy

**MEDLINE via PubMed**

((“Head and Neck Neoplasms”[Mesh] OR “Head and Neck Neoplasms”[tw] OR “Head and Neck Neoplasm”[tw] OR “Ear Neoplasm”[tw] OR “Ear Neoplasms”[tw] OR “Eyelid Neoplasm”[tw] OR “Eyelid Neoplasms”[tw] OR “Facial Neoplasm”[tw] OR “Facial Neoplasms”[tw] OR “Gingival Neoplasm”[tw] OR “Gingival Neoplasms”[tw] OR “Hypopharyngeal Neoplasm”[tw] OR “Hypopharyngeal Neoplasms”[tw] OR “Laryngeal Neoplasm”[tw] OR “Laryngeal Neoplasms”[tw] OR “Lip Neoplasm”[tw] OR “Lip Neoplasms”[tw] OR “Maxillary Sinus Neoplasm”[tw] OR “Maxillary Sinus Neoplasms”[tw] OR “Mouth Neoplasm”[tw] OR “Mouth Neoplasms”[tw] OR “Nasopharyngeal Neoplasm”[tw] OR “Nasopharyngeal Neoplasms”[tw] OR “Nose Neoplasm”[tw] OR “Nose Neoplasms”[tw] OR “Oropharyngeal Neoplasm”[tw] OR “Oropharyngeal Neoplasms”[tw] OR “Otorhinolaryngologic Neoplasm”[tw] OR “Otorhinolaryngologic Neoplasms”[tw] OR “Palatal Neoplasm”[tw] OR “Palatal Neoplasms”[tw] OR “Papillary Thyroid Neoplasm “[tw] OR “Papillary Thyroid Neoplasms “[tw] OR “Paranasal Sinus Neoplasm”[tw] OR “Paranasal Sinus Neoplasms”[tw] OR “Parathyroid Neoplasm”[tw] OR “Parathyroid Neoplasms”[tw] OR “Parotid Neoplasm”[tw] OR “Parotid Neoplasms”[tw] OR “Pharyngeal Neoplasm”[tw] OR “Pharyngeal Neoplasms”[tw] OR “Salivary Gland Neoplasm”[tw] OR “Salivary Gland Neoplasms”[tw] OR “Sublingual Gland Neoplasm”[tw] OR “Sublingual Gland Neoplasms”[tw] OR “Submandibular Gland Neoplasm”[tw] OR “Submandibular Gland Neoplasms”[tw] OR “Thyroid Neoplasm”[tw] OR “Thyroid Neoplasms”[tw] OR “Tongue Neoplasm”[tw] OR “Tongue Neoplasms”[tw] OR “Tonsillar Neoplasm”[tw] OR “Tonsillar Neoplasms”[tw] OR “Tracheal Neoplasm”[tw] OR “Tracheal Neoplasms”[tw] OR “Head and Neck Cancers”[tw] OR “Head and Neck Cancer”[tw] OR “Ear Cancer”[tw] OR “Ear Cancers”[tw] OR “Eyelid Cancer”[tw] OR “Eyelid Cancers”[tw] OR “Facial Cancer”[tw] OR “Facial Cancers”[tw] OR “Gingival Cancer”[tw] OR “Gingival Cancers”[tw] OR “Hypopharyngeal Cancer”[tw] OR “Hypopharyngeal Cancers”[tw] OR “Laryngeal Cancer”[tw] OR “Laryngeal Cancers”[tw] OR “Lip Cancer”[tw] OR “Lip Cancers”[tw] OR “Maxillary Sinus Cancer”[tw] OR “Maxillary Sinus Cancers”[tw] OR “Mouth Cancer”[tw] OR “Mouth Cancers”[tw] OR “Nasopharyngeal Cancer”[tw] OR “Nasopharyngeal Cancers”[tw] OR “Nose Cancer”[tw] OR “Nose Cancers”[tw] OR “Oropharyngeal Cancer”[tw] OR “Oropharyngeal Cancers”[tw] OR “Otorhinolaryngologic Cancer”[tw] OR “Otorhinolaryngologic Cancers”[tw] OR “Palatal Cancer”[tw] OR “Palatal Cancers”[tw] OR “Papillary Thyroid Cancer “[tw] OR “Papillary Thyroid Cancers “[tw] OR “Paranasal Sinus Cancer”[tw] OR “Paranasal Sinus Cancers”[tw] OR “Parathyroid Cancer”[tw] OR “Parathyroid Cancers”[tw] OR “Parotid Cancer”[tw] OR “Parotid Cancers”[tw] OR “Pharyngeal Cancer”[tw] OR “Pharyngeal Cancers”[tw] OR “Salivary Gland Cancer”[tw] OR “Salivary Gland Cancers”[tw] OR “Sublingual Gland Cancer”[tw] OR “Sublingual Gland Cancers”[tw] OR “Submandibular Gland Cancer”[tw] OR “Submandibular Gland Cancers”[tw] OR “Thyroid Cancer”[tw] OR “Thyroid Cancers”[tw] OR “Tongue Cancer”[tw] OR “Tongue Cancers”[tw] OR “Tonsillar Cancer”[tw] OR “Tonsillar Cancers”[tw] OR “Tracheal Cancer”[tw] OR “Tracheal Cancers”[tw] OR “Head and Neck Carcinomas”[tw] OR “Head and Neck Carcinoma”[tw] OR “Ear Carcinoma”[tw] OR “Ear Carcinomas”[tw] OR “Eyelid Carcinoma”[tw] OR “Eyelid Carcinomas”[tw] OR “Facial Carcinoma”[tw] OR “Facial Carcinomas”[tw] OR “Gingival Carcinoma”[tw] OR “Gingival Carcinomas”[tw] OR “Hypopharyngeal Carcinoma”[tw] OR “Hypopharyngeal Carcinomas”[tw] OR “Laryngeal Carcinoma”[tw] OR “Laryngeal Carcinomas”[tw] OR “Lip Carcinoma”[tw] OR “Lip Carcinomas”[tw] OR “Maxillary Sinus Carcinoma”[tw] OR “Maxillary Sinus Carcinomas”[tw] OR “Mouth Carcinoma”[tw] OR “Mouth Carcinomas”[tw] OR “Nasopharyngeal Carcinoma”[tw] OR “Nasopharyngeal Carcinomas”[tw] OR “Nose Carcinoma”[tw] OR “Nose Carcinomas”[tw] OR “Oropharyngeal Carcinoma”[tw] OR “Oropharyngeal Carcinomas”[tw] OR “Otorhinolaryngologic Carcinoma”[tw] OR “Otorhinolaryngologic Carcinomas”[tw] OR “Palatal Carcinoma”[tw] OR “Palatal Carcinomas”[tw] OR “Papillary Thyroid Carcinoma “[tw] OR “Papillary Thyroid Carcinomas “[tw] OR “Paranasal Sinus Carcinoma”[tw] OR “Paranasal Sinus Carcinomas”[tw] OR “Parathyroid Carcinoma”[tw] OR “Parathyroid Carcinomas”[tw] OR “Parotid Carcinoma”[tw] OR “Parotid Carcinomas”[tw] OR “Pharyngeal Carcinoma”[tw] OR “Pharyngeal Carcinomas”[tw] OR “Salivary Gland Carcinoma”[tw] OR “Salivary Gland Carcinomas”[tw] OR “Sublingual Gland Carcinoma”[tw] OR “Sublingual Gland Carcinomas”[tw] OR “Submandibular Gland Carcinoma”[tw] OR “Submandibular Gland Carcinomas”[tw] OR “Thyroid Carcinoma”[tw] OR “Thyroid Carcinomas”[tw] OR “Tongue Carcinoma”[tw] OR “Tongue Carcinomas”[tw] OR “Tonsillar Carcinoma”[tw] OR “Tonsillar Carcinomas”[tw] OR “Tracheal Carcinoma”[tw] OR “Tracheal Carcinomas”[tw] OR “Head and Neck Adenocarcinomas”[tw] OR “Head and Neck Adenocarcinoma”[tw] OR “Ear Adenocarcinoma”[tw] OR “Ear Adenocarcinomas”[tw] OR “Eyelid Adenocarcinoma”[tw] OR “Eyelid Adenocarcinomas”[tw] OR “Facial Adenocarcinoma”[tw] OR “Facial Adenocarcinomas”[tw] OR “Gingival Adenocarcinoma”[tw] OR “Gingival Adenocarcinomas”[tw] OR “Hypopharyngeal Adenocarcinoma”[tw] OR “Hypopharyngeal Adenocarcinomas”[tw] OR “Laryngeal Adenocarcinoma”[tw] OR “Laryngeal Adenocarcinomas”[tw] OR “Lip Adenocarcinoma”[tw] OR “Lip Adenocarcinomas”[tw] OR “Maxillary Sinus Adenocarcinoma”[tw] OR “Maxillary Sinus Adenocarcinomas”[tw] OR “Mouth Adenocarcinoma”[tw] OR “Mouth Adenocarcinomas”[tw] OR “Nasopharyngeal Adenocarcinoma”[tw] OR “Nasopharyngeal Adenocarcinomas”[tw] OR “Nose Adenocarcinoma”[tw] OR “Nose Adenocarcinomas”[tw] OR “Oropharyngeal Adenocarcinoma”[tw] OR “Oropharyngeal Adenocarcinomas”[tw] OR “Otorhinolaryngologic Adenocarcinoma”[tw] OR “Otorhinolaryngologic Adenocarcinomas”[tw] OR “Palatal Adenocarcinoma”[tw] OR “Palatal Adenocarcinomas”[tw] OR “Papillary Thyroid Adenocarcinoma “[tw] OR “Papillary Thyroid Adenocarcinomas “[tw] OR “Paranasal Sinus Adenocarcinoma”[tw] OR “Paranasal Sinus Adenocarcinomas”[tw] OR “Parathyroid Adenocarcinoma”[tw] OR “Parathyroid Adenocarcinomas”[tw] OR “Parotid Adenocarcinoma”[tw] OR “Parotid Adenocarcinomas”[tw] OR “Pharyngeal Adenocarcinoma”[tw] OR “Pharyngeal Adenocarcinomas”[tw] OR “Salivary Gland Adenocarcinoma”[tw] OR “Salivary Gland Adenocarcinomas”[tw] OR “Sublingual Gland Adenocarcinoma”[tw] OR “Sublingual Gland Adenocarcinomas”[tw] OR “Submandibular Gland Adenocarcinoma”[tw] OR “Submandibular Gland Adenocarcinomas”[tw] OR “Thyroid Adenocarcinoma”[tw] OR “Thyroid Adenocarcinomas”[tw] OR “Tongue Adenocarcinoma”[tw] OR “Tongue Adenocarcinomas”[tw] OR “Tonsillar Adenocarcinoma”[tw] OR “Tonsillar Adenocarcinomas”[tw] OR “Tracheal Adenocarcinoma”[tw] OR “Tracheal Adenocarcinomas”[tw] OR “Head and Neck Tumors”[tw] OR “Head and Neck Tumor”[tw] OR “Ear Tumor”[tw] OR “Ear Tumors”[tw] OR “Eyelid Tumor”[tw] OR “Eyelid Tumors”[tw] OR “Facial Tumor”[tw] OR “Facial Tumors”[tw] OR “Gingival Tumor”[tw] OR “Gingival Tumors”[tw] OR “Hypopharyngeal Tumor”[tw] OR “Hypopharyngeal Tumors”[tw] OR “Laryngeal Tumor”[tw] OR “Laryngeal Tumors”[tw] OR “Lip Tumor”[tw] OR “Lip Tumors”[tw] OR “Maxillary Sinus Tumor”[tw] OR “Maxillary Sinus Tumors”[tw] OR “Mouth Tumor”[tw] OR “Mouth Tumors”[tw] OR “Nasopharyngeal Tumor”[tw] OR “Nasopharyngeal Tumors”[tw] OR “Nose Tumor”[tw] OR “Nose Tumors”[tw] OR “Oropharyngeal Tumor”[tw] OR “Oropharyngeal Tumors”[tw] OR “Otorhinolaryngologic Tumor”[tw] OR “Otorhinolaryngologic Tumors”[tw] OR “Palatal Tumor”[tw] OR “Palatal Tumors”[tw] OR “Papillary Thyroid Tumor “[tw] OR “Papillary Thyroid Tumors “[tw] OR “Paranasal Sinus Tumor”[tw] OR “Paranasal Sinus Tumors”[tw] OR “Parathyroid Tumor”[tw] OR “Parathyroid Tumors”[tw] OR “Parotid Tumor”[tw] OR “Parotid Tumors”[tw] OR “Pharyngeal Tumor”[tw] OR “Pharyngeal Tumors”[tw] OR “Salivary Gland Tumor”[tw] OR “Salivary Gland Tumors”[tw] OR “Sublingual Gland Tumor”[tw] OR “Sublingual Gland Tumors”[tw] OR “Submandibular Gland Tumor”[tw] OR “Submandibular Gland Tumors”[tw] OR “Thyroid Tumor”[tw] OR “Thyroid Tumors”[tw] OR “Tongue Tumor”[tw] OR “Tongue Tumors”[tw] OR “Tonsillar Tumor”[tw] OR “Tonsillar Tumors”[tw] OR “Tracheal Tumor”[tw] OR “Tracheal Tumors”[tw] OR “Head and Neck Tumours”[tw] OR “Head and Neck Tumour”[tw] OR “Ear Tumour”[tw] OR “Ear Tumours”[tw] OR “Eyelid Tumour”[tw] OR “Eyelid Tumours”[tw] OR “Facial Tumour”[tw] OR “Facial Tumours”[tw] OR “Gingival Tumour”[tw] OR “Gingival Tumours”[tw] OR “Hypopharyngeal Tumour”[tw] OR “Hypopharyngeal Tumours”[tw] OR “Laryngeal Tumour”[tw] OR “Laryngeal Tumours”[tw] OR “Lip Tumour”[tw] OR “Lip Tumours”[tw] OR “Maxillary Sinus Tumour”[tw] OR “Maxillary Sinus Tumours”[tw] OR “Mouth Tumour”[tw] OR “Mouth Tumours”[tw] OR “Nasopharyngeal Tumour”[tw] OR “Nasopharyngeal Tumours”[tw] OR “Nose Tumour”[tw] OR “Nose Tumours”[tw] OR “Oropharyngeal Tumour”[tw] OR “Oropharyngeal Tumours”[tw] OR “Otorhinolaryngologic Tumour”[tw] OR “Otorhinolaryngologic Tumours”[tw] OR “Palatal Tumour”[tw] OR “Palatal Tumours”[tw] OR “Papillary Thyroid Tumour “[tw] OR “Papillary Thyroid Tumours “[tw] OR “Paranasal Sinus Tumour”[tw] OR “Paranasal Sinus Tumours”[tw] OR “Parathyroid Tumour”[tw] OR “Parathyroid Tumours”[tw] OR “Parotid Tumour”[tw] OR “Parotid Tumours”[tw] OR “Pharyngeal Tumour”[tw] OR “Pharyngeal Tumours”[tw] OR “Salivary Gland Tumour”[tw] OR “Salivary Gland Tumours”[tw] OR “Sublingual Gland Tumour”[tw] OR “Sublingual Gland Tumours”[tw] OR “Submandibular Gland Tumour”[tw] OR “Submandibular Gland Tumours”[tw] OR “Thyroid Tumour”[tw] OR “Thyroid Tumours”[tw] OR “Tongue Tumour”[tw] OR “Tongue Tumours”[tw] OR “Tonsillar Tumour”[tw] OR “Tonsillar Tumours”[tw] OR “Tracheal Tumour”[tw] OR “Tracheal Tumours”[tw] OR “Head and Neck Malignancies”[tw] OR “Head and Neck Malignancy”[tw] OR “Ear Malignancy”[tw] OR “Ear Malignancies”[tw] OR “Eyelid Malignancy”[tw] OR “Eyelid Malignancies”[tw] OR “Facial Malignancy”[tw] OR “Facial Malignancies”[tw] OR “Gingival Malignancy”[tw] OR “Gingival Malignancies”[tw] OR “Hypopharyngeal Malignancy”[tw] OR “Hypopharyngeal Malignancies”[tw] OR “Laryngeal Malignancy”[tw] OR “Laryngeal Malignancies”[tw] OR “Lip Malignancy”[tw] OR “Lip Malignancies”[tw] OR “Maxillary Sinus Malignancy”[tw] OR “Maxillary Sinus Malignancies”[tw] OR “Mouth Malignancy”[tw] OR “Mouth Malignancies”[tw] OR “Nasopharyngeal Malignancy”[tw] OR “Nasopharyngeal Malignancies”[tw] OR “Nose Malignancy”[tw] OR “Nose Malignancies”[tw] OR “Oropharyngeal Malignancy”[tw] OR “Oropharyngeal Malignancies”[tw] OR “Otorhinolaryngologic Malignancy”[tw] OR “Otorhinolaryngologic Malignancies”[tw] OR “Palatal Malignancy”[tw] OR “Palatal Malignancies”[tw] OR “Papillary Thyroid Malignancy “[tw] OR “Papillary Thyroid Malignancies “[tw] OR “Paranasal Sinus Malignancy”[tw] OR “Paranasal Sinus Malignancies”[tw] OR “Parathyroid Malignancy”[tw] OR “Parathyroid Malignancies”[tw] OR “Parotid Malignancy”[tw] OR “Parotid Malignancies”[tw] OR “Pharyngeal Malignancy”[tw] OR “Pharyngeal Malignancies”[tw] OR “Salivary Gland Malignancy”[tw] OR “Salivary Gland Malignancies”[tw] OR “Sublingual Gland Malignancy”[tw] OR “Sublingual Gland Malignancies”[tw] OR “Submandibular Gland Malignancy”[tw] OR “Submandibular Gland Malignancies”[tw] OR “Thyroid Malignancy”[tw] OR “Thyroid Malignancies”[tw] OR “Tongue Malignancy”[tw] OR “Tongue Malignancies”[tw] OR “Tonsillar Malignancy”[tw] OR “Tonsillar Malignancies”[tw] OR “Tracheal Malignancy”[tw] OR “Tracheal Malignancies”[tw] OR “Thyroid Nodule”[tw] OR “Oral Leukoplakia”[tw] OR “Hairy Leukoplakia”[tw] OR “Gastrointestinal Neoplasms”[Mesh] OR “Gastrointestinal Neoplasms”[tw] OR “Gastrointestinal Neoplasm”[tw] OR “Stomach Neoplasms”[tw] OR “Stomach Neoplasm”[tw] OR “Gastric Neoplasms”[tw] OR “Gastric Neoplasm”[tw] OR “Esophageal Neoplasms”[tw] OR “Esophageal Neoplasm”[tw] OR “OEsophageal Neoplasms”[tw] OR “OEsophageal Neoplasm”[tw] OR “Esophagus Neoplasms”[tw] OR “Esophagus Neoplasm”[tw] OR “OEsophagus Neoplasms”[tw] OR “Esophagus Neoplasm”[tw] OR “Esophageal Squamus Cell Neoplasms”[tw] OR “Esophageal Squamus Cell Neoplasm”[tw] OR “OEsophageal Squamus Cell Neoplasms”[tw] OR “OEsophageal Squamus Cell Neoplasm”[tw] OR “Esophagus Squamus Cell Neoplasms”[tw] OR “Esophagus Squamus Cell Neoplasm”[tw] OR “OEsophagus Squamus Cell Neoplasms”[tw] OR “Esophagus Squamus Cell Neoplasm”[tw] OR “Intestinal Neoplasms”[tw] OR “Intestinal Neoplasm”[tw] OR “Cecal Neoplasms”[tw] OR “Cecal Neoplasm”[tw] OR “Appenciceal Neoplasms”[tw] OR “Appendiceal Neoplasm”[tw] OR “Colorectal Neoplasms”[tw] OR “Colorectal Neoplasm”[tw] OR “Colonic Neoplasms”[tw] OR “Colonic Neoplasm”[tw] OR “Colon Neoplasms”[tw] OR “Colon Neoplasm”[tw] OR “Sigmoidal Neoplasms”[tw] OR “Sigmoidal Neoplasm”[tw] OR “Sigmoid Neoplasms”[tw] OR “Sigmoid Neoplasm”[tw] OR “Rectal Neoplasms”[tw] OR “Rectal Neoplasm”[tw] OR “Anorectal Neoplasms”[tw] OR “Anorectal Neoplasm”[tw] OR “Anus Neoplasms”[tw] OR “Anus Neoplasm”[tw] OR “Anal Neoplasms”[tw] OR “Anal Neoplasm”[tw] OR “Anal Gland Neoplasms”[tw] OR “Anal Gland Neoplasm”[tw] OR “Duodenal Neoplasms”[tw] OR “Duodenal Neoplasm”[tw] OR “Duodenum Neoplasms”[tw] OR “Duodenum Neoplasm”[tw] OR “Ileal Neoplasms”[tw] OR “Ileal Neoplasm”[tw] OR “Ileum Neoplasms”[tw] OR “Ileum Neoplasm”[tw] OR “Jejunal Neoplasms”[tw] OR “Jejunal Neoplasm”[tw] OR “Jejunum Neoplasms”[tw] OR “Jejunum Neoplasm”[tw] OR “Gastrointestinal Cancers”[tw] OR “Gastrointestinal Cancer”[tw] OR “Stomach Cancers”[tw] OR “Stomach Cancer”[tw] OR “Gastric Cancers”[tw] OR “Gastric Cancer”[tw] OR “Esophageal Cancers”[tw] OR “Esophageal Cancer”[tw] OR “OEsophageal Cancers”[tw] OR “OEsophageal Cancer”[tw] OR “Esophagus Cancers”[tw] OR “Esophagus Cancer”[tw] OR “OEsophagus Cancers”[tw] OR “Esophagus Cancer”[tw] OR “Esophageal Squamus Cell Cancers”[tw] OR “Esophageal Squamus Cell Cancer”[tw] OR “OEsophageal Squamus Cell Cancers”[tw] OR “OEsophageal Squamus Cell Cancer”[tw] OR “Esophagus Squamus Cell Cancers”[tw] OR “Esophagus Squamus Cell Cancer”[tw] OR “OEsophagus Squamus Cell Cancers”[tw] OR “Esophagus Squamus Cell Cancer”[tw] OR “Intestinal Cancers”[tw] OR “Intestinal Cancer”[tw] OR “Cecal Cancers”[tw] OR “Cecal Cancer”[tw] OR “Appenciceal Cancers”[tw] OR “Appendiceal Cancer”[tw] OR “Colorectal Cancers”[tw] OR “Colorectal Cancer”[tw] OR “Colonic Cancers”[tw] OR “Colonic Cancer”[tw] OR “Colon Cancers”[tw] OR “Colon Cancer”[tw] OR “Sigmoidal Cancers”[tw] OR “Sigmoidal Cancer”[tw] OR “Sigmoid Cancers”[tw] OR “Sigmoid Cancer”[tw] OR “Rectal Cancers”[tw] OR “Rectal Cancer”[tw] OR “Anorectal Cancers”[tw] OR “Anorectal Cancer”[tw] OR “Anus Cancers”[tw] OR “Anus Cancer”[tw] OR “Anal Cancers”[tw] OR “Anal Cancer”[tw] OR “Anal Gland Cancers”[tw] OR “Anal Gland Cancer”[tw] OR “Duodenal Cancers”[tw] OR “Duodenal Cancer”[tw] OR “Duodenum Cancers”[tw] OR “Duodenum Cancer”[tw] OR “Ileal Cancers”[tw] OR “Ileal Cancer”[tw] OR “Ileum Cancers”[tw] OR “Ileum Cancer”[tw] OR “Jejunal Cancers”[tw] OR “Jejunal Cancer”[tw] OR “Jejunum Cancers”[tw] OR “Jejunum Cancer”[tw] OR “Gastrointestinal Carcinomas”[tw] OR “Gastrointestinal Carcinoma”[tw] OR “Stomach Carcinomas”[tw] OR “Stomach Carcinoma”[tw] OR “Gastric Carcinomas”[tw] OR “Gastric Carcinoma”[tw] OR “Esophageal Carcinomas”[tw] OR “Esophageal Carcinoma”[tw] OR “OEsophageal Carcinomas”[tw] OR “OEsophageal Carcinoma”[tw] OR “Esophagus Carcinomas”[tw] OR “Esophagus Carcinoma”[tw] OR “OEsophagus Carcinomas”[tw] OR “Esophagus Carcinoma”[tw] OR “Esophageal Squamus Cell Carcinomas”[tw] OR “Esophageal Squamus Cell Carcinoma”[tw] OR “OEsophageal Squamus Cell Carcinomas”[tw] OR “OEsophageal Squamus Cell Carcinoma”[tw] OR “Esophagus Squamus Cell Carcinomas”[tw] OR “Esophagus Squamus Cell Carcinoma”[tw] OR “OEsophagus Squamus Cell Carcinomas”[tw] OR “Esophagus Squamus Cell Carcinoma”[tw] OR “Intestinal Carcinomas”[tw] OR “Intestinal Carcinoma”[tw] OR “Cecal Carcinomas”[tw] OR “Cecal Carcinoma”[tw] OR “Appenciceal Carcinomas”[tw] OR “Appendiceal Carcinoma”[tw] OR “Colorectal Carcinomas”[tw] OR “Colorectal Carcinoma”[tw] OR “Colonic Carcinomas”[tw] OR “Colonic Carcinoma”[tw] OR “Colon Carcinomas”[tw] OR “Colon Carcinoma”[tw] OR “Sigmoidal Carcinomas”[tw] OR “Sigmoidal Carcinoma”[tw] OR “Sigmoid Carcinomas”[tw] OR “Sigmoid Carcinoma”[tw] OR “Rectal Carcinomas”[tw] OR “Rectal Carcinoma”[tw] OR “Anorectal Carcinomas”[tw] OR “Anorectal Carcinoma”[tw] OR “Anus Carcinomas”[tw] OR “Anus Carcinoma”[tw] OR “Anal Carcinomas”[tw] OR “Anal Carcinoma”[tw] OR “Anal Gland Carcinomas”[tw] OR “Anal Gland Carcinoma”[tw] OR “Duodenal Carcinomas”[tw] OR “Duodenal Carcinoma”[tw] OR “Duodenum Carcinomas”[tw] OR “Duodenum Carcinoma”[tw] OR “Ileal Carcinomas”[tw] OR “Ileal Carcinoma”[tw] OR “Ileum Carcinomas”[tw] OR “Ileum Carcinoma”[tw] OR “Jejunal Carcinomas”[tw] OR “Jejunal Carcinoma”[tw] OR “Jejunum Carcinomas”[tw] OR “Jejunum Carcinoma”[tw] OR “Gastrointestinal Adenocarcinomas”[tw] OR “Gastrointestinal Adenocarcinoma”[tw] OR “Stomach Adenocarcinomas”[tw] OR “Stomach Adenocarcinoma”[tw] OR “Gastric Adenocarcinomas”[tw] OR “Gastric Adenocarcinoma”[tw] OR “Esophageal Adenocarcinomas”[tw] OR “Esophageal Adenocarcinoma”[tw] OR “OEsophageal Adenocarcinomas”[tw] OR “OEsophageal Adenocarcinoma”[tw] OR “Esophagus Adenocarcinomas”[tw] OR “Esophagus Adenocarcinoma”[tw] OR “OEsophagus Adenocarcinomas”[tw] OR “Esophagus Adenocarcinoma”[tw] OR “Esophageal Squamus Cell Adenocarcinomas”[tw] OR “Esophageal Squamus Cell Adenocarcinoma”[tw] OR “OEsophageal Squamus Cell Adenocarcinomas”[tw] OR “OEsophageal Squamus Cell Adenocarcinoma”[tw] OR “Esophagus Squamus Cell Adenocarcinomas”[tw] OR “Esophagus Squamus Cell Adenocarcinoma”[tw] OR “OEsophagus Squamus Cell Adenocarcinomas”[tw] OR “Esophagus Squamus Cell Adenocarcinoma”[tw] OR “Intestinal Adenocarcinomas”[tw] OR “Intestinal Adenocarcinoma”[tw] OR “Cecal Adenocarcinomas”[tw] OR “Cecal Adenocarcinoma”[tw] OR “Appenciceal Adenocarcinomas”[tw] OR “Appendiceal Adenocarcinoma”[tw] OR “Colorectal Adenocarcinomas”[tw] OR “Colorectal Adenocarcinoma”[tw] OR “Colonic Adenocarcinomas”[tw] OR “Colonic Adenocarcinoma”[tw] OR “Colon Adenocarcinomas”[tw] OR “Colon Adenocarcinoma”[tw] OR “Sigmoidal Adenocarcinomas”[tw] OR “Sigmoidal Adenocarcinoma”[tw] OR “Sigmoid Adenocarcinomas”[tw] OR “Sigmoid Adenocarcinoma”[tw] OR “Rectal Adenocarcinomas”[tw] OR “Rectal Adenocarcinoma”[tw] OR “Anorectal Adenocarcinomas”[tw] OR “Anorectal Adenocarcinoma”[tw] OR “Anus Adenocarcinomas”[tw] OR “Anus Adenocarcinoma”[tw] OR “Anal Adenocarcinomas”[tw] OR “Anal Adenocarcinoma”[tw] OR “Anal Gland Adenocarcinomas”[tw] OR “Anal Gland Adenocarcinoma”[tw] OR “Duodenal Adenocarcinomas”[tw] OR “Duodenal Adenocarcinoma”[tw] OR “Duodenum Adenocarcinomas”[tw] OR “Duodenum Adenocarcinoma”[tw] OR “Ileal Adenocarcinomas”[tw] OR “Ileal Adenocarcinoma”[tw] OR “Ileum Adenocarcinomas”[tw] OR “Ileum Adenocarcinoma”[tw] OR “Jejunal Adenocarcinomas”[tw] OR “Jejunal Adenocarcinoma”[tw] OR “Jejunum Adenocarcinomas”[tw] OR “Jejunum Adenocarcinoma”[tw] OR “Gastrointestinal Tumors”[tw] OR “Gastrointestinal Tumor”[tw] OR “Stomach Tumors”[tw] OR “Stomach Tumor”[tw] OR “Gastric Tumors”[tw] OR “Gastric Tumor”[tw] OR “Esophageal Tumors”[tw] OR “Esophageal Tumor”[tw] OR “OEsophageal Tumors”[tw] OR “OEsophageal Tumor”[tw] OR “Esophagus Tumors”[tw] OR “Esophagus Tumor”[tw] OR “OEsophagus Tumors”[tw] OR “Esophagus Tumor”[tw] OR “Esophageal Squamus Cell Tumors”[tw] OR “Esophageal Squamus Cell Tumor”[tw] OR “OEsophageal Squamus Cell Tumors”[tw] OR “OEsophageal Squamus Cell Tumor”[tw] OR “Esophagus Squamus Cell Tumors”[tw] OR “Esophagus Squamus Cell Tumor”[tw] OR “OEsophagus Squamus Cell Tumors”[tw] OR “Esophagus Squamus Cell Tumor”[tw] OR “Intestinal Tumors”[tw] OR “Intestinal Tumor”[tw] OR “Cecal Tumors”[tw] OR “Cecal Tumor”[tw] OR “Appenciceal Tumors”[tw] OR “Appendiceal Tumor”[tw] OR “Colorectal Tumors”[tw] OR “Colorectal Tumor”[tw] OR “Colonic Tumors”[tw] OR “Colonic Tumor”[tw] OR “Colon Tumors”[tw] OR “Colon Tumor”[tw] OR “Sigmoidal Tumors”[tw] OR “Sigmoidal Tumor”[tw] OR “Sigmoid Tumors”[tw] OR “Sigmoid Tumor”[tw] OR “Rectal Tumors”[tw] OR “Rectal Tumor”[tw] OR “Anorectal Tumors”[tw] OR “Anorectal Tumor”[tw] OR “Anus Tumors”[tw] OR “Anus Tumor”[tw] OR “Anal Tumors”[tw] OR “Anal Tumor”[tw] OR “Anal Gland Tumors”[tw] OR “Anal Gland Tumor”[tw] OR “Duodenal Tumors”[tw] OR “Duodenal Tumor”[tw] OR “Duodenum Tumors”[tw] OR “Duodenum Tumor”[tw] OR “Ileal Tumors”[tw] OR “Ileal Tumor”[tw] OR “Ileum Tumors”[tw] OR “Ileum Tumor”[tw] OR “Jejunal Tumors”[tw] OR “Jejunal Tumor”[tw] OR “Jejunum Tumors”[tw] OR “Jejunum Tumor”[tw] OR “Gastrointestinal Tumours”[tw] OR “Gastrointestinal Tumour”[tw] OR “Stomach Tumours”[tw] OR “Stomach Tumour”[tw] OR “Gastric Tumours”[tw] OR “Gastric Tumour”[tw] OR “Esophageal Tumours”[tw] OR “Esophageal Tumour”[tw] OR “OEsophageal Tumours”[tw] OR “OEsophageal Tumour”[tw] OR “Esophagus Tumours”[tw] OR “Esophagus Tumour”[tw] OR “OEsophagus Tumours”[tw] OR “Esophagus Tumour”[tw] OR “Esophageal Squamus Cell Tumours”[tw] OR “Esophageal Squamus Cell Tumour”[tw] OR “OEsophageal Squamus Cell Tumours”[tw] OR “OEsophageal Squamus Cell Tumour”[tw] OR “Esophagus Squamus Cell Tumours”[tw] OR “Esophagus Squamus Cell Tumour”[tw] OR “OEsophagus Squamus Cell Tumours”[tw] OR “Esophagus Squamus Cell Tumour”[tw] OR “Intestinal Tumours”[tw] OR “Intestinal Tumour”[tw] OR “Cecal Tumours”[tw] OR “Cecal Tumour”[tw] OR “Appenciceal Tumours”[tw] OR “Appendiceal Tumour”[tw] OR “Colorectal Tumours”[tw] OR “Colorectal Tumour”[tw] OR “Colonic Tumours”[tw] OR “Colonic Tumour”[tw] OR “Colon Tumours”[tw] OR “Colon Tumour”[tw] OR “Sigmoidal Tumours”[tw] OR “Sigmoidal Tumour”[tw] OR “Sigmoid Tumours”[tw] OR “Sigmoid Tumour”[tw] OR “Rectal Tumours”[tw] OR “Rectal Tumour”[tw] OR “Anorectal Tumours”[tw] OR “Anorectal Tumour”[tw] OR “Anus Tumours”[tw] OR “Anus Tumour”[tw] OR “Anal Tumours”[tw] OR “Anal Tumour”[tw] OR “Anal Gland Tumours”[tw] OR “Anal Gland Tumour”[tw] OR “Duodenal Tumours”[tw] OR “Duodenal Tumour”[tw] OR “Duodenum Tumours”[tw] OR “Duodenum Tumour”[tw] OR “Ileal Tumours”[tw] OR “Ileal Tumour”[tw] OR “Ileum Tumours”[tw] OR “Ileum Tumour”[tw] OR “Jejunal Tumours”[tw] OR “Jejunal Tumour”[tw] OR “Jejunum Tumours”[tw] OR “Jejunum Tumour”[tw] OR “Gastrointestinal Malignancies”[tw] OR “Gastrointestinal Malignancy”[tw] OR “Stomach Malignancies”[tw] OR “Stomach Malignancy”[tw] OR “Gastric Malignancies”[tw] OR “Gastric Malignancy”[tw] OR “Esophageal Malignancies”[tw] OR “Esophageal Malignancy”[tw] OR “OEsophageal Malignancies”[tw] OR “OEsophageal Malignancy”[tw] OR “Esophagus Malignancies”[tw] OR “Esophagus Malignancy”[tw] OR “OEsophagus Malignancies”[tw] OR “Esophagus Malignancy”[tw] OR “Esophageal Squamus Cell Malignancies”[tw] OR “Esophageal Squamus Cell Malignancy”[tw] OR “OEsophageal Squamus Cell Malignancies”[tw] OR “OEsophageal Squamus Cell Malignancy”[tw] OR “Esophagus Squamus Cell Malignancies”[tw] OR “Esophagus Squamus Cell Malignancy”[tw] OR “OEsophagus Squamus Cell Malignancies”[tw] OR “Esophagus Squamus Cell Malignancy”[tw] OR “Intestinal Malignancies”[tw] OR “Intestinal Malignancy”[tw] OR “Cecal Malignancies”[tw] OR “Cecal Malignancy”[tw] OR “Appenciceal Malignancies”[tw] OR “Appendiceal Malignancy”[tw] OR “Colorectal Malignancies”[tw] OR “Colorectal Malignancy”[tw] OR “Colonic Malignancies”[tw] OR “Colonic Malignancy”[tw] OR “Colon Malignancies”[tw] OR “Colon Malignancy”[tw] OR “Sigmoidal Malignancies”[tw] OR “Sigmoidal Malignancy”[tw] OR “Sigmoid Malignancies”[tw] OR “Sigmoid Malignancy”[tw] OR “Rectal Malignancies”[tw] OR “Rectal Malignancy”[tw] OR “Anorectal Malignancies”[tw] OR “Anorectal Malignancy”[tw] OR “Anus Malignancies”[tw] OR “Anus Malignancy”[tw] OR “Anal Malignancies”[tw] OR “Anal Malignancy”[tw] OR “Anal Gland Malignancies”[tw] OR “Anal Gland Malignancy”[tw] OR “Duodenal Malignancies”[tw] OR “Duodenal Malignancy”[tw] OR “Duodenum Malignancies”[tw] OR “Duodenum Malignancy”[tw] OR “Ileal Malignancies”[tw] OR “Ileal Malignancy”[tw] OR “Ileum Malignancies”[tw] OR “Ileum Malignancy”[tw] OR “Jejunal Malignancies”[tw] OR “Jejunal Malignancy”[tw] OR “Jejunum Malignancies”[tw] OR “Jejunum Malignancy”[tw] OR “Adenomatous Polyposis Coli”[tw] OR “Gardner Syndrome”[tw]) AND (“resection”[tw] OR “resect*”[tw] OR “surgery”[Subheading] OR “surgery”[tw] OR “surgical procedures, operative”[mesh] OR “surgical”[tw] OR “gastrectomy”[mesh] OR “gastrectomy”[tw] OR “gastrectom*”[tw] OR “Preoperative Period”[Mesh] OR “Preoperative Care”[Mesh] OR “Preoperative Period”[tw] OR “Preoperative Care”[tw] OR “Perioperative Period”[mesh] OR “Perioperative Care”[mesh] OR “Perioperative Period”[tw] OR “Perioperative Care”[tw] OR “Postoperative Period”[Mesh] OR “Postoperative Care”[Mesh] OR “Postoperative Period”[tw] OR “Postoperative Care”[tw] OR “Preoperative”[tw] OR “Pre operative”[tw] OR “Perioperative”[tw] OR “Peri operative”[tw] OR “Postoperative”[tw] OR “Post operative”[tw]) AND (“immunonutrition”[tw] OR “immunonutr*”[tw] OR “immuno nutrition”[tw] OR “immuno nutr*”[tw] OR ((“Immunomodulation”[mesh] OR “Immunomodulation”[tw] OR “Immunomodulat*”[tw] OR “Immunotherapy”[mesh] OR “immunother*”[tw] OR “ Malnutrition/immunology”[mesh]) AND (“Nutrition Therapy”[Mesh] OR “Nutrition Therapy”[tw] OR “Diet Therapy”[mesh] OR “Diet Therapy”[tw] OR “Caloric Restriction”[tw] OR “Carbohydrate Loading diet”[tw] OR “Carbohydrate-Restricted diet”[tw] OR “High-Protein Low-Carbohydrate diet”[tw] OR “Ketogenic diet”[tw] OR “Diabetic diet”[tw] OR “Fat-Restricted diet”[tw] OR “Gluten-Free diet”[tw] OR “High-Protein diet”[tw] OR “High-Protein Low-Carbohydrate diet”[tw] OR “Mediterranean diet”[tw] OR “Paleolithic diet”[tw] OR “Protein-Restricted diet”[tw] OR “Reducing diet”[tw] OR “Sodium-Restricted diet”[tw] OR “Vegetarian diet”[tw] OR “Macrobiotic diet”[tw] OR “Vegan diet”[tw] OR “Carbohydrate Loading diets”[tw] OR “Carbohydrate-Restricted diets”[tw] OR “High-Protein Low-Carbohydrate diets”[tw] OR “Ketogenic diets”[tw] OR “Diabetic diets”[tw] OR “Fat-Restricted diets”[tw] OR “Gluten-Free diets”[tw] OR “High-Protein diets”[tw] OR “High-Protein Low-Carbohydrate diets”[tw] OR “Mediterranean diets”[tw] OR “Paleolithic diets”[tw] OR “Protein-Restricted diets”[tw] OR “Reducing diets”[tw] OR “Sodium-Restricted diets”[tw] OR “Vegetarian diets”[tw] OR “Macrobiotic diets”[tw] OR “Vegan diets”[tw] OR “Nutritional Support”[tw] OR “Enteral Nutrition”[tw] OR “Parenteral Nutrition”[tw] OR “Enteral Feeding”[tw] OR “Parenteral Feeding”[tw] OR “nutritional supplementation”[tw] OR “artifical nutrition”[tw] OR “Diet, Food, and Nutrition”[Mesh]))) AND (“clinical trial”[pt] OR “clinical trial”[tiab] OR “clinical trials as topic”[mesh] OR “clinical trials”[tiab] OR “control groups”[mesh] OR “control group”[tiab] OR “control groups”[tiab] OR “controlled clinical trial”[pt] OR “controlled clinical trials as topic”[mesh] OR “cross-over studies”[mesh] OR “cross over study”[tiab] OR “cross over studies”[tiab] OR “double-blind method”[mesh] OR “double blind”[tiab] OR “evaluation studies as topic”[mesh] OR “follow-up studies”[mesh] OR “follow up study”[tiab] OR “follow up studies”[tiab] OR “placebos”[mesh] OR placebo*[tiab] OR placebos*[tiab] OR “pragmatic clinical trial”[pt] OR “prospective studies”[mesh] OR “prospective study”[tiab] OR “prospective studies”[tiab] OR “RaCT”[tiab] OR “RaCTs”[tiab] OR “random allocation”[mesh] OR “randomised “[tiab] OR “randomized controlled trial”[pt] OR “randomized controlled trials as topic”[mesh] OR “randomized”[tiab] OR random*[tiab] OR “RCT”[tiab] OR “RCTs”[tiab] OR “Research Design”[MeSH:noexp] OR “Research design”[tiab] OR “Research designs”[tiab] OR “single blind”[tiab] OR “single-blind method”[mesh] OR ((single*[tiab] OR double*[tiab] OR triple*[tiab]) AND (blind*[tiab] OR mask*[tiab])) OR volunteer*[tiab] OR “trial”[ti] OR “trials”[ti]))

**MEDLINE via OVID**

((exp “Head and Neck Neoplasms”/ OR “Head and Neck Neoplasms”.mp OR “Head and Neck Neoplasm”.mp OR “Ear Neoplasm”.mp OR “Ear Neoplasms”.mp OR “Eyelid Neoplasm”.mp OR “Eyelid Neoplasms”.mp OR “Facial Neoplasm”.mp OR “Facial Neoplasms”.mp OR “Gingival Neoplasm”.mp OR “Gingival Neoplasms”.mp OR “Hypopharyngeal Neoplasm”.mp OR “Hypopharyngeal Neoplasms”.mp OR “Laryngeal Neoplasm”.mp OR “Laryngeal Neoplasms”.mp OR “Lip Neoplasm”.mp OR “Lip Neoplasms”.mp OR “Maxillary Sinus Neoplasm”.mp OR “Maxillary Sinus Neoplasms”.mp OR “Mouth Neoplasm”.mp OR “Mouth Neoplasms”.mp OR “Nasopharyngeal Neoplasm”.mp OR “Nasopharyngeal Neoplasms”.mp OR “Nose Neoplasm”.mp OR “Nose Neoplasms”.mp OR “Oropharyngeal Neoplasm”.mp OR “Oropharyngeal Neoplasms”.mp OR “Otorhinolaryngologic Neoplasm”.mp OR “Otorhinolaryngologic Neoplasms”.mp OR “Palatal Neoplasm”.mp OR “Palatal Neoplasms”.mp OR “Papillary Thyroid Neoplasm “.mp OR “Papillary Thyroid Neoplasms “.mp OR “Paranasal Sinus Neoplasm”.mp OR “Paranasal Sinus Neoplasms”.mp OR “Parathyroid Neoplasm”.mp OR “Parathyroid Neoplasms”.mp OR “Parotid Neoplasm”.mp OR “Parotid Neoplasms”.mp OR “Pharyngeal Neoplasm”.mp OR “Pharyngeal Neoplasms”.mp OR “Salivary Gland Neoplasm”.mp OR “Salivary Gland Neoplasms”.mp OR “Sublingual Gland Neoplasm”.mp OR “Sublingual Gland Neoplasms”.mp OR “Submandibular Gland Neoplasm”.mp OR “Submandibular Gland Neoplasms”.mp OR “Thyroid Neoplasm”.mp OR “Thyroid Neoplasms”.mp OR “Tongue Neoplasm”.mp OR “Tongue Neoplasms”.mp OR “Tonsillar Neoplasm”.mp OR “Tonsillar Neoplasms”.mp OR “Tracheal Neoplasm”.mp OR “Tracheal Neoplasms”.mp OR “Head and Neck Cancers”.mp OR “Head and Neck Cancer”.mp OR “Ear Cancer”.mp OR “Ear Cancers”.mp OR “Eyelid Cancer”.mp OR “Eyelid Cancers”.mp OR “Facial Cancer”.mp OR “Facial Cancers”.mp OR “Gingival Cancer”.mp OR “Gingival Cancers”.mp OR “Hypopharyngeal Cancer”.mp OR “Hypopharyngeal Cancers”.mp OR “Laryngeal Cancer”.mp OR “Laryngeal Cancers”.mp OR “Lip Cancer”.mp OR “Lip Cancers”.mp OR “Maxillary Sinus Cancer”.mp OR “Maxillary Sinus Cancers”.mp OR “Mouth Cancer”.mp OR “Mouth Cancers”.mp OR “Nasopharyngeal Cancer”.mp OR “Nasopharyngeal Cancers”.mp OR “Nose Cancer”.mp OR “Nose Cancers”.mp OR “Oropharyngeal Cancer”.mp OR “Oropharyngeal Cancers”.mp OR “Otorhinolaryngologic Cancer”.mp OR “Otorhinolaryngologic Cancers”.mp OR “Palatal Cancer”.mp OR “Palatal Cancers”.mp OR “Papillary Thyroid Cancer “.mp OR “Papillary Thyroid Cancers “.mp OR “Paranasal Sinus Cancer”.mp OR “Paranasal Sinus Cancers”.mp OR “Parathyroid Cancer”.mp OR “Parathyroid Cancers”.mp OR “Parotid Cancer”.mp OR “Parotid Cancers”.mp OR “Pharyngeal Cancer”.mp OR “Pharyngeal Cancers”.mp OR “Salivary Gland Cancer”.mp OR “Salivary Gland Cancers”.mp OR “Sublingual Gland Cancer”.mp OR “Sublingual Gland Cancers”.mp OR “Submandibular Gland Cancer”.mp OR “Submandibular Gland Cancers”.mp OR “Thyroid Cancer”.mp OR “Thyroid Cancers”.mp OR “Tongue Cancer”.mp OR “Tongue Cancers”.mp OR “Tonsillar Cancer”.mp OR “Tonsillar Cancers”.mp OR “Tracheal Cancer”.mp OR “Tracheal Cancers”.mp OR “Head and Neck Carcinomas”.mp OR “Head and Neck Carcinoma”.mp OR “Ear Carcinoma”.mp OR “Ear Carcinomas”.mp OR “Eyelid Carcinoma”.mp OR “Eyelid Carcinomas”.mp OR “Facial Carcinoma”.mp OR “Facial Carcinomas”.mp OR “Gingival Carcinoma”.mp OR “Gingival Carcinomas”.mp OR “Hypopharyngeal Carcinoma”.mp OR “Hypopharyngeal Carcinomas”.mp OR “Laryngeal Carcinoma”.mp OR “Laryngeal Carcinomas”.mp OR “Lip Carcinoma”.mp OR “Lip Carcinomas”.mp OR “Maxillary Sinus Carcinoma”.mp OR “Maxillary Sinus Carcinomas”.mp OR “Mouth Carcinoma”.mp OR “Mouth Carcinomas”.mp OR “Nasopharyngeal Carcinoma”.mp OR “Nasopharyngeal Carcinomas”.mp OR “Nose Carcinoma”.mp OR “Nose Carcinomas”.mp OR “Oropharyngeal Carcinoma”.mp OR “Oropharyngeal Carcinomas”.mp OR “Otorhinolaryngologic Carcinoma”.mp OR “Otorhinolaryngologic Carcinomas”.mp OR “Palatal Carcinoma”.mp OR “Palatal Carcinomas”.mp OR “Papillary Thyroid Carcinoma “.mp OR “Papillary Thyroid Carcinomas “.mp OR “Paranasal Sinus Carcinoma”.mp OR “Paranasal Sinus Carcinomas”.mp OR “Parathyroid Carcinoma”.mp OR “Parathyroid Carcinomas”.mp OR “Parotid Carcinoma”.mp OR “Parotid Carcinomas”.mp OR “Pharyngeal Carcinoma”.mp OR “Pharyngeal Carcinomas”.mp OR “Salivary Gland Carcinoma”.mp OR “Salivary Gland Carcinomas”.mp OR “Sublingual Gland Carcinoma”.mp OR “Sublingual Gland Carcinomas”.mp OR “Submandibular Gland Carcinoma”.mp OR “Submandibular Gland Carcinomas”.mp OR “Thyroid Carcinoma”.mp OR “Thyroid Carcinomas”.mp OR “Tongue Carcinoma”.mp OR “Tongue Carcinomas”.mp OR “Tonsillar Carcinoma”.mp OR “Tonsillar Carcinomas”.mp OR “Tracheal Carcinoma”.mp OR “Tracheal Carcinomas”.mp OR “Head and Neck Adenocarcinomas”.mp OR “Head and Neck Adenocarcinoma”.mp OR “Ear Adenocarcinoma”.mp OR “Ear Adenocarcinomas”.mp OR “Eyelid Adenocarcinoma”.mp OR “Eyelid Adenocarcinomas”.mp OR “Facial Adenocarcinoma”.mp OR “Facial Adenocarcinomas”.mp OR “Gingival Adenocarcinoma”.mp OR “Gingival Adenocarcinomas”.mp OR “Hypopharyngeal Adenocarcinoma”.mp OR “Hypopharyngeal Adenocarcinomas”.mp OR “Laryngeal Adenocarcinoma”.mp OR “Laryngeal Adenocarcinomas”.mp OR “Lip Adenocarcinoma”.mp OR “Lip Adenocarcinomas”.mp OR “Maxillary Sinus Adenocarcinoma”.mp OR “Maxillary Sinus Adenocarcinomas”.mp OR “Mouth Adenocarcinoma”.mp OR “Mouth Adenocarcinomas”.mp OR “Nasopharyngeal Adenocarcinoma”.mp OR “Nasopharyngeal Adenocarcinomas”.mp OR “Nose Adenocarcinoma”.mp OR “Nose Adenocarcinomas”.mp OR “Oropharyngeal Adenocarcinoma”.mp OR “Oropharyngeal Adenocarcinomas”.mp OR “Otorhinolaryngologic Adenocarcinoma”.mp OR “Otorhinolaryngologic Adenocarcinomas”.mp OR “Palatal Adenocarcinoma”.mp OR “Palatal Adenocarcinomas”.mp OR “Papillary Thyroid Adenocarcinoma “.mp OR “Papillary Thyroid Adenocarcinomas “.mp OR “Paranasal Sinus Adenocarcinoma”.mp OR “Paranasal Sinus Adenocarcinomas”.mp OR “Parathyroid Adenocarcinoma”.mp OR “Parathyroid Adenocarcinomas”.mp OR “Parotid Adenocarcinoma”.mp OR “Parotid Adenocarcinomas”.mp OR “Pharyngeal Adenocarcinoma”.mp OR “Pharyngeal Adenocarcinomas”.mp OR “Salivary Gland Adenocarcinoma”.mp OR “Salivary Gland Adenocarcinomas”.mp OR “Sublingual Gland Adenocarcinoma”.mp OR “Sublingual Gland Adenocarcinomas”.mp OR “Submandibular Gland Adenocarcinoma”.mp OR “Submandibular Gland Adenocarcinomas”.mp OR “Thyroid Adenocarcinoma”.mp OR “Thyroid Adenocarcinomas”.mp OR “Tongue Adenocarcinoma”.mp OR “Tongue Adenocarcinomas”.mp OR “Tonsillar Adenocarcinoma”.mp OR “Tonsillar Adenocarcinomas”.mp OR “Tracheal Adenocarcinoma”.mp OR “Tracheal Adenocarcinomas”.mp OR “Head and Neck Tumors”.mp OR “Head and Neck Tumor”.mp OR “Ear Tumor”.mp OR “Ear Tumors”.mp OR “Eyelid Tumor”.mp OR “Eyelid Tumors”.mp OR “Facial Tumor”.mp OR “Facial Tumors”.mp OR “Gingival Tumor”.mp OR “Gingival Tumors”.mp OR “Hypopharyngeal Tumor”.mp OR “Hypopharyngeal Tumors”.mp OR “Laryngeal Tumor”.mp OR “Laryngeal Tumors”.mp OR “Lip Tumor”.mp OR “Lip Tumors”.mp OR “Maxillary Sinus Tumor”.mp OR “Maxillary Sinus Tumors”.mp OR “Mouth Tumor”.mp OR “Mouth Tumors”.mp OR “Nasopharyngeal Tumor”.mp OR “Nasopharyngeal Tumors”.mp OR “Nose Tumor”.mp OR “Nose Tumors”.mp OR “Oropharyngeal Tumor”.mp OR “Oropharyngeal Tumors”.mp OR “Otorhinolaryngologic Tumor”.mp OR “Otorhinolaryngologic Tumors”.mp OR “Palatal Tumor”.mp OR “Palatal Tumors”.mp OR “Papillary Thyroid Tumor “.mp OR “Papillary Thyroid Tumors “.mp OR “Paranasal Sinus Tumor”.mp OR “Paranasal Sinus Tumors”.mp OR “Parathyroid Tumor”.mp OR “Parathyroid Tumors”.mp OR “Parotid Tumor”.mp OR “Parotid Tumors”.mp OR “Pharyngeal Tumor”.mp OR “Pharyngeal Tumors”.mp OR “Salivary Gland Tumor”.mp OR “Salivary Gland Tumors”.mp OR “Sublingual Gland Tumor”.mp OR “Sublingual Gland Tumors”.mp OR “Submandibular Gland Tumor”.mp OR “Submandibular Gland Tumors”.mp OR “Thyroid Tumor”.mp OR “Thyroid Tumors”.mp OR “Tongue Tumor”.mp OR “Tongue Tumors”.mp OR “Tonsillar Tumor”.mp OR “Tonsillar Tumors”.mp OR “Tracheal Tumor”.mp OR “Tracheal Tumors”.mp OR “Head and Neck Tumours”.mp OR “Head and Neck Tumour”.mp OR “Ear Tumour”.mp OR “Ear Tumours”.mp OR “Eyelid Tumour”.mp OR “Eyelid Tumours”.mp OR “Facial Tumour”.mp OR “Facial Tumours”.mp OR “Gingival Tumour”.mp OR “Gingival Tumours”.mp OR “Hypopharyngeal Tumour”.mp OR “Hypopharyngeal Tumours”.mp OR “Laryngeal Tumour”.mp OR “Laryngeal Tumours”.mp OR “Lip Tumour”.mp OR “Lip Tumours”.mp OR “Maxillary Sinus Tumour”.mp OR “Maxillary Sinus Tumours”.mp OR “Mouth Tumour”.mp OR “Mouth Tumours”.mp OR “Nasopharyngeal Tumour”.mp OR “Nasopharyngeal Tumours”.mp OR “Nose Tumour”.mp OR “Nose Tumours”.mp OR “Oropharyngeal Tumour”.mp OR “Oropharyngeal Tumours”.mp OR “Otorhinolaryngologic Tumour”.mp OR “Otorhinolaryngologic Tumours”.mp OR “Palatal Tumour”.mp OR “Palatal Tumours”.mp OR “Papillary Thyroid Tumour “.mp OR “Papillary Thyroid Tumours “.mp OR “Paranasal Sinus Tumour”.mp OR “Paranasal Sinus Tumours”.mp OR “Parathyroid Tumour”.mp OR “Parathyroid Tumours”.mp OR “Parotid Tumour”.mp OR “Parotid Tumours”.mp OR “Pharyngeal Tumour”.mp OR “Pharyngeal Tumours”.mp OR “Salivary Gland Tumour”.mp OR “Salivary Gland Tumours”.mp OR “Sublingual Gland Tumour”.mp OR “Sublingual Gland Tumours”.mp OR “Submandibular Gland Tumour”.mp OR “Submandibular Gland Tumours”.mp OR “Thyroid Tumour”.mp OR “Thyroid Tumours”.mp OR “Tongue Tumour”.mp OR “Tongue Tumours”.mp OR “Tonsillar Tumour”.mp OR “Tonsillar Tumours”.mp OR “Tracheal Tumour”.mp OR “Tracheal Tumours”.mp OR “Head and Neck Malignancies”.mp OR “Head and Neck Malignancy”.mp OR “Ear Malignancy”.mp OR “Ear Malignancies”.mp OR “Eyelid Malignancy”.mp OR “Eyelid Malignancies”.mp OR “Facial Malignancy”.mp OR “Facial Malignancies”.mp OR “Gingival Malignancy”.mp OR “Gingival Malignancies”.mp OR “Hypopharyngeal Malignancy”.mp OR “Hypopharyngeal Malignancies”.mp OR “Laryngeal Malignancy”.mp OR “Laryngeal Malignancies”.mp OR “Lip Malignancy”.mp OR “Lip Malignancies”.mp OR “Maxillary Sinus Malignancy”.mp OR “Maxillary Sinus Malignancies”.mp OR “Mouth Malignancy”.mp OR “Mouth Malignancies”.mp OR “Nasopharyngeal Malignancy”.mp OR “Nasopharyngeal Malignancies”.mp OR “Nose Malignancy”.mp OR “Nose Malignancies”.mp OR “Oropharyngeal Malignancy”.mp OR “Oropharyngeal Malignancies”.mp OR “Otorhinolaryngologic Malignancy”.mp OR “Otorhinolaryngologic Malignancies”.mp OR “Palatal Malignancy”.mp OR “Palatal Malignancies”.mp OR “Papillary Thyroid Malignancy “.mp OR “Papillary Thyroid Malignancies “.mp OR “Paranasal Sinus Malignancy”.mp OR “Paranasal Sinus Malignancies”.mp OR “Parathyroid Malignancy”.mp OR “Parathyroid Malignancies”.mp OR “Parotid Malignancy”.mp OR “Parotid Malignancies”.mp OR “Pharyngeal Malignancy”.mp OR “Pharyngeal Malignancies”.mp OR “Salivary Gland Malignancy”.mp OR “Salivary Gland Malignancies”.mp OR “Sublingual Gland Malignancy”.mp OR “Sublingual Gland Malignancies”.mp OR “Submandibular Gland Malignancy”.mp OR “Submandibular Gland Malignancies”.mp OR “Thyroid Malignancy”.mp OR “Thyroid Malignancies”.mp OR “Tongue Malignancy”.mp OR “Tongue Malignancies”.mp OR “Tonsillar Malignancy”.mp OR “Tonsillar Malignancies”.mp OR “Tracheal Malignancy”.mp OR “Tracheal Malignancies”.mp OR “Thyroid Nodule”.mp OR “Oral Leukoplakia”.mp OR “Hairy Leukoplakia”.mp OR exp “Gastrointestinal Neoplasms”/ OR “Gastrointestinal Neoplasms”.mp OR “Gastrointestinal Neoplasm”.mp OR “Stomach Neoplasms”.mp OR “Stomach Neoplasm”.mp OR “Gastric Neoplasms”.mp OR “Gastric Neoplasm”.mp OR “Esophageal Neoplasms”.mp OR “Esophageal Neoplasm”.mp OR “OEsophageal Neoplasms”.mp OR “OEsophageal Neoplasm”.mp OR “Esophagus Neoplasms”.mp OR “Esophagus Neoplasm”.mp OR “OEsophagus Neoplasms”.mp OR “Esophagus Neoplasm”.mp OR “Esophageal Squamus Cell Neoplasms”.mp OR “Esophageal Squamus Cell Neoplasm”.mp OR “OEsophageal Squamus Cell Neoplasms”.mp OR “OEsophageal Squamus Cell Neoplasm”.mp OR “Esophagus Squamus Cell Neoplasms”.mp OR “Esophagus Squamus Cell Neoplasm”.mp OR “OEsophagus Squamus Cell Neoplasms”.mp OR “Esophagus Squamus Cell Neoplasm”.mp OR “Intestinal Neoplasms”.mp OR “Intestinal Neoplasm”.mp OR “Cecal Neoplasms”.mp OR “Cecal Neoplasm”.mp OR “Appenciceal Neoplasms”.mp OR “Appendiceal Neoplasm”.mp OR “Colorectal Neoplasms”.mp OR “Colorectal Neoplasm”.mp OR “Colonic Neoplasms”.mp OR “Colonic Neoplasm”.mp OR “Colon Neoplasms”.mp OR “Colon Neoplasm”.mp OR “Sigmoidal Neoplasms”.mp OR “Sigmoidal Neoplasm”.mp OR “Sigmoid Neoplasms”.mp OR “Sigmoid Neoplasm”.mp OR “Rectal Neoplasms”.mp OR “Rectal Neoplasm”.mp OR “Anorectal Neoplasms”.mp OR “Anorectal Neoplasm”.mp OR “Anus Neoplasms”.mp OR “Anus Neoplasm”.mp OR “Anal Neoplasms”.mp OR “Anal Neoplasm”.mp OR “Anal Gland Neoplasms”.mp OR “Anal Gland Neoplasm”.mp OR “Duodenal Neoplasms”.mp OR “Duodenal Neoplasm”.mp OR “Duodenum Neoplasms”.mp OR “Duodenum Neoplasm”.mp OR “Ileal Neoplasms”.mp OR “Ileal Neoplasm”.mp OR “Ileum Neoplasms”.mp OR “Ileum Neoplasm”.mp OR “Jejunal Neoplasms”.mp OR “Jejunal Neoplasm”.mp OR “Jejunum Neoplasms”.mp OR “Jejunum Neoplasm”.mp OR “Gastrointestinal Cancers”.mp OR “Gastrointestinal Cancer”.mp OR “Stomach Cancers”.mp OR “Stomach Cancer”.mp OR “Gastric Cancers”.mp OR “Gastric Cancer”.mp OR “Esophageal Cancers”.mp OR “Esophageal Cancer”.mp OR “OEsophageal Cancers”.mp OR “OEsophageal Cancer”.mp OR “Esophagus Cancers”.mp OR “Esophagus Cancer”.mp OR “OEsophagus Cancers”.mp OR “Esophagus Cancer”.mp OR “Esophageal Squamus Cell Cancers”.mp OR “Esophageal Squamus Cell Cancer”.mp OR “OEsophageal Squamus Cell Cancers”.mp OR “OEsophageal Squamus Cell Cancer”.mp OR “Esophagus Squamus Cell Cancers”.mp OR “Esophagus Squamus Cell Cancer”.mp OR “OEsophagus Squamus Cell Cancers”.mp OR “Esophagus Squamus Cell Cancer”.mp OR “Intestinal Cancers”.mp OR “Intestinal Cancer”.mp OR “Cecal Cancers”.mp OR “Cecal Cancer”.mp OR “Appenciceal Cancers”.mp OR “Appendiceal Cancer”.mp OR “Colorectal Cancers”.mp OR “Colorectal Cancer”.mp OR “Colonic Cancers”.mp OR “Colonic Cancer”.mp OR “Colon Cancers”.mp OR “Colon Cancer”.mp OR “Sigmoidal Cancers”.mp OR “Sigmoidal Cancer”.mp OR “Sigmoid Cancers”.mp OR “Sigmoid Cancer”.mp OR “Rectal Cancers”.mp OR “Rectal Cancer”.mp OR “Anorectal Cancers”.mp OR “Anorectal Cancer”.mp OR “Anus Cancers”.mp OR “Anus Cancer”.mp OR “Anal Cancers”.mp OR “Anal Cancer”.mp OR “Anal Gland Cancers”.mp OR “Anal Gland Cancer”.mp OR “Duodenal Cancers”.mp OR “Duodenal Cancer”.mp OR “Duodenum Cancers”.mp OR “Duodenum Cancer”.mp OR “Ileal Cancers”.mp OR “Ileal Cancer”.mp OR “Ileum Cancers”.mp OR “Ileum Cancer”.mp OR “Jejunal Cancers”.mp OR “Jejunal Cancer”.mp OR “Jejunum Cancers”.mp OR “Jejunum Cancer”.mp OR “Gastrointestinal Carcinomas”.mp OR “Gastrointestinal Carcinoma”.mp OR “Stomach Carcinomas”.mp OR “Stomach Carcinoma”.mp OR “Gastric Carcinomas”.mp OR “Gastric Carcinoma”.mp OR “Esophageal Carcinomas”.mp OR “Esophageal Carcinoma”.mp OR “OEsophageal Carcinomas”.mp OR “OEsophageal Carcinoma”.mp OR “Esophagus Carcinomas”.mp OR “Esophagus Carcinoma”.mp OR “OEsophagus Carcinomas”.mp OR “Esophagus Carcinoma”.mp OR “Esophageal Squamus Cell Carcinomas”.mp OR “Esophageal Squamus Cell Carcinoma”.mp OR “OEsophageal Squamus Cell Carcinomas”.mp OR “OEsophageal Squamus Cell Carcinoma”.mp OR “Esophagus Squamus Cell Carcinomas”.mp OR “Esophagus Squamus Cell Carcinoma”.mp OR “OEsophagus Squamus Cell Carcinomas”.mp OR “Esophagus Squamus Cell Carcinoma”.mp OR “Intestinal Carcinomas”.mp OR “Intestinal Carcinoma”.mp OR “Cecal Carcinomas”.mp OR “Cecal Carcinoma”.mp OR “Appenciceal Carcinomas”.mp OR “Appendiceal Carcinoma”.mp OR “Colorectal Carcinomas”.mp OR “Colorectal Carcinoma”.mp OR “Colonic Carcinomas”.mp OR “Colonic Carcinoma”.mp OR “Colon Carcinomas”.mp OR “Colon Carcinoma”.mp OR “Sigmoidal Carcinomas”.mp OR “Sigmoidal Carcinoma”.mp OR “Sigmoid Carcinomas”.mp OR “Sigmoid Carcinoma”.mp OR “Rectal Carcinomas”.mp OR “Rectal Carcinoma”.mp OR “Anorectal Carcinomas”.mp OR “Anorectal Carcinoma”.mp OR “Anus Carcinomas”.mp OR “Anus Carcinoma”.mp OR “Anal Carcinomas”.mp OR “Anal Carcinoma”.mp OR “Anal Gland Carcinomas”.mp OR “Anal Gland Carcinoma”.mp OR “Duodenal Carcinomas”.mp OR “Duodenal Carcinoma”.mp OR “Duodenum Carcinomas”.mp OR “Duodenum Carcinoma”.mp OR “Ileal Carcinomas”.mp OR “Ileal Carcinoma”.mp OR “Ileum Carcinomas”.mp OR “Ileum Carcinoma”.mp OR “Jejunal Carcinomas”.mp OR “Jejunal Carcinoma”.mp OR “Jejunum Carcinomas”.mp OR “Jejunum Carcinoma”.mp OR “Gastrointestinal Adenocarcinomas”.mp OR “Gastrointestinal Adenocarcinoma”.mp OR “Stomach Adenocarcinomas”.mp OR “Stomach Adenocarcinoma”.mp OR “Gastric Adenocarcinomas”.mp OR “Gastric Adenocarcinoma”.mp OR “Esophageal Adenocarcinomas”.mp OR “Esophageal Adenocarcinoma”.mp OR “OEsophageal Adenocarcinomas”.mp OR “OEsophageal Adenocarcinoma”.mp OR “Esophagus Adenocarcinomas”.mp OR “Esophagus Adenocarcinoma”.mp OR “OEsophagus Adenocarcinomas”.mp OR “Esophagus Adenocarcinoma”.mp OR “Esophageal Squamus Cell Adenocarcinomas”.mp OR “Esophageal Squamus Cell Adenocarcinoma”.mp OR “OEsophageal Squamus Cell Adenocarcinomas”.mp OR “OEsophageal Squamus Cell Adenocarcinoma”.mp OR “Esophagus Squamus Cell Adenocarcinomas”.mp OR “Esophagus Squamus Cell Adenocarcinoma”.mp OR “OEsophagus Squamus Cell Adenocarcinomas”.mp OR “Esophagus Squamus Cell Adenocarcinoma”.mp OR “Intestinal Adenocarcinomas”.mp OR “Intestinal Adenocarcinoma”.mp OR “Cecal Adenocarcinomas”.mp OR “Cecal Adenocarcinoma”.mp OR “Appenciceal Adenocarcinomas”.mp OR “Appendiceal Adenocarcinoma”.mp OR “Colorectal Adenocarcinomas”.mp OR “Colorectal Adenocarcinoma”.mp OR “Colonic Adenocarcinomas”.mp OR “Colonic Adenocarcinoma”.mp OR “Colon Adenocarcinomas”.mp OR “Colon Adenocarcinoma”.mp OR “Sigmoidal Adenocarcinomas”.mp OR “Sigmoidal Adenocarcinoma”.mp OR “Sigmoid Adenocarcinomas”.mp OR “Sigmoid Adenocarcinoma”.mp OR “Rectal Adenocarcinomas”.mp OR “Rectal Adenocarcinoma”.mp OR “Anorectal Adenocarcinomas”.mp OR “Anorectal Adenocarcinoma”.mp OR “Anus Adenocarcinomas”.mp OR “Anus Adenocarcinoma”.mp OR “Anal Adenocarcinomas”.mp OR “Anal Adenocarcinoma”.mp OR “Anal Gland Adenocarcinomas”.mp OR “Anal Gland Adenocarcinoma”.mp OR “Duodenal Adenocarcinomas”.mp OR “Duodenal Adenocarcinoma”.mp OR “Duodenum Adenocarcinomas”.mp OR “Duodenum Adenocarcinoma”.mp OR “Ileal Adenocarcinomas”.mp OR “Ileal Adenocarcinoma”.mp OR “Ileum Adenocarcinomas”.mp OR “Ileum Adenocarcinoma”.mp OR “Jejunal Adenocarcinomas”.mp OR “Jejunal Adenocarcinoma”.mp OR “Jejunum Adenocarcinomas”.mp OR “Jejunum Adenocarcinoma”.mp OR “Gastrointestinal Tumors”.mp OR “Gastrointestinal Tumor”.mp OR “Stomach Tumors”.mp OR “Stomach Tumor”.mp OR “Gastric Tumors”.mp OR “Gastric Tumor”.mp OR “Esophageal Tumors”.mp OR “Esophageal Tumor”.mp OR “OEsophageal Tumors”.mp OR “OEsophageal Tumor”.mp OR “Esophagus Tumors”.mp OR “Esophagus Tumor”.mp OR “OEsophagus Tumors”.mp OR “Esophagus Tumor”.mp OR “Esophageal Squamus Cell Tumors”.mp OR “Esophageal Squamus Cell Tumor”.mp OR “OEsophageal Squamus Cell Tumors”.mp OR “OEsophageal Squamus Cell Tumor”.mp OR “Esophagus Squamus Cell Tumors”.mp OR “Esophagus Squamus Cell Tumor”.mp OR “OEsophagus Squamus Cell Tumors”.mp OR “Esophagus Squamus Cell Tumor”.mp OR “Intestinal Tumors”.mp OR “Intestinal Tumor”.mp OR “Cecal Tumors”.mp OR “Cecal Tumor”.mp OR “Appenciceal Tumors”.mp OR “Appendiceal Tumor”.mp OR “Colorectal Tumors”.mp OR “Colorectal Tumor”.mp OR “Colonic Tumors”.mp OR “Colonic Tumor”.mp OR “Colon Tumors”.mp OR “Colon Tumor”.mp OR “Sigmoidal Tumors”.mp OR “Sigmoidal Tumor”.mp OR “Sigmoid Tumors”.mp OR “Sigmoid Tumor”.mp OR “Rectal Tumors”.mp OR “Rectal Tumor”.mp OR “Anorectal Tumors”.mp OR “Anorectal Tumor”.mp OR “Anus Tumors”.mp OR “Anus Tumor”.mp OR “Anal Tumors”.mp OR “Anal Tumor”.mp OR “Anal Gland Tumors”.mp OR “Anal Gland Tumor”.mp OR “Duodenal Tumors”.mp OR “Duodenal Tumor”.mp OR “Duodenum Tumors”.mp OR “Duodenum Tumor”.mp OR “Ileal Tumors”.mp OR “Ileal Tumor”.mp OR “Ileum Tumors”.mp OR “Ileum Tumor”.mp OR “Jejunal Tumors”.mp OR “Jejunal Tumor”.mp OR “Jejunum Tumors”.mp OR “Jejunum Tumor”.mp OR “Gastrointestinal Tumours”.mp OR “Gastrointestinal Tumour”.mp OR “Stomach Tumours”.mp OR “Stomach Tumour”.mp OR “Gastric Tumours”.mp OR “Gastric Tumour”.mp OR “Esophageal Tumours”.mp OR “Esophageal Tumour”.mp OR “OEsophageal Tumours”.mp OR “OEsophageal Tumour”.mp OR “Esophagus Tumours”.mp OR “Esophagus Tumour”.mp OR “OEsophagus Tumours”.mp OR “Esophagus Tumour”.mp OR “Esophageal Squamus Cell Tumours”.mp OR “Esophageal Squamus Cell Tumour”.mp OR “OEsophageal Squamus Cell Tumours”.mp OR “OEsophageal Squamus Cell Tumour”.mp OR “Esophagus Squamus Cell Tumours”.mp OR “Esophagus Squamus Cell Tumour”.mp OR “OEsophagus Squamus Cell Tumours”.mp OR “Esophagus Squamus Cell Tumour”.mp OR “Intestinal Tumours”.mp OR “Intestinal Tumour”.mp OR “Cecal Tumours”.mp OR “Cecal Tumour”.mp OR “Appenciceal Tumours”.mp OR “Appendiceal Tumour”.mp OR “Colorectal Tumours”.mp OR “Colorectal Tumour”.mp OR “Colonic Tumours”.mp OR “Colonic Tumour”.mp OR “Colon Tumours”.mp OR “Colon Tumour”.mp OR “Sigmoidal Tumours”.mp OR “Sigmoidal Tumour”.mp OR “Sigmoid Tumours”.mp OR “Sigmoid Tumour”.mp OR “Rectal Tumours”.mp OR “Rectal Tumour”.mp OR “Anorectal Tumours”.mp OR “Anorectal Tumour”.mp OR “Anus Tumours”.mp OR “Anus Tumour”.mp OR “Anal Tumours”.mp OR “Anal Tumour”.mp OR “Anal Gland Tumours”.mp OR “Anal Gland Tumour”.mp OR “Duodenal Tumours”.mp OR “Duodenal Tumour”.mp OR “Duodenum Tumours”.mp OR “Duodenum Tumour”.mp OR “Ileal Tumours”.mp OR “Ileal Tumour”.mp OR “Ileum Tumours”.mp OR “Ileum Tumour”.mp OR “Jejunal Tumours”.mp OR “Jejunal Tumour”.mp OR “Jejunum Tumours”.mp OR “Jejunum Tumour”.mp OR “Gastrointestinal Malignancies”.mp OR “Gastrointestinal Malignancy”.mp OR “Stomach Malignancies”.mp OR “Stomach Malignancy”.mp OR “Gastric Malignancies”.mp OR “Gastric Malignancy”.mp OR “Esophageal Malignancies”.mp OR “Esophageal Malignancy”.mp OR “OEsophageal Malignancies”.mp OR “OEsophageal Malignancy”.mp OR “Esophagus Malignancies”.mp OR “Esophagus Malignancy”.mp OR “OEsophagus Malignancies”.mp OR “Esophagus Malignancy”.mp OR “Esophageal Squamus Cell Malignancies”.mp OR “Esophageal Squamus Cell Malignancy”.mp OR “OEsophageal Squamus Cell Malignancies”.mp OR “OEsophageal Squamus Cell Malignancy”.mp OR “Esophagus Squamus Cell Malignancies”.mp OR “Esophagus Squamus Cell Malignancy”.mp OR “OEsophagus Squamus Cell Malignancies”.mp OR “Esophagus Squamus Cell Malignancy”.mp OR “Intestinal Malignancies”.mp OR “Intestinal Malignancy”.mp OR “Cecal Malignancies”.mp OR “Cecal Malignancy”.mp OR “Appenciceal Malignancies”.mp OR “Appendiceal Malignancy”.mp OR “Colorectal Malignancies”.mp OR “Colorectal Malignancy”.mp OR “Colonic Malignancies”.mp OR “Colonic Malignancy”.mp OR “Colon Malignancies”.mp OR “Colon Malignancy”.mp OR “Sigmoidal Malignancies”.mp OR “Sigmoidal Malignancy”.mp OR “Sigmoid Malignancies”.mp OR “Sigmoid Malignancy”.mp OR “Rectal Malignancies”.mp OR “Rectal Malignancy”.mp OR “Anorectal Malignancies”.mp OR “Anorectal Malignancy”.mp OR “Anus Malignancies”.mp OR “Anus Malignancy”.mp OR “Anal Malignancies”.mp OR “Anal Malignancy”.mp OR “Anal Gland Malignancies”.mp OR “Anal Gland Malignancy”.mp OR “Duodenal Malignancies”.mp OR “Duodenal Malignancy”.mp OR “Duodenum Malignancies”.mp OR “Duodenum Malignancy”.mp OR “Ileal Malignancies”.mp OR “Ileal Malignancy”.mp OR “Ileum Malignancies”.mp OR “Ileum Malignancy”.mp OR “Jejunal Malignancies”.mp OR “Jejunal Malignancy”.mp OR “Jejunum Malignancies”.mp OR “Jejunum Malignancy”.mp OR “Adenomatous Polyposis Coli”.mp OR “Gardner Syndrome”.mp) AND (“resection”.mp OR “resect*”.mp OR “surgery”.fs OR “surgery”.mp OR exp “surgical procedures, operative”/ OR “surgical”.mp OR exp “gastrectomy”/ OR “gastrectomy”.mp OR “gastrectom*”.mp OR exp “Preoperative Period”/ OR exp “Preoperative Care”/ OR “Preoperative Period”.mp OR “Preoperative Care”.mp OR exp “Perioperative Period”/ OR exp “Perioperative Care”/ OR “Perioperative Period”.mp OR “Perioperative Care”.mp OR exp “Postoperative Period”/ OR exp “Postoperative Care”/ OR “Postoperative Period”.mp OR “Postoperative Care”.mp OR “Preoperative”.mp OR “Pre operative”.mp OR “Perioperative”.mp OR “Peri operative”.mp OR “Postoperative”.mp OR “Post operative”.mp) AND (“immunonutrition”.mp OR “immunonutr*”.mp OR “immuno nutrition”.mp OR “immuno nutr*”.mp OR ((exp “Immunomodulation”/ OR “Immunomodulation”.mp OR “Immunomodulat*”.mp OR exp “Immunotherapy”/ OR “immunother*”.mp OR exp “Malnutrition”/im) AND (exp “Nutrition Therapy”/ OR “Nutrition Therapy”.mp OR exp “Diet Therapy”/ OR “Diet Therapy”.mp OR “Caloric Restriction”.mp OR “Carbohydrate Loading diet”.mp OR “Carbohydrate-Restricted diet”.mp OR “High-Protein Low-Carbohydrate diet”.mp OR “Ketogenic diet”.mp OR “Diabetic diet”.mp OR “Fat-Restricted diet”.mp OR “Gluten-Free diet”.mp OR “High-Protein diet”.mp OR “High-Protein Low-Carbohydrate diet”.mp OR “Mediterranean diet”.mp OR “Paleolithic diet”.mp OR “Protein-Restricted diet”.mp OR “Reducing diet”.mp OR “Sodium-Restricted diet”.mp OR “Vegetarian diet”.mp OR “Macrobiotic diet”.mp OR “Vegan diet”.mp OR “Carbohydrate Loading diets”.mp OR “Carbohydrate-Restricted diets”.mp OR “High-Protein Low-Carbohydrate diets”.mp OR “Ketogenic diets”.mp OR “Diabetic diets”.mp OR “Fat-Restricted diets”.mp OR “Gluten-Free diets”.mp OR “High-Protein diets”.mp OR “High-Protein Low-Carbohydrate diets”.mp OR “Mediterranean diets”.mp OR “Paleolithic diets”.mp OR “Protein-Restricted diets”.mp OR “Reducing diets”.mp OR “Sodium-Restricted diets”.mp OR “Vegetarian diets”.mp OR “Macrobiotic diets”.mp OR “Vegan diets”.mp OR “Nutritional Support”.mp OR “Enteral Nutrition”.mp OR “Parenteral Nutrition”.mp OR “Enteral Feeding”.mp OR “Parenteral Feeding”.mp OR “nutritional supplementation”.mp OR “artifical nutrition”.mp OR exp “Diet, Food, and Nutrition”/))) AND (“clinical trial”/ OR “clinical trial”.ti, ab OR exp “clinical trials as topic”/ OR “clinical trials”.ti, ab OR exp “control groups”/ OR “control group”.ti, ab OR “control groups”.ti, ab OR “controlled clinical trial”/ OR exp “controlled clinical trials as topic”/ OR exp “cross-over studies”/ OR “cross over study”.ti, ab OR “cross over studies”.ti, ab OR exp “double-blind method”/ OR “double blind”.ti, ab OR exp “evaluation studies as topic”/ OR exp “follow-up studies”/ OR “follow up study”.ti, ab OR “follow up studies”.ti, ab OR exp “placebos”/ OR placebo*.ti, ab OR placebos*.ti, ab OR exp “pragmatic clinical trial”/ OR exp “prospective studies”/ OR “prospective study”.ti, ab OR “prospective studies”.ti, ab OR “RaCT”.ti, ab OR “RaCTs”.ti, ab OR exp “random allocation”/ OR “randomised”.ti, ab OR “randomized controlled trial”/ OR exp “randomized controlled trials as topic”/ OR “randomized”.ti, ab OR random*.ti, ab OR “RCT”.ti, ab OR “RCTs”.ti, ab OR “Research Design”/ OR “Research design”.ti, ab OR “Research designs”.ti, ab OR “single blind”.ti, ab OR exp “single-blind method”/ OR ((single*.ti, ab OR double*.ti, ab OR triple*.ti, ab) AND (blind*.ti, ab OR mask*.ti, ab)) OR volunteer*.ti, ab OR “trial”.ti OR “trials”.ti))

**EMBASE via OVID**

((exp *”Head and Neck Tumor”/ OR “Head and Neck Neoplasms”.ti, ab OR “Head and Neck Neoplasm”.ti, ab OR “Ear Neoplasm”.ti, ab OR “Ear Neoplasms”.ti, ab OR “Eyelid Neoplasm”.ti, ab OR “Eyelid Neoplasms”.ti, ab OR “Facial Neoplasm”.ti, ab OR “Facial Neoplasms”.ti, ab OR “Gingival Neoplasm”.ti, ab OR “Gingival Neoplasms”.ti, ab OR “Hypopharyngeal Neoplasm”.ti, ab OR “Hypopharyngeal Neoplasms”.ti, ab OR “Laryngeal Neoplasm”.ti, ab OR “Laryngeal Neoplasms”.ti, ab OR “Lip Neoplasm”.ti, ab OR “Lip Neoplasms”.ti, ab OR “Maxillary Sinus Neoplasm”.ti, ab OR “Maxillary Sinus Neoplasms”.ti, ab OR “Mouth Neoplasm”.ti, ab OR “Mouth Neoplasms”.ti, ab OR “Nasopharyngeal Neoplasm”.ti, ab OR “Nasopharyngeal Neoplasms”.ti, ab OR “Nose Neoplasm”.ti, ab OR “Nose Neoplasms”.ti, ab OR “Oropharyngeal Neoplasm”.ti, ab OR “Oropharyngeal Neoplasms”.ti, ab OR “Otorhinolaryngologic Neoplasm”.ti, ab OR “Otorhinolaryngologic Neoplasms”.ti, ab OR “Palatal Neoplasm”.ti, ab OR “Palatal Neoplasms”.ti, ab OR “Papillary Thyroid Neoplasm “.ti, ab OR “Papillary Thyroid Neoplasms “.ti, ab OR “Paranasal Sinus Neoplasm”.ti, ab OR “Paranasal Sinus Neoplasms”.ti, ab OR “Parathyroid Neoplasm”.ti, ab OR “Parathyroid Neoplasms”.ti, ab OR “Parotid Neoplasm”.ti, ab OR “Parotid Neoplasms”.ti, ab OR “Pharyngeal Neoplasm”.ti, ab OR “Pharyngeal Neoplasms”.ti, ab OR “Salivary Gland Neoplasm”.ti, ab OR “Salivary Gland Neoplasms”.ti, ab OR “Sublingual Gland Neoplasm”.ti, ab OR “Sublingual Gland Neoplasms”.ti, ab OR “Submandibular Gland Neoplasm”.ti, ab OR “Submandibular Gland Neoplasms”.ti, ab OR “Thyroid Neoplasm”.ti, ab OR “Thyroid Neoplasms”.ti, ab OR “Tongue Neoplasm”.ti, ab OR “Tongue Neoplasms”.ti, ab OR “Tonsillar Neoplasm”.ti, ab OR “Tonsillar Neoplasms”.ti, ab OR “Tracheal Neoplasm”.ti, ab OR “Tracheal Neoplasms”.ti, ab OR “Head and Neck Cancers”.ti, ab OR “Head and Neck Cancer”.ti, ab OR “Ear Cancer”.ti, ab OR “Ear Cancers”.ti, ab OR “Eyelid Cancer”.ti, ab OR “Eyelid Cancers”.ti, ab OR “Facial Cancer”.ti, ab OR “Facial Cancers”.ti, ab OR “Gingival Cancer”.ti, ab OR “Gingival Cancers”.ti, ab OR “Hypopharyngeal Cancer”.ti, ab OR “Hypopharyngeal Cancers”.ti, ab OR “Laryngeal Cancer”.ti, ab OR “Laryngeal Cancers”.ti, ab OR “Lip Cancer”.ti, ab OR “Lip Cancers”.ti, ab OR “Maxillary Sinus Cancer”.ti, ab OR “Maxillary Sinus Cancers”.ti, ab OR “Mouth Cancer”.ti, ab OR “Mouth Cancers”.ti, ab OR “Nasopharyngeal Cancer”.ti, ab OR “Nasopharyngeal Cancers”.ti, ab OR “Nose Cancer”.ti, ab OR “Nose Cancers”.ti, ab OR “Oropharyngeal Cancer”.ti, ab OR “Oropharyngeal Cancers”.ti, ab OR “Otorhinolaryngologic Cancer”.ti, ab OR “Otorhinolaryngologic Cancers”.ti, ab OR “Palatal Cancer”.ti, ab OR “Palatal Cancers”.ti, ab OR “Papillary Thyroid Cancer “.ti, ab OR “Papillary Thyroid Cancers “.ti, ab OR “Paranasal Sinus Cancer”.ti, ab OR “Paranasal Sinus Cancers”.ti, ab OR “Parathyroid Cancer”.ti, ab OR “Parathyroid Cancers”.ti, ab OR “Parotid Cancer”.ti, ab OR “Parotid Cancers”.ti, ab OR “Pharyngeal Cancer”.ti, ab OR “Pharyngeal Cancers”.ti, ab OR “Salivary Gland Cancer”.ti, ab OR “Salivary Gland Cancers”.ti, ab OR “Sublingual Gland Cancer”.ti, ab OR “Sublingual Gland Cancers”.ti, ab OR “Submandibular Gland Cancer”.ti, ab OR “Submandibular Gland Cancers”.ti, ab OR “Thyroid Cancer”.ti, ab OR “Thyroid Cancers”.ti, ab OR “Tongue Cancer”.ti, ab OR “Tongue Cancers”.ti, ab OR “Tonsillar Cancer”.ti, ab OR “Tonsillar Cancers”.ti, ab OR “Tracheal Cancer”.ti, ab OR “Tracheal Cancers”.ti, ab OR “Head and Neck Carcinomas”.ti, ab OR “Head and Neck Carcinoma”.ti, ab OR “Ear Carcinoma”.ti, ab OR “Ear Carcinomas”.ti, ab OR “Eyelid Carcinoma”.ti, ab OR “Eyelid Carcinomas”.ti, ab OR “Facial Carcinoma”.ti, ab OR “Facial Carcinomas”.ti, ab OR “Gingival Carcinoma”.ti, ab OR “Gingival Carcinomas”.ti, ab OR “Hypopharyngeal Carcinoma”.ti, ab OR “Hypopharyngeal Carcinomas”.ti, ab OR “Laryngeal Carcinoma”.ti, ab OR “Laryngeal Carcinomas”.ti, ab OR “Lip Carcinoma”.ti, ab OR “Lip Carcinomas”.ti, ab OR “Maxillary Sinus Carcinoma”.ti, ab OR “Maxillary Sinus Carcinomas”.ti, ab OR “Mouth Carcinoma”.ti, ab OR “Mouth Carcinomas”.ti, ab OR “Nasopharyngeal Carcinoma”.ti, ab OR “Nasopharyngeal Carcinomas”.ti, ab OR “Nose Carcinoma”.ti, ab OR “Nose Carcinomas”.ti, ab OR “Oropharyngeal Carcinoma”.ti, ab OR “Oropharyngeal Carcinomas”.ti, ab OR “Otorhinolaryngologic Carcinoma”.ti, ab OR “Otorhinolaryngologic Carcinomas”.ti, ab OR “Palatal Carcinoma”.ti, ab OR “Palatal Carcinomas”.ti, ab OR “Papillary Thyroid Carcinoma “.ti, ab OR “Papillary Thyroid Carcinomas “.ti, ab OR “Paranasal Sinus Carcinoma”.ti, ab OR “Paranasal Sinus Carcinomas”.ti, ab OR “Parathyroid Carcinoma”.ti, ab OR “Parathyroid Carcinomas”.ti, ab OR “Parotid Carcinoma”.ti, ab OR “Parotid Carcinomas”.ti, ab OR “Pharyngeal Carcinoma”.ti, ab OR “Pharyngeal Carcinomas”.ti, ab OR “Salivary Gland Carcinoma”.ti, ab OR “Salivary Gland Carcinomas”.ti, ab OR “Sublingual Gland Carcinoma”.ti, ab OR “Sublingual Gland Carcinomas”.ti, ab OR “Submandibular Gland Carcinoma”.ti, ab OR “Submandibular Gland Carcinomas”.ti, ab OR “Thyroid Carcinoma”.ti, ab OR “Thyroid Carcinomas”.ti, ab OR “Tongue Carcinoma”.ti, ab OR “Tongue Carcinomas”.ti, ab OR “Tonsillar Carcinoma”.ti, ab OR “Tonsillar Carcinomas”.ti, ab OR “Tracheal Carcinoma”.ti, ab OR “Tracheal Carcinomas”.ti, ab OR “Head and Neck Adenocarcinomas”.ti, ab OR “Head and Neck Adenocarcinoma”.ti, ab OR “Ear Adenocarcinoma”.ti, ab OR “Ear Adenocarcinomas”.ti, ab OR “Eyelid Adenocarcinoma”.ti, ab OR “Eyelid Adenocarcinomas”.ti, ab OR “Facial Adenocarcinoma”.ti, ab OR “Facial Adenocarcinomas”.ti, ab OR “Gingival Adenocarcinoma”.ti, ab OR “Gingival Adenocarcinomas”.ti, ab OR “Hypopharyngeal Adenocarcinoma”.ti, ab OR “Hypopharyngeal Adenocarcinomas”.ti, ab OR “Laryngeal Adenocarcinoma”.ti, ab OR “Laryngeal Adenocarcinomas”.ti, ab OR “Lip Adenocarcinoma”.ti, ab OR “Lip Adenocarcinomas”.ti, ab OR “Maxillary Sinus Adenocarcinoma”.ti, ab OR “Maxillary Sinus Adenocarcinomas”.ti, ab OR “Mouth Adenocarcinoma”.ti, ab OR “Mouth Adenocarcinomas”.ti, ab OR “Nasopharyngeal Adenocarcinoma”.ti, ab OR “Nasopharyngeal Adenocarcinomas”.ti, ab OR “Nose Adenocarcinoma”.ti, ab OR “Nose Adenocarcinomas”.ti, ab OR “Oropharyngeal Adenocarcinoma”.ti, ab OR “Oropharyngeal Adenocarcinomas”.ti, ab OR “Otorhinolaryngologic Adenocarcinoma”.ti, ab OR “Otorhinolaryngologic Adenocarcinomas”.ti, ab OR “Palatal Adenocarcinoma”.ti, ab OR “Palatal Adenocarcinomas”.ti, ab OR “Papillary Thyroid Adenocarcinoma “.ti, ab OR “Papillary Thyroid Adenocarcinomas “.ti, ab OR “Paranasal Sinus Adenocarcinoma”.ti, ab OR “Paranasal Sinus Adenocarcinomas”.ti, ab OR “Parathyroid Adenocarcinoma”.ti, ab OR “Parathyroid Adenocarcinomas”.ti, ab OR “Parotid Adenocarcinoma”.ti, ab OR “Parotid Adenocarcinomas”.ti, ab OR “Pharyngeal Adenocarcinoma”.ti, ab OR “Pharyngeal Adenocarcinomas”.ti, ab OR “Salivary Gland Adenocarcinoma”.ti, ab OR “Salivary Gland Adenocarcinomas”.ti, ab OR “Sublingual Gland Adenocarcinoma”.ti, ab OR “Sublingual Gland Adenocarcinomas”.ti, ab OR “Submandibular Gland Adenocarcinoma”.ti, ab OR “Submandibular Gland Adenocarcinomas”.ti, ab OR “Thyroid Adenocarcinoma”.ti, ab OR “Thyroid Adenocarcinomas”.ti, ab OR “Tongue Adenocarcinoma”.ti, ab OR “Tongue Adenocarcinomas”.ti, ab OR “Tonsillar Adenocarcinoma”.ti, ab OR “Tonsillar Adenocarcinomas”.ti, ab OR “Tracheal Adenocarcinoma”.ti, ab OR “Tracheal Adenocarcinomas”.ti, ab OR “Head and Neck Tumors”.ti, ab OR “Head and Neck Tumor”.ti, ab OR “Ear Tumor”.ti, ab OR “Ear Tumors”.ti, ab OR “Eyelid Tumor”.ti, ab OR “Eyelid Tumors”.ti, ab OR “Facial Tumor”.ti, ab OR “Facial Tumors”.ti, ab OR “Gingival Tumor”.ti, ab OR “Gingival Tumors”.ti, ab OR “Hypopharyngeal Tumor”.ti, ab OR “Hypopharyngeal Tumors”.ti, ab OR “Laryngeal Tumor”.ti, ab OR “Laryngeal Tumors”.ti, ab OR “Lip Tumor”.ti, ab OR “Lip Tumors”.ti, ab OR “Maxillary Sinus Tumor”.ti, ab OR “Maxillary Sinus Tumors”.ti, ab OR “Mouth Tumor”.ti, ab OR “Mouth Tumors”.ti, ab OR “Nasopharyngeal Tumor”.ti, ab OR “Nasopharyngeal Tumors”.ti, ab OR “Nose Tumor”.ti, ab OR “Nose Tumors”.ti, ab OR “Oropharyngeal Tumor”.ti, ab OR “Oropharyngeal Tumors”.ti, ab OR “Otorhinolaryngologic Tumor”.ti, ab OR “Otorhinolaryngologic Tumors”.ti, ab OR “Palatal Tumor”.ti, ab OR “Palatal Tumors”.ti, ab OR “Papillary Thyroid Tumor “.ti, ab OR “Papillary Thyroid Tumors “.ti, ab OR “Paranasal Sinus Tumor”.ti, ab OR “Paranasal Sinus Tumors”.ti, ab OR “Parathyroid Tumor”.ti, ab OR “Parathyroid Tumors”.ti, ab OR “Parotid Tumor”.ti, ab OR “Parotid Tumors”.ti, ab OR “Pharyngeal Tumor”.ti, ab OR “Pharyngeal Tumors”.ti, ab OR “Salivary Gland Tumor”.ti, ab OR “Salivary Gland Tumors”.ti, ab OR “Sublingual Gland Tumor”.ti, ab OR “Sublingual Gland Tumors”.ti, ab OR “Submandibular Gland Tumor”.ti, ab OR “Submandibular Gland Tumors”.ti, ab OR “Thyroid Tumor”.ti, ab OR “Thyroid Tumors”.ti, ab OR “Tongue Tumor”.ti, ab OR “Tongue Tumors”.ti, ab OR “Tonsillar Tumor”.ti, ab OR “Tonsillar Tumors”.ti, ab OR “Tracheal Tumor”.ti, ab OR “Tracheal Tumors”.ti, ab OR “Head and Neck Tumours”.ti, ab OR “Head and Neck Tumour”.ti, ab OR “Ear Tumour”.ti, ab OR “Ear Tumours”.ti, ab OR “Eyelid Tumour”.ti, ab OR “Eyelid Tumours”.ti, ab OR “Facial Tumour”.ti, ab OR “Facial Tumours”.ti, ab OR “Gingival Tumour”.ti, ab OR “Gingival Tumours”.ti, ab OR “Hypopharyngeal Tumour”.ti, ab OR “Hypopharyngeal Tumours”.ti, ab OR “Laryngeal Tumour”.ti, ab OR “Laryngeal Tumours”.ti, ab OR “Lip Tumour”.ti, ab OR “Lip Tumours”.ti, ab OR “Maxillary Sinus Tumour”.ti, ab OR “Maxillary Sinus Tumours”.ti, ab OR “Mouth Tumour”.ti, ab OR “Mouth Tumours”.ti, ab OR “Nasopharyngeal Tumour”.ti, ab OR “Nasopharyngeal Tumours”.ti, ab OR “Nose Tumour”.ti, ab OR “Nose Tumours”.ti, ab OR “Oropharyngeal Tumour”.ti, ab OR “Oropharyngeal Tumours”.ti, ab OR “Otorhinolaryngologic Tumour”.ti, ab OR “Otorhinolaryngologic Tumours”.ti, ab OR “Palatal Tumour”.ti, ab OR “Palatal Tumours”.ti, ab OR “Papillary Thyroid Tumour “.ti, ab OR “Papillary Thyroid Tumours “.ti, ab OR “Paranasal Sinus Tumour”.ti, ab OR “Paranasal Sinus Tumours”.ti, ab OR “Parathyroid Tumour”.ti, ab OR “Parathyroid Tumours”.ti, ab OR “Parotid Tumour”.ti, ab OR “Parotid Tumours”.ti, ab OR “Pharyngeal Tumour”.ti, ab OR “Pharyngeal Tumours”.ti, ab OR “Salivary Gland Tumour”.ti, ab OR “Salivary Gland Tumours”.ti, ab OR “Sublingual Gland Tumour”.ti, ab OR “Sublingual Gland Tumours”.ti, ab OR “Submandibular Gland Tumour”.ti, ab OR “Submandibular Gland Tumours”.ti, ab OR “Thyroid Tumour”.ti, ab OR “Thyroid Tumours”.ti, ab OR “Tongue Tumour”.ti, ab OR “Tongue Tumours”.ti, ab OR “Tonsillar Tumour”.ti, ab OR “Tonsillar Tumours”.ti, ab OR “Tracheal Tumour”.ti, ab OR “Tracheal Tumours”.ti, ab OR “Head and Neck Malignancies”.ti, ab OR “Head and Neck Malignancy”.ti, ab OR “Ear Malignancy”.ti, ab OR “Ear Malignancies”.ti, ab OR “Eyelid Malignancy”.ti, ab OR “Eyelid Malignancies”.ti, ab OR “Facial Malignancy”.ti, ab OR “Facial Malignancies”.ti, ab OR “Gingival Malignancy”.ti, ab OR “Gingival Malignancies”.ti, ab OR “Hypopharyngeal Malignancy”.ti, ab OR “Hypopharyngeal Malignancies”.ti, ab OR “Laryngeal Malignancy”.ti, ab OR “Laryngeal Malignancies”.ti, ab OR “Lip Malignancy”.ti, ab OR “Lip Malignancies”.ti, ab OR “Maxillary Sinus Malignancy”.ti, ab OR “Maxillary Sinus Malignancies”.ti, ab OR “Mouth Malignancy”.ti, ab OR “Mouth Malignancies”.ti, ab OR “Nasopharyngeal Malignancy”.ti, ab OR “Nasopharyngeal Malignancies”.ti, ab OR “Nose Malignancy”.ti, ab OR “Nose Malignancies”.ti, ab OR “Oropharyngeal Malignancy”.ti, ab OR “Oropharyngeal Malignancies”.ti, ab OR “Otorhinolaryngologic Malignancy”.ti, ab OR “Otorhinolaryngologic Malignancies”.ti, ab OR “Palatal Malignancy”.ti, ab OR “Palatal Malignancies”.ti, ab OR “Papillary Thyroid Malignancy “.ti, ab OR “Papillary Thyroid Malignancies “.ti, ab OR “Paranasal Sinus Malignancy”.ti, ab OR “Paranasal Sinus Malignancies”.ti, ab OR “Parathyroid Malignancy”.ti, ab OR “Parathyroid Malignancies”.ti, ab OR “Parotid Malignancy”.ti, ab OR “Parotid Malignancies”.ti, ab OR “Pharyngeal Malignancy”.ti, ab OR “Pharyngeal Malignancies”.ti, ab OR “Salivary Gland Malignancy”.ti, ab OR “Salivary Gland Malignancies”.ti, ab OR “Sublingual Gland Malignancy”.ti, ab OR “Sublingual Gland Malignancies”.ti, ab OR “Submandibular Gland Malignancy”.ti, ab OR “Submandibular Gland Malignancies”.ti, ab OR “Thyroid Malignancy”.ti, ab OR “Thyroid Malignancies”.ti, ab OR “Tongue Malignancy”.ti, ab OR “Tongue Malignancies”.ti, ab OR “Tonsillar Malignancy”.ti, ab OR “Tonsillar Malignancies”.ti, ab OR “Tracheal Malignancy”.ti, ab OR “Tracheal Malignancies”.ti, ab OR “Thyroid Nodule”.ti, ab OR “Oral Leukoplakia”.ti, ab OR “Hairy Leukoplakia”.ti, ab OR exp *”Digestive System Tumor”/ OR “Gastrointestinal Neoplasms”.ti, ab OR “Gastrointestinal Neoplasm”.ti, ab OR “Stomach Neoplasms”.ti, ab OR “Stomach Neoplasm”.ti, ab OR “Gastric Neoplasms”.ti, ab OR “Gastric Neoplasm”.ti, ab OR “Esophageal Neoplasms”.ti, ab OR “Esophageal Neoplasm”.ti, ab OR “OEsophageal Neoplasms”.ti, ab OR “OEsophageal Neoplasm”.ti, ab OR “Esophagus Neoplasms”.ti, ab OR “Esophagus Neoplasm”.ti, ab OR “OEsophagus Neoplasms”.ti, ab OR “Esophagus Neoplasm”.ti, ab OR “Esophageal Squamus Cell Neoplasms”.ti, ab OR “Esophageal Squamus Cell Neoplasm”.ti, ab OR “OEsophageal Squamus Cell Neoplasms”.ti, ab OR “OEsophageal Squamus Cell Neoplasm”.ti, ab OR “Esophagus Squamus Cell Neoplasms”.ti, ab OR “Esophagus Squamus Cell Neoplasm”.ti, ab OR “OEsophagus Squamus Cell Neoplasms”.ti, ab OR “Esophagus Squamus Cell Neoplasm”.ti, ab OR “Intestinal Neoplasms”.ti, ab OR “Intestinal Neoplasm”.ti, ab OR “Cecal Neoplasms”.ti, ab OR “Cecal Neoplasm”.ti, ab OR “Appenciceal Neoplasms”.ti, ab OR “Appendiceal Neoplasm”.ti, ab OR “Colorectal Neoplasms”.ti, ab OR “Colorectal Neoplasm”.ti, ab OR “Colonic Neoplasms”.ti, ab OR “Colonic Neoplasm”.ti, ab OR “Colon Neoplasms”.ti, ab OR “Colon Neoplasm”.ti, ab OR “Sigmoidal Neoplasms”.ti, ab OR “Sigmoidal Neoplasm”.ti, ab OR “Sigmoid Neoplasms”.ti, ab OR “Sigmoid Neoplasm”.ti, ab OR “Rectal Neoplasms”.ti, ab OR “Rectal Neoplasm”.ti, ab OR “Anorectal Neoplasms”.ti, ab OR “Anorectal Neoplasm”.ti, ab OR “Anus Neoplasms”.ti, ab OR “Anus Neoplasm”.ti, ab OR “Anal Neoplasms”.ti, ab OR “Anal Neoplasm”.ti, ab OR “Anal Gland Neoplasms”.ti, ab OR “Anal Gland Neoplasm”.ti, ab OR “Duodenal Neoplasms”.ti, ab OR “Duodenal Neoplasm”.ti, ab OR “Duodenum Neoplasms”.ti, ab OR “Duodenum Neoplasm”.ti, ab OR “Ileal Neoplasms”.ti, ab OR “Ileal Neoplasm”.ti, ab OR “Ileum Neoplasms”.ti, ab OR “Ileum Neoplasm”.ti, ab OR “Jejunal Neoplasms”.ti, ab OR “Jejunal Neoplasm”.ti, ab OR “Jejunum Neoplasms”.ti, ab OR “Jejunum Neoplasm”.ti, ab OR “Gastrointestinal Cancers”.ti, ab OR “Gastrointestinal Cancer”.ti, ab OR “Stomach Cancers”.ti, ab OR “Stomach Cancer”.ti, ab OR “Gastric Cancers”.ti, ab OR “Gastric Cancer”.ti, ab OR “Esophageal Cancers”.ti, ab OR “Esophageal Cancer”.ti, ab OR “OEsophageal Cancers”.ti, ab OR “OEsophageal Cancer”.ti, ab OR “Esophagus Cancers”.ti, ab OR “Esophagus Cancer”.ti, ab OR “OEsophagus Cancers”.ti, ab OR “Esophagus Cancer”.ti, ab OR “Esophageal Squamus Cell Cancers”.ti, ab OR “Esophageal Squamus Cell Cancer”.ti, ab OR “OEsophageal Squamus Cell Cancers”.ti, ab OR “OEsophageal Squamus Cell Cancer”.ti, ab OR “Esophagus Squamus Cell Cancers”.ti, ab OR “Esophagus Squamus Cell Cancer”.ti, ab OR “OEsophagus Squamus Cell Cancers”.ti, ab OR “Esophagus Squamus Cell Cancer”.ti, ab OR “Intestinal Cancers”.ti, ab OR “Intestinal Cancer”.ti, ab OR “Cecal Cancers”.ti, ab OR “Cecal Cancer”.ti, ab OR “Appenciceal Cancers”.ti, ab OR “Appendiceal Cancer”.ti, ab OR “Colorectal Cancers”.ti, ab OR “Colorectal Cancer”.ti, ab OR “Colonic Cancers”.ti, ab OR “Colonic Cancer”.ti, ab OR “Colon Cancers”.ti, ab OR “Colon Cancer”.ti, ab OR “Sigmoidal Cancers”.ti, ab OR “Sigmoidal Cancer”.ti, ab OR “Sigmoid Cancers”.ti, ab OR “Sigmoid Cancer”.ti, ab OR “Rectal Cancers”.ti, ab OR “Rectal Cancer”.ti, ab OR “Anorectal Cancers”.ti, ab OR “Anorectal Cancer”.ti, ab OR “Anus Cancers”.ti, ab OR “Anus Cancer”.ti, ab OR “Anal Cancers”.ti, ab OR “Anal Cancer”.ti, ab OR “Anal Gland Cancers”.ti, ab OR “Anal Gland Cancer”.ti, ab OR “Duodenal Cancers”.ti, ab OR “Duodenal Cancer”.ti, ab OR “Duodenum Cancers”.ti, ab OR “Duodenum Cancer”.ti, ab OR “Ileal Cancers”.ti, ab OR “Ileal Cancer”.ti, ab OR “Ileum Cancers”.ti, ab OR “Ileum Cancer”.ti, ab OR “Jejunal Cancers”.ti, ab OR “Jejunal Cancer”.ti, ab OR “Jejunum Cancers”.ti, ab OR “Jejunum Cancer”.ti, ab OR “Gastrointestinal Carcinomas”.ti, ab OR “Gastrointestinal Carcinoma”.ti, ab OR “Stomach Carcinomas”.ti, ab OR “Stomach Carcinoma”.ti, ab OR “Gastric Carcinomas”.ti, ab OR “Gastric Carcinoma”.ti, ab OR “Esophageal Carcinomas”.ti, ab OR “Esophageal Carcinoma”.ti, ab OR “OEsophageal Carcinomas”.ti, ab OR “OEsophageal Carcinoma”.ti, ab OR “Esophagus Carcinomas”.ti, ab OR “Esophagus Carcinoma”.ti, ab OR “OEsophagus Carcinomas”.ti, ab OR “Esophagus Carcinoma”.ti, ab OR “Esophageal Squamus Cell Carcinomas”.ti, ab OR “Esophageal Squamus Cell Carcinoma”.ti, ab OR “OEsophageal Squamus Cell Carcinomas”.ti, ab OR “OEsophageal Squamus Cell Carcinoma”.ti, ab OR “Esophagus Squamus Cell Carcinomas”.ti, ab OR “Esophagus Squamus Cell Carcinoma”.ti, ab OR “OEsophagus Squamus Cell Carcinomas”.ti, ab OR “Esophagus Squamus Cell Carcinoma”.ti, ab OR “Intestinal Carcinomas”.ti, ab OR “Intestinal Carcinoma”.ti, ab OR “Cecal Carcinomas”.ti, ab OR “Cecal Carcinoma”.ti, ab OR “Appenciceal Carcinomas”.ti, ab OR “Appendiceal Carcinoma”.ti, ab OR “Colorectal Carcinomas”.ti, ab OR “Colorectal Carcinoma”.ti, ab OR “Colonic Carcinomas”.ti, ab OR “Colonic Carcinoma”.ti, ab OR “Colon Carcinomas”.ti, ab OR “Colon Carcinoma”.ti, ab OR “Sigmoidal Carcinomas”.ti, ab OR “Sigmoidal Carcinoma”.ti, ab OR “Sigmoid Carcinomas”.ti, ab OR “Sigmoid Carcinoma”.ti, ab OR “Rectal Carcinomas”.ti, ab OR “Rectal Carcinoma”.ti, ab OR “Anorectal Carcinomas”.ti, ab OR “Anorectal Carcinoma”.ti, ab OR “Anus Carcinomas”.ti, ab OR “Anus Carcinoma”.ti, ab OR “Anal Carcinomas”.ti, ab OR “Anal Carcinoma”.ti, ab OR “Anal Gland Carcinomas”.ti, ab OR “Anal Gland Carcinoma”.ti, ab OR “Duodenal Carcinomas”.ti, ab OR “Duodenal Carcinoma”.ti, ab OR “Duodenum Carcinomas”.ti, ab OR “Duodenum Carcinoma”.ti, ab OR “Ileal Carcinomas”.ti, ab OR “Ileal Carcinoma”.ti, ab OR “Ileum Carcinomas”.ti, ab OR “Ileum Carcinoma”.ti, ab OR “Jejunal Carcinomas”.ti, ab OR “Jejunal Carcinoma”.ti, ab OR “Jejunum Carcinomas”.ti, ab OR “Jejunum Carcinoma”.ti, ab OR “Gastrointestinal Adenocarcinomas”.ti, ab OR “Gastrointestinal Adenocarcinoma”.ti, ab OR “Stomach Adenocarcinomas”.ti, ab OR “Stomach Adenocarcinoma”.ti, ab OR “Gastric Adenocarcinomas”.ti, ab OR “Gastric Adenocarcinoma”.ti, ab OR “Esophageal Adenocarcinomas”.ti, ab OR “Esophageal Adenocarcinoma”.ti, ab OR “OEsophageal Adenocarcinomas”.ti, ab OR “OEsophageal Adenocarcinoma”.ti, ab OR “Esophagus Adenocarcinomas”.ti, ab OR “Esophagus Adenocarcinoma”.ti, ab OR “OEsophagus Adenocarcinomas”.ti, ab OR “Esophagus Adenocarcinoma”.ti, ab OR “Esophageal Squamus Cell Adenocarcinomas”.ti, ab OR “Esophageal Squamus Cell Adenocarcinoma”.ti, ab OR “OEsophageal Squamus Cell Adenocarcinomas”.ti, ab OR “OEsophageal Squamus Cell Adenocarcinoma”.ti, ab OR “Esophagus Squamus Cell Adenocarcinomas”.ti, ab OR “Esophagus Squamus Cell Adenocarcinoma”.ti, ab OR “OEsophagus Squamus Cell Adenocarcinomas”.ti, ab OR “Esophagus Squamus Cell Adenocarcinoma”.ti, ab OR “Intestinal Adenocarcinomas”.ti, ab OR “Intestinal Adenocarcinoma”.ti, ab OR “Cecal Adenocarcinomas”.ti, ab OR “Cecal Adenocarcinoma”.ti, ab OR “Appenciceal Adenocarcinomas”.ti, ab OR “Appendiceal Adenocarcinoma”.ti, ab OR “Colorectal Adenocarcinomas”.ti, ab OR “Colorectal Adenocarcinoma”.ti, ab OR “Colonic Adenocarcinomas”.ti, ab OR “Colonic Adenocarcinoma”.ti, ab OR “Colon Adenocarcinomas”.ti, ab OR “Colon Adenocarcinoma”.ti, ab OR “Sigmoidal Adenocarcinomas”.ti, ab OR “Sigmoidal Adenocarcinoma”.ti, ab OR “Sigmoid Adenocarcinomas”.ti, ab OR “Sigmoid Adenocarcinoma”.ti, ab OR “Rectal Adenocarcinomas”.ti, ab OR “Rectal Adenocarcinoma”.ti, ab OR “Anorectal Adenocarcinomas”.ti, ab OR “Anorectal Adenocarcinoma”.ti, ab OR “Anus Adenocarcinomas”.ti, ab OR “Anus Adenocarcinoma”.ti, ab OR “Anal Adenocarcinomas”.ti, ab OR “Anal Adenocarcinoma”.ti, ab OR “Anal Gland Adenocarcinomas”.ti, ab OR “Anal Gland Adenocarcinoma”.ti, ab OR “Duodenal Adenocarcinomas”.ti, ab OR “Duodenal Adenocarcinoma”.ti, ab OR “Duodenum Adenocarcinomas”.ti, ab OR “Duodenum Adenocarcinoma”.ti, ab OR “Ileal Adenocarcinomas”.ti, ab OR “Ileal Adenocarcinoma”.ti, ab OR “Ileum Adenocarcinomas”.ti, ab OR “Ileum Adenocarcinoma”.ti, ab OR “Jejunal Adenocarcinomas”.ti, ab OR “Jejunal Adenocarcinoma”.ti, ab OR “Jejunum Adenocarcinomas”.ti, ab OR “Jejunum Adenocarcinoma”.ti, ab OR “Gastrointestinal Tumors”.ti, ab OR “Gastrointestinal Tumor”.ti, ab OR “Stomach Tumors”.ti, ab OR “Stomach Tumor”.ti, ab OR “Gastric Tumors”.ti, ab OR “Gastric Tumor”.ti, ab OR “Esophageal Tumors”.ti, ab OR “Esophageal Tumor”.ti, ab OR “OEsophageal Tumors”.ti, ab OR “OEsophageal Tumor”.ti, ab OR “Esophagus Tumors”.ti, ab OR “Esophagus Tumor”.ti, ab OR “OEsophagus Tumors”.ti, ab OR “Esophagus Tumor”.ti, ab OR “Esophageal Squamus Cell Tumors”.ti, ab OR “Esophageal Squamus Cell Tumor”.ti, ab OR “OEsophageal Squamus Cell Tumors”.ti, ab OR “OEsophageal Squamus Cell Tumor”.ti, ab OR “Esophagus Squamus Cell Tumors”.ti, ab OR “Esophagus Squamus Cell Tumor”.ti, ab OR “OEsophagus Squamus Cell Tumors”.ti, ab OR “Esophagus Squamus Cell Tumor”.ti, ab OR “Intestinal Tumors”.ti, ab OR “Intestinal Tumor”.ti, ab OR “Cecal Tumors”.ti, ab OR “Cecal Tumor”.ti, ab OR “Appenciceal Tumors”.ti, ab OR “Appendiceal Tumor”.ti, ab OR “Colorectal Tumors”.ti, ab OR “Colorectal Tumor”.ti, ab OR “Colonic Tumors”.ti, ab OR “Colonic Tumor”.ti, ab OR “Colon Tumors”.ti, ab OR “Colon Tumor”.ti, ab OR “Sigmoidal Tumors”.ti, ab OR “Sigmoidal Tumor”.ti, ab OR “Sigmoid Tumors”.ti, ab OR “Sigmoid Tumor”.ti, ab OR “Rectal Tumors”.ti, ab OR “Rectal Tumor”.ti, ab OR “Anorectal Tumors”.ti, ab OR “Anorectal Tumor”.ti, ab OR “Anus Tumors”.ti, ab OR “Anus Tumor”.ti, ab OR “Anal Tumors”.ti, ab OR “Anal Tumor”.ti, ab OR “Anal Gland Tumors”.ti, ab OR “Anal Gland Tumor”.ti, ab OR “Duodenal Tumors”.ti, ab OR “Duodenal Tumor”.ti, ab OR “Duodenum Tumors”.ti, ab OR “Duodenum Tumor”.ti, ab OR “Ileal Tumors”.ti, ab OR “Ileal Tumor”.ti, ab OR “Ileum Tumors”.ti, ab OR “Ileum Tumor”.ti, ab OR “Jejunal Tumors”.ti, ab OR “Jejunal Tumor”.ti, ab OR “Jejunum Tumors”.ti, ab OR “Jejunum Tumor”.ti, ab OR “Gastrointestinal Tumours”.ti, ab OR “Gastrointestinal Tumour”.ti, ab OR “Stomach Tumours”.ti, ab OR “Stomach Tumour”.ti, ab OR “Gastric Tumours”.ti, ab OR “Gastric Tumour”.ti, ab OR “Esophageal Tumours”.ti, ab OR “Esophageal Tumour”.ti, ab OR “OEsophageal Tumours”.ti, ab OR “OEsophageal Tumour”.ti, ab OR “Esophagus Tumours”.ti, ab OR “Esophagus Tumour”.ti, ab OR “OEsophagus Tumours”.ti, ab OR “Esophagus Tumour”.ti, ab OR “Esophageal Squamus Cell Tumours”.ti, ab OR “Esophageal Squamus Cell Tumour”.ti, ab OR “OEsophageal Squamus Cell Tumours”.ti, ab OR “OEsophageal Squamus Cell Tumour”.ti, ab OR “Esophagus Squamus Cell Tumours”.ti, ab OR “Esophagus Squamus Cell Tumour”.ti, ab OR “OEsophagus Squamus Cell Tumours”.ti, ab OR “Esophagus Squamus Cell Tumour”.ti, ab OR “Intestinal Tumours”.ti, ab OR “Intestinal Tumour”.ti, ab OR “Cecal Tumours”.ti, ab OR “Cecal Tumour”.ti, ab OR “Appenciceal Tumours”.ti, ab OR “Appendiceal Tumour”.ti, ab OR “Colorectal Tumours”.ti, ab OR “Colorectal Tumour”.ti, ab OR “Colonic Tumours”.ti, ab OR “Colonic Tumour”.ti, ab OR “Colon Tumours”.ti, ab OR “Colon Tumour”.ti, ab OR “Sigmoidal Tumours”.ti, ab OR “Sigmoidal Tumour”.ti, ab OR “Sigmoid Tumours”.ti, ab OR “Sigmoid Tumour”.ti, ab OR “Rectal Tumours”.ti, ab OR “Rectal Tumour”.ti, ab OR “Anorectal Tumours”.ti, ab OR “Anorectal Tumour”.ti, ab OR “Anus Tumours”.ti, ab OR “Anus Tumour”.ti, ab OR “Anal Tumours”.ti, ab OR “Anal Tumour”.ti, ab OR “Anal Gland Tumours”.ti, ab OR “Anal Gland Tumour”.ti, ab OR “Duodenal Tumours”.ti, ab OR “Duodenal Tumour”.ti, ab OR “Duodenum Tumours”.ti, ab OR “Duodenum Tumour”.ti, ab OR “Ileal Tumours”.ti, ab OR “Ileal Tumour”.ti, ab OR “Ileum Tumours”.ti, ab OR “Ileum Tumour”.ti, ab OR “Jejunal Tumours”.ti, ab OR “Jejunal Tumour”.ti, ab OR “Jejunum Tumours”.ti, ab OR “Jejunum Tumour”.ti, ab OR “Gastrointestinal Malignancies”.ti, ab OR “Gastrointestinal Malignancy”.ti, ab OR “Stomach Malignancies”.ti, ab OR “Stomach Malignancy”.ti, ab OR “Gastric Malignancies”.ti, ab OR “Gastric Malignancy”.ti, ab OR “Esophageal Malignancies”.ti, ab OR “Esophageal Malignancy”.ti, ab OR “OEsophageal Malignancies”.ti, ab OR “OEsophageal Malignancy”.ti, ab OR “Esophagus Malignancies”.ti, ab OR “Esophagus Malignancy”.ti, ab OR “OEsophagus Malignancies”.ti, ab OR “Esophagus Malignancy”.ti, ab OR “Esophageal Squamus Cell Malignancies”.ti, ab OR “Esophageal Squamus Cell Malignancy”.ti, ab OR “OEsophageal Squamus Cell Malignancies”.ti, ab OR “OEsophageal Squamus Cell Malignancy”.ti, ab OR “Esophagus Squamus Cell Malignancies”.ti, ab OR “Esophagus Squamus Cell Malignancy”.ti, ab OR “OEsophagus Squamus Cell Malignancies”.ti, ab OR “Esophagus Squamus Cell Malignancy”.ti, ab OR “Intestinal Malignancies”.ti, ab OR “Intestinal Malignancy”.ti, ab OR “Cecal Malignancies”.ti, ab OR “Cecal Malignancy”.ti, ab OR “Appenciceal Malignancies”.ti, ab OR “Appendiceal Malignancy”.ti, ab OR “Colorectal Malignancies”.ti, ab OR “Colorectal Malignancy”.ti, ab OR “Colonic Malignancies”.ti, ab OR “Colonic Malignancy”.ti, ab OR “Colon Malignancies”.ti, ab OR “Colon Malignancy”.ti, ab OR “Sigmoidal Malignancies”.ti, ab OR “Sigmoidal Malignancy”.ti, ab OR “Sigmoid Malignancies”.ti, ab OR “Sigmoid Malignancy”.ti, ab OR “Rectal Malignancies”.ti, ab OR “Rectal Malignancy”.ti, ab OR “Anorectal Malignancies”.ti, ab OR “Anorectal Malignancy”.ti, ab OR “Anus Malignancies”.ti, ab OR “Anus Malignancy”.ti, ab OR “Anal Malignancies”.ti, ab OR “Anal Malignancy”.ti, ab OR “Anal Gland Malignancies”.ti, ab OR “Anal Gland Malignancy”.ti, ab OR “Duodenal Malignancies”.ti, ab OR “Duodenal Malignancy”.ti, ab OR “Duodenum Malignancies”.ti, ab OR “Duodenum Malignancy”.ti, ab OR “Ileal Malignancies”.ti, ab OR “Ileal Malignancy”.ti, ab OR “Ileum Malignancies”.ti, ab OR “Ileum Malignancy”.ti, ab OR “Jejunal Malignancies”.ti, ab OR “Jejunal Malignancy”.ti, ab OR “Jejunum Malignancies”.ti, ab OR “Jejunum Malignancy”.ti, ab OR “Adenomatous Polyposis Coli”.ti, ab OR “Gardner Syndrome”.ti, ab) AND (“resection”.mp OR “resect*”.mp OR “surgery”.fs OR “surgery”.mp OR exp “surgery”/ OR “surgical”.mp OR exp “gastrectomy”/ OR “gastrectomy”.mp OR “gastrectom*”.mp OR exp *”Preoperative Period”/ OR exp *”Preoperative Care”/ OR “Preoperative Period”.ti, ab OR “Preoperative Care”.ti, ab OR exp *”Perioperative Period”/ OR exp *”Perioperative Care”/ OR “Perioperative Period”.ti, ab OR “Perioperative Care”.ti, ab OR exp *”Postoperative Period”/ OR exp *”Postoperative Care”/ OR “Postoperative Period”.ti, ab OR “Postoperative Care”.ti, ab OR “Preoperative”.ti, ab OR “Pre operative”.ti, ab OR “Perioperative”.ti, ab OR “Peri operative”.ti, ab OR “Postoperative”.ti, ab OR “Post operative”.ti, ab) AND (“immunonutrition “/ OR “immunonutrition”.mp OR “immunonutr*”.mp OR “immuno nutrition”.mp OR “immuno nutr*”.mp OR ((exp “Immunomodulation”/ OR “Immunomodulation”.mp OR “Immunomodulat*”.mp OR exp “Immunotherapy”/ OR “immunother*”.mp) AND (“Nutrition Therapy”.ti, ab OR exp *”Diet Therapy”/ OR “Diet Therapy”.ti, ab OR “Caloric Restriction”.ti, ab OR “Carbohydrate Loading diet”.ti, ab OR “Carbohydrate-Restricted diet”.ti, ab OR “High-Protein Low-Carbohydrate diet”.ti, ab OR “Ketogenic diet”.ti, ab OR “Diabetic diet”.ti, ab OR “Fat-Restricted diet”.ti, ab OR “Gluten-Free diet”.ti, ab OR “High-Protein diet”.ti, ab OR “High-Protein Low-Carbohydrate diet”.ti, ab OR “Mediterranean diet”.ti, ab OR “Paleolithic diet”.ti, ab OR “Protein-Restricted diet”.ti, ab OR “Reducing diet”.ti, ab OR “Sodium-Restricted diet”.ti, ab OR “Vegetarian diet”.ti, ab OR “Macrobiotic diet”.ti, ab OR “Vegan diet”.ti, ab OR “Carbohydrate Loading diets”.ti, ab OR “Carbohydrate-Restricted diets”.ti, ab OR “High-Protein Low-Carbohydrate diets”.ti, ab OR “Ketogenic diets”.ti, ab OR “Diabetic diets”.ti, ab OR “Fat-Restricted diets”.ti, ab OR “Gluten-Free diets”.ti, ab OR “High-Protein diets”.ti, ab OR “High-Protein Low-Carbohydrate diets”.ti, ab OR “Mediterranean diets”.ti, ab OR “Paleolithic diets”.ti, ab OR “Protein-Restricted diets”.ti, ab OR “Reducing diets”.ti, ab OR “Sodium-Restricted diets”.ti, ab OR “Vegetarian diets”.ti, ab OR “Macrobiotic diets”.ti, ab OR “Vegan diets”.ti, ab OR “Nutritional Support”.ti, ab OR “Enteral Nutrition”.ti, ab OR “Parenteral Nutrition”.ti, ab OR “Enteral Feeding”.ti, ab OR “Parenteral Feeding”.ti, ab OR “nutritional supplementation”.ti, ab OR “artifical nutrition”.ti, ab))) AND (exp “clinical trial”/ OR “clinical trial”.ti, ab OR exp “clinical trials as topic”/ OR “clinical trials”.ti, ab OR exp “control group”/ OR “control group”.ti, ab OR “control groups”.ti, ab OR exp “controlled clinical trial”/ OR exp “controlled clinical trials as topic”/ OR exp “crossover procedure”/ OR “cross over study”.ti, ab OR “cross over studies”.ti, ab OR exp “double-blind method”/ OR “double blind”.ti, ab OR exp “evaluation study”/ OR “follow up”/ OR “follow up study”.ti, ab OR “follow up studies”.ti, ab OR exp “placebo”/ OR placebo*.ti, ab OR placebos*.ti, ab OR exp “pragmatic clinical trial”/ OR exp “prospective study”/ OR “prospective study”.ti, ab OR “prospective studies”.ti, ab OR “RaCT”.ti, ab OR “RaCTs”.ti, ab OR exp “randomization”/ OR “randomised”.ti, ab OR exp “randomized controlled trial”/ OR exp “randomized controlled trials as topic”/ OR “randomized”.ti, ab OR random*.ti, ab OR “RCT”.ti, ab OR “RCTs”.ti, ab OR “single blind”.ti, ab OR exp “single blind procedure”/ OR ((single*.ti, ab OR double*.ti, ab OR triple*.ti, ab) ADJ3 (blind*.ti, ab OR mask*.ti, ab)) OR volunteer*.ti, ab OR “trial”.ti OR “trials”.ti))

**CENTRAL**

((“Head and Neck Neoplasms” OR “Head and Neck Neoplasms” OR “Head and Neck Neoplasm” OR “Ear Neoplasm” OR “Ear Neoplasms” OR “Eyelid Neoplasm” OR “Eyelid Neoplasms” OR “Facial Neoplasm” OR “Facial Neoplasms” OR “Gingival Neoplasm” OR “Gingival Neoplasms” OR “Hypopharyngeal Neoplasm” OR “Hypopharyngeal Neoplasms” OR “Laryngeal Neoplasm” OR “Laryngeal Neoplasms” OR “Lip Neoplasm” OR “Lip Neoplasms” OR “Maxillary Sinus Neoplasm” OR “Maxillary Sinus Neoplasms” OR “Mouth Neoplasm” OR “Mouth Neoplasms” OR “Nasopharyngeal Neoplasm” OR “Nasopharyngeal Neoplasms” OR “Nose Neoplasm” OR “Nose Neoplasms” OR “Oropharyngeal Neoplasm” OR “Oropharyngeal Neoplasms” OR “Otorhinolaryngologic Neoplasm” OR “Otorhinolaryngologic Neoplasms” OR “Palatal Neoplasm” OR “Palatal Neoplasms” OR “Papillary Thyroid Neoplasm” OR “Papillary Thyroid Neoplasms” OR “Paranasal Sinus Neoplasm” OR “Paranasal Sinus Neoplasms” OR “Parathyroid Neoplasm” OR “Parathyroid Neoplasms” OR “Parotid Neoplasm” OR “Parotid Neoplasms” OR “Pharyngeal Neoplasm” OR “Pharyngeal Neoplasms” OR “Salivary Gland Neoplasm” OR “Salivary Gland Neoplasms” OR “Sublingual Gland Neoplasm” OR “Sublingual Gland Neoplasms” OR “Submandibular Gland Neoplasm” OR “Submandibular Gland Neoplasms” OR “Thyroid Neoplasm” OR “Thyroid Neoplasms” OR “Tongue Neoplasm” OR “Tongue Neoplasms” OR “Tonsillar Neoplasm” OR “Tonsillar Neoplasms” OR “Tracheal Neoplasm” OR “Tracheal Neoplasms” OR “Head and Neck Cancers” OR “Head and Neck Cancer” OR “Ear Cancer” OR “Ear Cancers” OR “Eyelid Cancer” OR “Eyelid Cancers” OR “Facial Cancer” OR “Facial Cancers” OR “Gingival Cancer” OR “Gingival Cancers” OR “Hypopharyngeal Cancer” OR “Hypopharyngeal Cancers” OR “Laryngeal Cancer” OR “Laryngeal Cancers” OR “Lip Cancer” OR “Lip Cancers” OR “Maxillary Sinus Cancer” OR “Maxillary Sinus Cancers” OR “Mouth Cancer” OR “Mouth Cancers” OR “Nasopharyngeal Cancer” OR “Nasopharyngeal Cancers” OR “Nose Cancer” OR “Nose Cancers” OR “Oropharyngeal Cancer” OR “Oropharyngeal Cancers” OR “Otorhinolaryngologic Cancer” OR “Otorhinolaryngologic Cancers” OR “Palatal Cancer” OR “Palatal Cancers” OR “Papillary Thyroid Cancer” OR “Papillary Thyroid Cancers” OR “Paranasal Sinus Cancer” OR “Paranasal Sinus Cancers” OR “Parathyroid Cancer” OR “Parathyroid Cancers” OR “Parotid Cancer” OR “Parotid Cancers” OR “Pharyngeal Cancer” OR “Pharyngeal Cancers” OR “Salivary Gland Cancer” OR “Salivary Gland Cancers” OR “Sublingual Gland Cancer” OR “Sublingual Gland Cancers” OR “Submandibular Gland Cancer” OR “Submandibular Gland Cancers” OR “Thyroid Cancer” OR “Thyroid Cancers” OR “Tongue Cancer” OR “Tongue Cancers” OR “Tonsillar Cancer” OR “Tonsillar Cancers” OR “Tracheal Cancer” OR “Tracheal Cancers” OR “Head and Neck Carcinomas” OR “Head and Neck Carcinoma” OR “Ear Carcinoma” OR “Ear Carcinomas” OR “Eyelid Carcinoma” OR “Eyelid Carcinomas” OR “Facial Carcinoma” OR “Facial Carcinomas” OR “Gingival Carcinoma” OR “Gingival Carcinomas” OR “Hypopharyngeal Carcinoma” OR “Hypopharyngeal Carcinomas” OR “Laryngeal Carcinoma” OR “Laryngeal Carcinomas” OR “Lip Carcinoma” OR “Lip Carcinomas” OR “Maxillary Sinus Carcinoma” OR “Maxillary Sinus Carcinomas” OR “Mouth Carcinoma” OR “Mouth Carcinomas” OR “Nasopharyngeal Carcinoma” OR “Nasopharyngeal Carcinomas” OR “Nose Carcinoma” OR “Nose Carcinomas” OR “Oropharyngeal Carcinoma” OR “Oropharyngeal Carcinomas” OR “Otorhinolaryngologic Carcinoma” OR “Otorhinolaryngologic Carcinomas” OR “Palatal Carcinoma” OR “Palatal Carcinomas” OR “Papillary Thyroid Carcinoma” OR “Papillary Thyroid Carcinomas” OR “Paranasal Sinus Carcinoma” OR “Paranasal Sinus Carcinomas” OR “Parathyroid Carcinoma” OR “Parathyroid Carcinomas” OR “Parotid Carcinoma” OR “Parotid Carcinomas” OR “Pharyngeal Carcinoma” OR “Pharyngeal Carcinomas” OR “Salivary Gland Carcinoma” OR “Salivary Gland Carcinomas” OR “Sublingual Gland Carcinoma” OR “Sublingual Gland Carcinomas” OR “Submandibular Gland Carcinoma” OR “Submandibular Gland Carcinomas” OR “Thyroid Carcinoma” OR “Thyroid Carcinomas” OR “Tongue Carcinoma” OR “Tongue Carcinomas” OR “Tonsillar Carcinoma” OR “Tonsillar Carcinomas” OR “Tracheal Carcinoma” OR “Tracheal Carcinomas” OR “Head and Neck Adenocarcinomas” OR “Head and Neck Adenocarcinoma” OR “Ear Adenocarcinoma” OR “Ear Adenocarcinomas” OR “Eyelid Adenocarcinoma” OR “Eyelid Adenocarcinomas” OR “Facial Adenocarcinoma” OR “Facial Adenocarcinomas” OR “Gingival Adenocarcinoma” OR “Gingival Adenocarcinomas” OR “Hypopharyngeal Adenocarcinoma” OR “Hypopharyngeal Adenocarcinomas” OR “Laryngeal Adenocarcinoma” OR “Laryngeal Adenocarcinomas” OR “Lip Adenocarcinoma” OR “Lip Adenocarcinomas” OR “Maxillary Sinus Adenocarcinoma” OR “Maxillary Sinus Adenocarcinomas” OR “Mouth Adenocarcinoma” OR “Mouth Adenocarcinomas” OR “Nasopharyngeal Adenocarcinoma” OR “Nasopharyngeal Adenocarcinomas” OR “Nose Adenocarcinoma” OR “Nose Adenocarcinomas” OR “Oropharyngeal Adenocarcinoma” OR “Oropharyngeal Adenocarcinomas” OR “Otorhinolaryngologic Adenocarcinoma” OR “Otorhinolaryngologic Adenocarcinomas” OR “Palatal Adenocarcinoma” OR “Palatal Adenocarcinomas” OR “Papillary Thyroid Adenocarcinoma” OR “Papillary Thyroid Adenocarcinomas” OR “Paranasal Sinus Adenocarcinoma” OR “Paranasal Sinus Adenocarcinomas” OR “Parathyroid Adenocarcinoma” OR “Parathyroid Adenocarcinomas” OR “Parotid Adenocarcinoma” OR “Parotid Adenocarcinomas” OR “Pharyngeal Adenocarcinoma” OR “Pharyngeal Adenocarcinomas” OR “Salivary Gland Adenocarcinoma” OR “Salivary Gland Adenocarcinomas” OR “Sublingual Gland Adenocarcinoma” OR “Sublingual Gland Adenocarcinomas” OR “Submandibular Gland Adenocarcinoma” OR “Submandibular Gland Adenocarcinomas” OR “Thyroid Adenocarcinoma” OR “Thyroid Adenocarcinomas” OR “Tongue Adenocarcinoma” OR “Tongue Adenocarcinomas” OR “Tonsillar Adenocarcinoma” OR “Tonsillar Adenocarcinomas” OR “Tracheal Adenocarcinoma” OR “Tracheal Adenocarcinomas” OR “Head and Neck Tumors” OR “Head and Neck Tumor” OR “Ear Tumor” OR “Ear Tumors” OR “Eyelid Tumor” OR “Eyelid Tumors” OR “Facial Tumor” OR “Facial Tumors” OR “Gingival Tumor” OR “Gingival Tumors” OR “Hypopharyngeal Tumor” OR “Hypopharyngeal Tumors” OR “Laryngeal Tumor” OR “Laryngeal Tumors” OR “Lip Tumor” OR “Lip Tumors” OR “Maxillary Sinus Tumor” OR “Maxillary Sinus Tumors” OR “Mouth Tumor” OR “Mouth Tumors” OR “Nasopharyngeal Tumor” OR “Nasopharyngeal Tumors” OR “Nose Tumor” OR “Nose Tumors” OR “Oropharyngeal Tumor” OR “Oropharyngeal Tumors” OR “Otorhinolaryngologic Tumor” OR “Otorhinolaryngologic Tumors” OR “Palatal Tumor” OR “Palatal Tumors” OR “Papillary Thyroid Tumor” OR “Papillary Thyroid Tumors” OR “Paranasal Sinus Tumor” OR “Paranasal Sinus Tumors” OR “Parathyroid Tumor” OR “Parathyroid Tumors” OR “Parotid Tumor” OR “Parotid Tumors” OR “Pharyngeal Tumor” OR “Pharyngeal Tumors” OR “Salivary Gland Tumor” OR “Salivary Gland Tumors” OR “Sublingual Gland Tumor” OR “Sublingual Gland Tumors” OR “Submandibular Gland Tumor” OR “Submandibular Gland Tumors” OR “Thyroid Tumor” OR “Thyroid Tumors” OR “Tongue Tumor” OR “Tongue Tumors” OR “Tonsillar Tumor” OR “Tonsillar Tumors” OR “Tracheal Tumor” OR “Tracheal Tumors” OR “Head and Neck Tumours” OR “Head and Neck Tumour” OR “Ear Tumour” OR “Ear Tumours” OR “Eyelid Tumour” OR “Eyelid Tumours” OR “Facial Tumour” OR “Facial Tumours” OR “Gingival Tumour” OR “Gingival Tumours” OR “Hypopharyngeal Tumour” OR “Hypopharyngeal Tumours” OR “Laryngeal Tumour” OR “Laryngeal Tumours” OR “Lip Tumour” OR “Lip Tumours” OR “Maxillary Sinus Tumour” OR “Maxillary Sinus Tumours” OR “Mouth Tumour” OR “Mouth Tumours” OR “Nasopharyngeal Tumour” OR “Nasopharyngeal Tumours” OR “Nose Tumour” OR “Nose Tumours” OR “Oropharyngeal Tumour” OR “Oropharyngeal Tumours” OR “Otorhinolaryngologic Tumour” OR “Otorhinolaryngologic Tumours” OR “Palatal Tumour” OR “Palatal Tumours” OR “Papillary Thyroid Tumour” OR “Papillary Thyroid Tumours” OR “Paranasal Sinus Tumour” OR “Paranasal Sinus Tumours” OR “Parathyroid Tumour” OR “Parathyroid Tumours” OR “Parotid Tumour” OR “Parotid Tumours” OR “Pharyngeal Tumour” OR “Pharyngeal Tumours” OR “Salivary Gland Tumour” OR “Salivary Gland Tumours” OR “Sublingual Gland Tumour” OR “Sublingual Gland Tumours” OR “Submandibular Gland Tumour” OR “Submandibular Gland Tumours” OR “Thyroid Tumour” OR “Thyroid Tumours” OR “Tongue Tumour” OR “Tongue Tumours” OR “Tonsillar Tumour” OR “Tonsillar Tumours” OR “Tracheal Tumour” OR “Tracheal Tumours” OR “Head and Neck Malignancies” OR “Head and Neck Malignancy” OR “Ear Malignancy” OR “Ear Malignancies” OR “Eyelid Malignancy” OR “Eyelid Malignancies” OR “Facial Malignancy” OR “Facial Malignancies” OR “Gingival Malignancy” OR “Gingival Malignancies” OR “Hypopharyngeal Malignancy” OR “Hypopharyngeal Malignancies” OR “Laryngeal Malignancy” OR “Laryngeal Malignancies” OR “Lip Malignancy” OR “Lip Malignancies” OR “Maxillary Sinus Malignancy” OR “Maxillary Sinus Malignancies” OR “Mouth Malignancy” OR “Mouth Malignancies” OR “Nasopharyngeal Malignancy” OR “Nasopharyngeal Malignancies” OR “Nose Malignancy” OR “Nose Malignancies” OR “Oropharyngeal Malignancy” OR “Oropharyngeal Malignancies” OR “Otorhinolaryngologic Malignancy” OR “Otorhinolaryngologic Malignancies” OR “Palatal Malignancy” OR “Palatal Malignancies” OR “Papillary Thyroid Malignancy” OR “Papillary Thyroid Malignancies” OR “Paranasal Sinus Malignancy” OR “Paranasal Sinus Malignancies” OR “Parathyroid Malignancy” OR “Parathyroid Malignancies” OR “Parotid Malignancy” OR “Parotid Malignancies” OR “Pharyngeal Malignancy” OR “Pharyngeal Malignancies” OR “Salivary Gland Malignancy” OR “Salivary Gland Malignancies” OR “Sublingual Gland Malignancy” OR “Sublingual Gland Malignancies” OR “Submandibular Gland Malignancy” OR “Submandibular Gland Malignancies” OR “Thyroid Malignancy” OR “Thyroid Malignancies” OR “Tongue Malignancy” OR “Tongue Malignancies” OR “Tonsillar Malignancy” OR “Tonsillar Malignancies” OR “Tracheal Malignancy” OR “Tracheal Malignancies” OR “Thyroid Nodule” OR “Oral Leukoplakia” OR “Hairy Leukoplakia” OR “Gastrointestinal Neoplasms” OR “Gastrointestinal Neoplasms” OR “Gastrointestinal Neoplasm” OR “Stomach Neoplasms” OR “Stomach Neoplasm” OR “Gastric Neoplasms” OR “Gastric Neoplasm” OR “Esophageal Neoplasms” OR “Esophageal Neoplasm” OR “OEsophageal Neoplasms” OR “OEsophageal Neoplasm” OR “Esophagus Neoplasms” OR “Esophagus Neoplasm” OR “OEsophagus Neoplasms” OR “Esophagus Neoplasm” OR “Esophageal Squamus Cell Neoplasms” OR “Esophageal Squamus Cell Neoplasm” OR “OEsophageal Squamus Cell Neoplasms” OR “OEsophageal Squamus Cell Neoplasm” OR “Esophagus Squamus Cell Neoplasms” OR “Esophagus Squamus Cell Neoplasm” OR “OEsophagus Squamus Cell Neoplasms” OR “Esophagus Squamus Cell Neoplasm” OR “Intestinal Neoplasms” OR “Intestinal Neoplasm” OR “Cecal Neoplasms” OR “Cecal Neoplasm” OR “Appenciceal Neoplasms” OR “Appendiceal Neoplasm” OR “Colorectal Neoplasms” OR “Colorectal Neoplasm” OR “Colonic Neoplasms” OR “Colonic Neoplasm” OR “Colon Neoplasms” OR “Colon Neoplasm” OR “Sigmoidal Neoplasms” OR “Sigmoidal Neoplasm” OR “Sigmoid Neoplasms” OR “Sigmoid Neoplasm” OR “Rectal Neoplasms” OR “Rectal Neoplasm” OR “Anorectal Neoplasms” OR “Anorectal Neoplasm” OR “Anus Neoplasms” OR “Anus Neoplasm” OR “Anal Neoplasms” OR “Anal Neoplasm” OR “Anal Gland Neoplasms” OR “Anal Gland Neoplasm” OR “Duodenal Neoplasms” OR “Duodenal Neoplasm” OR “Duodenum Neoplasms” OR “Duodenum Neoplasm” OR “Ileal Neoplasms” OR “Ileal Neoplasm” OR “Ileum Neoplasms” OR “Ileum Neoplasm” OR “Jejunal Neoplasms” OR “Jejunal Neoplasm” OR “Jejunum Neoplasms” OR “Jejunum Neoplasm” OR “Gastrointestinal Cancers” OR “Gastrointestinal Cancer” OR “Stomach Cancers” OR “Stomach Cancer” OR “Gastric Cancers” OR “Gastric Cancer” OR “Esophageal Cancers” OR “Esophageal Cancer” OR “OEsophageal Cancers” OR “OEsophageal Cancer” OR “Esophagus Cancers” OR “Esophagus Cancer” OR “OEsophagus Cancers” OR “Esophagus Cancer” OR “Esophageal Squamus Cell Cancers” OR “Esophageal Squamus Cell Cancer” OR “OEsophageal Squamus Cell Cancers” OR “OEsophageal Squamus Cell Cancer” OR “Esophagus Squamus Cell Cancers” OR “Esophagus Squamus Cell Cancer” OR “OEsophagus Squamus Cell Cancers” OR “Esophagus Squamus Cell Cancer” OR “Intestinal Cancers” OR “Intestinal Cancer” OR “Cecal Cancers” OR “Cecal Cancer” OR “Appenciceal Cancers” OR “Appendiceal Cancer” OR “Colorectal Cancers” OR “Colorectal Cancer” OR “Colonic Cancers” OR “Colonic Cancer” OR “Colon Cancers” OR “Colon Cancer” OR “Sigmoidal Cancers” OR “Sigmoidal Cancer” OR “Sigmoid Cancers” OR “Sigmoid Cancer” OR “Rectal Cancers” OR “Rectal Cancer” OR “Anorectal Cancers” OR “Anorectal Cancer” OR “Anus Cancers” OR “Anus Cancer” OR “Anal Cancers” OR “Anal Cancer” OR “Anal Gland Cancers” OR “Anal Gland Cancer” OR “Duodenal Cancers” OR “Duodenal Cancer” OR “Duodenum Cancers” OR “Duodenum Cancer” OR “Ileal Cancers” OR “Ileal Cancer” OR “Ileum Cancers” OR “Ileum Cancer” OR “Jejunal Cancers” OR “Jejunal Cancer” OR “Jejunum Cancers” OR “Jejunum Cancer” OR “Gastrointestinal Carcinomas” OR “Gastrointestinal Carcinoma” OR “Stomach Carcinomas” OR “Stomach Carcinoma” OR “Gastric Carcinomas” OR “Gastric Carcinoma” OR “Esophageal Carcinomas” OR “Esophageal Carcinoma” OR “OEsophageal Carcinomas” OR “OEsophageal Carcinoma” OR “Esophagus Carcinomas” OR “Esophagus Carcinoma” OR “OEsophagus Carcinomas” OR “Esophagus Carcinoma” OR “Esophageal Squamus Cell Carcinomas” OR “Esophageal Squamus Cell Carcinoma” OR “OEsophageal Squamus Cell Carcinomas” OR “OEsophageal Squamus Cell Carcinoma” OR “Esophagus Squamus Cell Carcinomas” OR “Esophagus Squamus Cell Carcinoma” OR “OEsophagus Squamus Cell Carcinomas” OR “Esophagus Squamus Cell Carcinoma” OR “Intestinal Carcinomas” OR “Intestinal Carcinoma” OR “Cecal Carcinomas” OR “Cecal Carcinoma” OR “Appenciceal Carcinomas” OR “Appendiceal Carcinoma” OR “Colorectal Carcinomas” OR “Colorectal Carcinoma” OR “Colonic Carcinomas” OR “Colonic Carcinoma” OR “Colon Carcinomas” OR “Colon Carcinoma” OR “Sigmoidal Carcinomas” OR “Sigmoidal Carcinoma” OR “Sigmoid Carcinomas” OR “Sigmoid Carcinoma” OR “Rectal Carcinomas” OR “Rectal Carcinoma” OR “Anorectal Carcinomas” OR “Anorectal Carcinoma” OR “Anus Carcinomas” OR “Anus Carcinoma” OR “Anal Carcinomas” OR “Anal Carcinoma” OR “Anal Gland Carcinomas” OR “Anal Gland Carcinoma” OR “Duodenal Carcinomas” OR “Duodenal Carcinoma” OR “Duodenum Carcinomas” OR “Duodenum Carcinoma” OR “Ileal Carcinomas” OR “Ileal Carcinoma” OR “Ileum Carcinomas” OR “Ileum Carcinoma” OR “Jejunal Carcinomas” OR “Jejunal Carcinoma” OR “Jejunum Carcinomas” OR “Jejunum Carcinoma” OR “Gastrointestinal Adenocarcinomas” OR “Gastrointestinal Adenocarcinoma” OR “Stomach Adenocarcinomas” OR “Stomach Adenocarcinoma” OR “Gastric Adenocarcinomas” OR “Gastric Adenocarcinoma” OR “Esophageal Adenocarcinomas” OR “Esophageal Adenocarcinoma” OR “OEsophageal Adenocarcinomas” OR “OEsophageal Adenocarcinoma” OR “Esophagus Adenocarcinomas” OR “Esophagus Adenocarcinoma” OR “OEsophagus Adenocarcinomas” OR “Esophagus Adenocarcinoma” OR “Esophageal Squamus Cell Adenocarcinomas” OR “Esophageal Squamus Cell Adenocarcinoma” OR “OEsophageal Squamus Cell Adenocarcinomas” OR “OEsophageal Squamus Cell Adenocarcinoma” OR “Esophagus Squamus Cell Adenocarcinomas” OR “Esophagus Squamus Cell Adenocarcinoma” OR “OEsophagus Squamus Cell Adenocarcinomas” OR “Esophagus Squamus Cell Adenocarcinoma” OR “Intestinal Adenocarcinomas” OR “Intestinal Adenocarcinoma” OR “Cecal Adenocarcinomas” OR “Cecal Adenocarcinoma” OR “Appenciceal Adenocarcinomas” OR “Appendiceal Adenocarcinoma” OR “Colorectal Adenocarcinomas” OR “Colorectal Adenocarcinoma” OR “Colonic Adenocarcinomas” OR “Colonic Adenocarcinoma” OR “Colon Adenocarcinomas” OR “Colon Adenocarcinoma” OR “Sigmoidal Adenocarcinomas” OR “Sigmoidal Adenocarcinoma” OR “Sigmoid Adenocarcinomas” OR “Sigmoid Adenocarcinoma” OR “Rectal Adenocarcinomas” OR “Rectal Adenocarcinoma” OR “Anorectal Adenocarcinomas” OR “Anorectal Adenocarcinoma” OR “Anus Adenocarcinomas” OR “Anus Adenocarcinoma” OR “Anal Adenocarcinomas” OR “Anal Adenocarcinoma” OR “Anal Gland Adenocarcinomas” OR “Anal Gland Adenocarcinoma” OR “Duodenal Adenocarcinomas” OR “Duodenal Adenocarcinoma” OR “Duodenum Adenocarcinomas” OR “Duodenum Adenocarcinoma” OR “Ileal Adenocarcinomas” OR “Ileal Adenocarcinoma” OR “Ileum Adenocarcinomas” OR “Ileum Adenocarcinoma” OR “Jejunal Adenocarcinomas” OR “Jejunal Adenocarcinoma” OR “Jejunum Adenocarcinomas” OR “Jejunum Adenocarcinoma” OR “Gastrointestinal Tumors” OR “Gastrointestinal Tumor” OR “Stomach Tumors” OR “Stomach Tumor” OR “Gastric Tumors” OR “Gastric Tumor” OR “Esophageal Tumors” OR “Esophageal Tumor” OR “OEsophageal Tumors” OR “OEsophageal Tumor” OR “Esophagus Tumors” OR “Esophagus Tumor” OR “OEsophagus Tumors” OR “Esophagus Tumor” OR “Esophageal Squamus Cell Tumors” OR “Esophageal Squamus Cell Tumor” OR “OEsophageal Squamus Cell Tumors” OR “OEsophageal Squamus Cell Tumor” OR “Esophagus Squamus Cell Tumors” OR “Esophagus Squamus Cell Tumor” OR “OEsophagus Squamus Cell Tumors” OR “Esophagus Squamus Cell Tumor” OR “Intestinal Tumors” OR “Intestinal Tumor” OR “Cecal Tumors” OR “Cecal Tumor” OR “Appenciceal Tumors” OR “Appendiceal Tumor” OR “Colorectal Tumors” OR “Colorectal Tumor” OR “Colonic Tumors” OR “Colonic Tumor” OR “Colon Tumors” OR “Colon Tumor” OR “Sigmoidal Tumors” OR “Sigmoidal Tumor” OR “Sigmoid Tumors” OR “Sigmoid Tumor” OR “Rectal Tumors” OR “Rectal Tumor” OR “Anorectal Tumors” OR “Anorectal Tumor” OR “Anus Tumors” OR “Anus Tumor” OR “Anal Tumors” OR “Anal Tumor” OR “Anal Gland Tumors” OR “Anal Gland Tumor” OR “Duodenal Tumors” OR “Duodenal Tumor” OR “Duodenum Tumors” OR “Duodenum Tumor” OR “Ileal Tumors” OR “Ileal Tumor” OR “Ileum Tumors” OR “Ileum Tumor” OR “Jejunal Tumors” OR “Jejunal Tumor” OR “Jejunum Tumors” OR “Jejunum Tumor” OR “Gastrointestinal Tumours” OR “Gastrointestinal Tumour” OR “Stomach Tumours” OR “Stomach Tumour” OR “Gastric Tumours” OR “Gastric Tumour” OR “Esophageal Tumours” OR “Esophageal Tumour” OR “OEsophageal Tumours” OR “OEsophageal Tumour” OR “Esophagus Tumours” OR “Esophagus Tumour” OR “OEsophagus Tumours” OR “Esophagus Tumour” OR “Esophageal Squamus Cell Tumours” OR “Esophageal Squamus Cell Tumour” OR “OEsophageal Squamus Cell Tumours” OR “OEsophageal Squamus Cell Tumour” OR “Esophagus Squamus Cell Tumours” OR “Esophagus Squamus Cell Tumour” OR “OEsophagus Squamus Cell Tumours” OR “Esophagus Squamus Cell Tumour” OR “Intestinal Tumours” OR “Intestinal Tumour” OR “Cecal Tumours” OR “Cecal Tumour” OR “Appenciceal Tumours” OR “Appendiceal Tumour” OR “Colorectal Tumours” OR “Colorectal Tumour” OR “Colonic Tumours” OR “Colonic Tumour” OR “Colon Tumours” OR “Colon Tumour” OR “Sigmoidal Tumours” OR “Sigmoidal Tumour” OR “Sigmoid Tumours” OR “Sigmoid Tumour” OR “Rectal Tumours” OR “Rectal Tumour” OR “Anorectal Tumours” OR “Anorectal Tumour” OR “Anus Tumours” OR “Anus Tumour” OR “Anal Tumours” OR “Anal Tumour” OR “Anal Gland Tumours” OR “Anal Gland Tumour” OR “Duodenal Tumours” OR “Duodenal Tumour” OR “Duodenum Tumours” OR “Duodenum Tumour” OR “Ileal Tumours” OR “Ileal Tumour” OR “Ileum Tumours” OR “Ileum Tumour” OR “Jejunal Tumours” OR “Jejunal Tumour” OR “Jejunum Tumours” OR “Jejunum Tumour” OR “Gastrointestinal Malignancies” OR “Gastrointestinal Malignancy” OR “Stomach Malignancies” OR “Stomach Malignancy” OR “Gastric Malignancies” OR “Gastric Malignancy” OR “Esophageal Malignancies” OR “Esophageal Malignancy” OR “OEsophageal Malignancies” OR “OEsophageal Malignancy” OR “Esophagus Malignancies” OR “Esophagus Malignancy” OR “OEsophagus Malignancies” OR “Esophagus Malignancy” OR “Esophageal Squamus Cell Malignancies” OR “Esophageal Squamus Cell Malignancy” OR “OEsophageal Squamus Cell Malignancies” OR “OEsophageal Squamus Cell Malignancy” OR “Esophagus Squamus Cell Malignancies” OR “Esophagus Squamus Cell Malignancy” OR “OEsophagus Squamus Cell Malignancies” OR “Esophagus Squamus Cell Malignancy” OR “Intestinal Malignancies” OR “Intestinal Malignancy” OR “Cecal Malignancies” OR “Cecal Malignancy” OR “Appenciceal Malignancies” OR “Appendiceal Malignancy” OR “Colorectal Malignancies” OR “Colorectal Malignancy” OR “Colonic Malignancies” OR “Colonic Malignancy” OR “Colon Malignancies” OR “Colon Malignancy” OR “Sigmoidal Malignancies” OR “Sigmoidal Malignancy” OR “Sigmoid Malignancies” OR “Sigmoid Malignancy” OR “Rectal Malignancies” OR “Rectal Malignancy” OR “Anorectal Malignancies” OR “Anorectal Malignancy” OR “Anus Malignancies” OR “Anus Malignancy” OR “Anal Malignancies” OR “Anal Malignancy” OR “Anal Gland Malignancies” OR “Anal Gland Malignancy” OR “Duodenal Malignancies” OR “Duodenal Malignancy” OR “Duodenum Malignancies” OR “Duodenum Malignancy” OR “Ileal Malignancies” OR “Ileal Malignancy” OR “Ileum Malignancies” OR “Ileum Malignancy” OR “Jejunal Malignancies” OR “Jejunal Malignancy” OR “Jejunum Malignancies” OR “Jejunum Malignancy” OR “Adenomatous Polyposis Coli” OR “Gardner Syndrome”) AND (“resection” OR “resect*” OR “surgery” OR “surgical” OR “gastrectomy” OR “gastrectomy” OR “gastrectom*” OR “Preoperative Period” OR “Preoperative Care” OR “Preoperative Period” OR “Preoperative Care” OR “Perioperative Period” OR “Perioperative Care” OR “Perioperative Period” OR “Perioperative Care” OR “Postoperative Period” OR “Postoperative Care” OR “Postoperative Period” OR “Postoperative Care” OR “Preoperative” OR “Pre operative” OR “Perioperative” OR “Peri operative” OR “Postoperative” OR “Post operative”) AND (“immunonutrition “ OR “immunonutrition” OR “immunonutr*” OR “immuno nutrition” OR “immuno nutr*” OR ((“Immunomodulation” OR “Immunomodulation” OR “Immunomodulat*” OR “Immunotherapy” OR “immunother*”) AND (“Nutrition Therapy” OR “Nutrition Therapy” OR “Diet Therapy” OR “Diet Therapy” OR “Caloric Restriction” OR “Carbohydrate Loading diet” OR “Carbohydrate Restricted diet” OR “High Protein Low Carbohydrate diet” OR “Ketogenic diet” OR “Diabetic diet” OR “Fat Restricted diet” OR “Gluten Free diet” OR “High Protein diet” OR “High Protein Low Carbohydrate diet” OR “Mediterranean diet” OR “Paleolithic diet” OR “Protein Restricted diet” OR “Reducing diet” OR “Sodium Restricted diet” OR “Vegetarian diet” OR “Macrobiotic diet” OR “Vegan diet” OR “Carbohydrate Loading diets” OR “Carbohydrate Restricted diets” OR “High Protein Low Carbohydrate diets” OR “Ketogenic diets” OR “Diabetic diets” OR “Fat Restricted diets” OR “Gluten Free diets” OR “High Protein diets” OR “High Protein Low Carbohydrate diets” OR “Mediterranean diets” OR “Paleolithic diets” OR “Protein Restricted diets” OR “Reducing diets” OR “Sodium Restricted diets” OR “Vegetarian diets” OR “Macrobiotic diets” OR “Vegan diets” OR “Nutritional Support” OR “Enteral Nutrition” OR “Parenteral Nutrition” OR “Enteral Feeding” OR “Parenteral Feeding” OR “nutritional supplementation” OR “artifical nutrition” OR “Diet, Food, and Nutrition”)))):ti, ab, kw

**Emcare via OVID**

((exp *”Head and Neck Tumor”/ OR “Head and Neck Neoplasms”.ti, ab OR “Head and Neck Neoplasm”.ti, ab OR “Ear Neoplasm”.ti, ab OR “Ear Neoplasms”.ti, ab OR “Eyelid Neoplasm”.ti, ab OR “Eyelid Neoplasms”.ti, ab OR “Facial Neoplasm”.ti, ab OR “Facial Neoplasms”.ti, ab OR “Gingival Neoplasm”.ti, ab OR “Gingival Neoplasms”.ti, ab OR “Hypopharyngeal Neoplasm”.ti, ab OR “Hypopharyngeal Neoplasms”.ti, ab OR “Laryngeal Neoplasm”.ti, ab OR “Laryngeal Neoplasms”.ti, ab OR “Lip Neoplasm”.ti, ab OR “Lip Neoplasms”.ti, ab OR “Maxillary Sinus Neoplasm”.ti, ab OR “Maxillary Sinus Neoplasms”.ti, ab OR “Mouth Neoplasm”.ti, ab OR “Mouth Neoplasms”.ti, ab OR “Nasopharyngeal Neoplasm”.ti, ab OR “Nasopharyngeal Neoplasms”.ti, ab OR “Nose Neoplasm”.ti, ab OR “Nose Neoplasms”.ti, ab OR “Oropharyngeal Neoplasm”.ti, ab OR “Oropharyngeal Neoplasms”.ti, ab OR “Otorhinolaryngologic Neoplasm”.ti, ab OR “Otorhinolaryngologic Neoplasms”.ti, ab OR “Palatal Neoplasm”.ti, ab OR “Palatal Neoplasms”.ti, ab OR “Papillary Thyroid Neoplasm “.ti, ab OR “Papillary Thyroid Neoplasms “.ti, ab OR “Paranasal Sinus Neoplasm”.ti, ab OR “Paranasal Sinus Neoplasms”.ti, ab OR “Parathyroid Neoplasm”.ti, ab OR “Parathyroid Neoplasms”.ti, ab OR “Parotid Neoplasm”.ti, ab OR “Parotid Neoplasms”.ti, ab OR “Pharyngeal Neoplasm”.ti, ab OR “Pharyngeal Neoplasms”.ti, ab OR “Salivary Gland Neoplasm”.ti, ab OR “Salivary Gland Neoplasms”.ti, ab OR “Sublingual Gland Neoplasm”.ti, ab OR “Sublingual Gland Neoplasms”.ti, ab OR “Submandibular Gland Neoplasm”.ti, ab OR “Submandibular Gland Neoplasms”.ti, ab OR “Thyroid Neoplasm”.ti, ab OR “Thyroid Neoplasms”.ti, ab OR “Tongue Neoplasm”.ti, ab OR “Tongue Neoplasms”.ti, ab OR “Tonsillar Neoplasm”.ti, ab OR “Tonsillar Neoplasms”.ti, ab OR “Tracheal Neoplasm”.ti, ab OR “Tracheal Neoplasms”.ti, ab OR “Head and Neck Cancers”.ti, ab OR “Head and Neck Cancer”.ti, ab OR “Ear Cancer”.ti, ab OR “Ear Cancers”.ti, ab OR “Eyelid Cancer”.ti, ab OR “Eyelid Cancers”.ti, ab OR “Facial Cancer”.ti, ab OR “Facial Cancers”.ti, ab OR “Gingival Cancer”.ti, ab OR “Gingival Cancers”.ti, ab OR “Hypopharyngeal Cancer”.ti, ab OR “Hypopharyngeal Cancers”.ti, ab OR “Laryngeal Cancer”.ti, ab OR “Laryngeal Cancers”.ti, ab OR “Lip Cancer”.ti, ab OR “Lip Cancers”.ti, ab OR “Maxillary Sinus Cancer”.ti, ab OR “Maxillary Sinus Cancers”.ti, ab OR “Mouth Cancer”.ti, ab OR “Mouth Cancers”.ti, ab OR “Nasopharyngeal Cancer”.ti, ab OR “Nasopharyngeal Cancers”.ti, ab OR “Nose Cancer”.ti, ab OR “Nose Cancers”.ti, ab OR “Oropharyngeal Cancer”.ti, ab OR “Oropharyngeal Cancers”.ti, ab OR “Otorhinolaryngologic Cancer”.ti, ab OR “Otorhinolaryngologic Cancers”.ti, ab OR “Palatal Cancer”.ti, ab OR “Palatal Cancers”.ti, ab OR “Papillary Thyroid Cancer “.ti, ab OR “Papillary Thyroid Cancers “.ti, ab OR “Paranasal Sinus Cancer”.ti, ab OR “Paranasal Sinus Cancers”.ti, ab OR “Parathyroid Cancer”.ti, ab OR “Parathyroid Cancers”.ti, ab OR “Parotid Cancer”.ti, ab OR “Parotid Cancers”.ti, ab OR “Pharyngeal Cancer”.ti, ab OR “Pharyngeal Cancers”.ti, ab OR “Salivary Gland Cancer”.ti, ab OR “Salivary Gland Cancers”.ti, ab OR “Sublingual Gland Cancer”.ti, ab OR “Sublingual Gland Cancers”.ti, ab OR “Submandibular Gland Cancer”.ti, ab OR “Submandibular Gland Cancers”.ti, ab OR “Thyroid Cancer”.ti, ab OR “Thyroid Cancers”.ti, ab OR “Tongue Cancer”.ti, ab OR “Tongue Cancers”.ti, ab OR “Tonsillar Cancer”.ti, ab OR “Tonsillar Cancers”.ti, ab OR “Tracheal Cancer”.ti, ab OR “Tracheal Cancers”.ti, ab OR “Head and Neck Carcinomas”.ti, ab OR “Head and Neck Carcinoma”.ti, ab OR “Ear Carcinoma”.ti, ab OR “Ear Carcinomas”.ti, ab OR “Eyelid Carcinoma”.ti, ab OR “Eyelid Carcinomas”.ti, ab OR “Facial Carcinoma”.ti, ab OR “Facial Carcinomas”.ti, ab OR “Gingival Carcinoma”.ti, ab OR “Gingival Carcinomas”.ti, ab OR “Hypopharyngeal Carcinoma”.ti, ab OR “Hypopharyngeal Carcinomas”.ti, ab OR “Laryngeal Carcinoma”.ti, ab OR “Laryngeal Carcinomas”.ti, ab OR “Lip Carcinoma”.ti, ab OR “Lip Carcinomas”.ti, ab OR “Maxillary Sinus Carcinoma”.ti, ab OR “Maxillary Sinus Carcinomas”.ti, ab OR “Mouth Carcinoma”.ti, ab OR “Mouth Carcinomas”.ti, ab OR “Nasopharyngeal Carcinoma”.ti, ab OR “Nasopharyngeal Carcinomas”.ti, ab OR “Nose Carcinoma”.ti, ab OR “Nose Carcinomas”.ti, ab OR “Oropharyngeal Carcinoma”.ti, ab OR “Oropharyngeal Carcinomas”.ti, ab OR “Otorhinolaryngologic Carcinoma”.ti, ab OR “Otorhinolaryngologic Carcinomas”.ti, ab OR “Palatal Carcinoma”.ti, ab OR “Palatal Carcinomas”.ti, ab OR “Papillary Thyroid Carcinoma “.ti, ab OR “Papillary Thyroid Carcinomas “.ti, ab OR “Paranasal Sinus Carcinoma”.ti, ab OR “Paranasal Sinus Carcinomas”.ti, ab OR “Parathyroid Carcinoma”.ti, ab OR “Parathyroid Carcinomas”.ti, ab OR “Parotid Carcinoma”.ti, ab OR “Parotid Carcinomas”.ti, ab OR “Pharyngeal Carcinoma”.ti, ab OR “Pharyngeal Carcinomas”.ti, ab OR “Salivary Gland Carcinoma”.ti, ab OR “Salivary Gland Carcinomas”.ti, ab OR “Sublingual Gland Carcinoma”.ti, ab OR “Sublingual Gland Carcinomas”.ti, ab OR “Submandibular Gland Carcinoma”.ti, ab OR “Submandibular Gland Carcinomas”.ti, ab OR “Thyroid Carcinoma”.ti, ab OR “Thyroid Carcinomas”.ti, ab OR “Tongue Carcinoma”.ti, ab OR “Tongue Carcinomas”.ti, ab OR “Tonsillar Carcinoma”.ti, ab OR “Tonsillar Carcinomas”.ti, ab OR “Tracheal Carcinoma”.ti, ab OR “Tracheal Carcinomas”.ti, ab OR “Head and Neck Adenocarcinomas”.ti, ab OR “Head and Neck Adenocarcinoma”.ti, ab OR “Ear Adenocarcinoma”.ti, ab OR “Ear Adenocarcinomas”.ti, ab OR “Eyelid Adenocarcinoma”.ti, ab OR “Eyelid Adenocarcinomas”.ti, ab OR “Facial Adenocarcinoma”.ti, ab OR “Facial Adenocarcinomas”.ti, ab OR “Gingival Adenocarcinoma”.ti, ab OR “Gingival Adenocarcinomas”.ti, ab OR “Hypopharyngeal Adenocarcinoma”.ti, ab OR “Hypopharyngeal Adenocarcinomas”.ti, ab OR “Laryngeal Adenocarcinoma”.ti, ab OR “Laryngeal Adenocarcinomas”.ti, ab OR “Lip Adenocarcinoma”.ti, ab OR “Lip Adenocarcinomas”.ti, ab OR “Maxillary Sinus Adenocarcinoma”.ti, ab OR “Maxillary Sinus Adenocarcinomas”.ti, ab OR “Mouth Adenocarcinoma”.ti, ab OR “Mouth Adenocarcinomas”.ti, ab OR “Nasopharyngeal Adenocarcinoma”.ti, ab OR “Nasopharyngeal Adenocarcinomas”.ti, ab OR “Nose Adenocarcinoma”.ti, ab OR “Nose Adenocarcinomas”.ti, ab OR “Oropharyngeal Adenocarcinoma”.ti, ab OR “Oropharyngeal Adenocarcinomas”.ti, ab OR “Otorhinolaryngologic Adenocarcinoma”.ti, ab OR “Otorhinolaryngologic Adenocarcinomas”.ti, ab OR “Palatal Adenocarcinoma”.ti, ab OR “Palatal Adenocarcinomas”.ti, ab OR “Papillary Thyroid Adenocarcinoma “.ti, ab OR “Papillary Thyroid Adenocarcinomas “.ti, ab OR “Paranasal Sinus Adenocarcinoma”.ti, ab OR “Paranasal Sinus Adenocarcinomas”.ti, ab OR “Parathyroid Adenocarcinoma”.ti, ab OR “Parathyroid Adenocarcinomas”.ti, ab OR “Parotid Adenocarcinoma”.ti, ab OR “Parotid Adenocarcinomas”.ti, ab OR “Pharyngeal Adenocarcinoma”.ti, ab OR “Pharyngeal Adenocarcinomas”.ti, ab OR “Salivary Gland Adenocarcinoma”.ti, ab OR “Salivary Gland Adenocarcinomas”.ti, ab OR “Sublingual Gland Adenocarcinoma”.ti, ab OR “Sublingual Gland Adenocarcinomas”.ti, ab OR “Submandibular Gland Adenocarcinoma”.ti, ab OR “Submandibular Gland Adenocarcinomas”.ti, ab OR “Thyroid Adenocarcinoma”.ti, ab OR “Thyroid Adenocarcinomas”.ti, ab OR “Tongue Adenocarcinoma”.ti, ab OR “Tongue Adenocarcinomas”.ti, ab OR “Tonsillar Adenocarcinoma”.ti, ab OR “Tonsillar Adenocarcinomas”.ti, ab OR “Tracheal Adenocarcinoma”.ti, ab OR “Tracheal Adenocarcinomas”.ti, ab OR “Head and Neck Tumors”.ti, ab OR “Head and Neck Tumor”.ti, ab OR “Ear Tumor”.ti, ab OR “Ear Tumors”.ti, ab OR “Eyelid Tumor”.ti, ab OR “Eyelid Tumors”.ti, ab OR “Facial Tumor”.ti, ab OR “Facial Tumors”.ti, ab OR “Gingival Tumor”.ti, ab OR “Gingival Tumors”.ti, ab OR “Hypopharyngeal Tumor”.ti, ab OR “Hypopharyngeal Tumors”.ti, ab OR “Laryngeal Tumor”.ti, ab OR “Laryngeal Tumors”.ti, ab OR “Lip Tumor”.ti, ab OR “Lip Tumors”.ti, ab OR “Maxillary Sinus Tumor”.ti, ab OR “Maxillary Sinus Tumors”.ti, ab OR “Mouth Tumor”.ti, ab OR “Mouth Tumors”.ti, ab OR “Nasopharyngeal Tumor”.ti, ab OR “Nasopharyngeal Tumors”.ti, ab OR “Nose Tumor”.ti, ab OR “Nose Tumors”.ti, ab OR “Oropharyngeal Tumor”.ti, ab OR “Oropharyngeal Tumors”.ti, ab OR “Otorhinolaryngologic Tumor”.ti, ab OR “Otorhinolaryngologic Tumors”.ti, ab OR “Palatal Tumor”.ti, ab OR “Palatal Tumors”.ti, ab OR “Papillary Thyroid Tumor “.ti, ab OR “Papillary Thyroid Tumors “.ti, ab OR “Paranasal Sinus Tumor”.ti, ab OR “Paranasal Sinus Tumors”.ti, ab OR “Parathyroid Tumor”.ti, ab OR “Parathyroid Tumors”.ti, ab OR “Parotid Tumor”.ti, ab OR “Parotid Tumors”.ti, ab OR “Pharyngeal Tumor”.ti, ab OR “Pharyngeal Tumors”.ti, ab OR “Salivary Gland Tumor”.ti, ab OR “Salivary Gland Tumors”.ti, ab OR “Sublingual Gland Tumor”.ti, ab OR “Sublingual Gland Tumors”.ti, ab OR “Submandibular Gland Tumor”.ti, ab OR “Submandibular Gland Tumors”.ti, ab OR “Thyroid Tumor”.ti, ab OR “Thyroid Tumors”.ti, ab OR “Tongue Tumor”.ti, ab OR “Tongue Tumors”.ti, ab OR “Tonsillar Tumor”.ti, ab OR “Tonsillar Tumors”.ti, ab OR “Tracheal Tumor”.ti, ab OR “Tracheal Tumors”.ti, ab OR “Head and Neck Tumours”.ti, ab OR “Head and Neck Tumour”.ti, ab OR “Ear Tumour”.ti, ab OR “Ear Tumours”.ti, ab OR “Eyelid Tumour”.ti, ab OR “Eyelid Tumours”.ti, ab OR “Facial Tumour”.ti, ab OR “Facial Tumours”.ti, ab OR “Gingival Tumour”.ti, ab OR “Gingival Tumours”.ti, ab OR “Hypopharyngeal Tumour”.ti, ab OR “Hypopharyngeal Tumours”.ti, ab OR “Laryngeal Tumour”.ti, ab OR “Laryngeal Tumours”.ti, ab OR “Lip Tumour”.ti, ab OR “Lip Tumours”.ti, ab OR “Maxillary Sinus Tumour”.ti, ab OR “Maxillary Sinus Tumours”.ti, ab OR “Mouth Tumour”.ti, ab OR “Mouth Tumours”.ti, ab OR “Nasopharyngeal Tumour”.ti, ab OR “Nasopharyngeal Tumours”.ti, ab OR “Nose Tumour”.ti, ab OR “Nose Tumours”.ti, ab OR “Oropharyngeal Tumour”.ti, ab OR “Oropharyngeal Tumours”.ti, ab OR “Otorhinolaryngologic Tumour”.ti, ab OR “Otorhinolaryngologic Tumours”.ti, ab OR “Palatal Tumour”.ti, ab OR “Palatal Tumours”.ti, ab OR “Papillary Thyroid Tumour “.ti, ab OR “Papillary Thyroid Tumours “.ti, ab OR “Paranasal Sinus Tumour”.ti, ab OR “Paranasal Sinus Tumours”.ti, ab OR “Parathyroid Tumour”.ti, ab OR “Parathyroid Tumours”.ti, ab OR “Parotid Tumour”.ti, ab OR “Parotid Tumours”.ti, ab OR “Pharyngeal Tumour”.ti, ab OR “Pharyngeal Tumours”.ti, ab OR “Salivary Gland Tumour”.ti, ab OR “Salivary Gland Tumours”.ti, ab OR “Sublingual Gland Tumour”.ti, ab OR “Sublingual Gland Tumours”.ti, ab OR “Submandibular Gland Tumour”.ti, ab OR “Submandibular Gland Tumours”.ti, ab OR “Thyroid Tumour”.ti, ab OR “Thyroid Tumours”.ti, ab OR “Tongue Tumour”.ti, ab OR “Tongue Tumours”.ti, ab OR “Tonsillar Tumour”.ti, ab OR “Tonsillar Tumours”.ti, ab OR “Tracheal Tumour”.ti, ab OR “Tracheal Tumours”.ti, ab OR “Head and Neck Malignancies”.ti, ab OR “Head and Neck Malignancy”.ti, ab OR “Ear Malignancy”.ti, ab OR “Ear Malignancies”.ti, ab OR “Eyelid Malignancy”.ti, ab OR “Eyelid Malignancies”.ti, ab OR “Facial Malignancy”.ti, ab OR “Facial Malignancies”.ti, ab OR “Gingival Malignancy”.ti, ab OR “Gingival Malignancies”.ti, ab OR “Hypopharyngeal Malignancy”.ti, ab OR “Hypopharyngeal Malignancies”.ti, ab OR “Laryngeal Malignancy”.ti, ab OR “Laryngeal Malignancies”.ti, ab OR “Lip Malignancy”.ti, ab OR “Lip Malignancies”.ti, ab OR “Maxillary Sinus Malignancy”.ti, ab OR “Maxillary Sinus Malignancies”.ti, ab OR “Mouth Malignancy”.ti, ab OR “Mouth Malignancies”.ti, ab OR “Nasopharyngeal Malignancy”.ti, ab OR “Nasopharyngeal Malignancies”.ti, ab OR “Nose Malignancy”.ti, ab OR “Nose Malignancies”.ti, ab OR “Oropharyngeal Malignancy”.ti, ab OR “Oropharyngeal Malignancies”.ti, ab OR “Otorhinolaryngologic Malignancy”.ti, ab OR “Otorhinolaryngologic Malignancies”.ti, ab OR “Palatal Malignancy”.ti, ab OR “Palatal Malignancies”.ti, ab OR “Papillary Thyroid Malignancy “.ti, ab OR “Papillary Thyroid Malignancies “.ti, ab OR “Paranasal Sinus Malignancy”.ti, ab OR “Paranasal Sinus Malignancies”.ti, ab OR “Parathyroid Malignancy”.ti, ab OR “Parathyroid Malignancies”.ti, ab OR “Parotid Malignancy”.ti, ab OR “Parotid Malignancies”.ti, ab OR “Pharyngeal Malignancy”.ti, ab OR “Pharyngeal Malignancies”.ti, ab OR “Salivary Gland Malignancy”.ti, ab OR “Salivary Gland Malignancies”.ti, ab OR “Sublingual Gland Malignancy”.ti, ab OR “Sublingual Gland Malignancies”.ti, ab OR “Submandibular Gland Malignancy”.ti, ab OR “Submandibular Gland Malignancies”.ti, ab OR “Thyroid Malignancy”.ti, ab OR “Thyroid Malignancies”.ti, ab OR “Tongue Malignancy”.ti, ab OR “Tongue Malignancies”.ti, ab OR “Tonsillar Malignancy”.ti, ab OR “Tonsillar Malignancies”.ti, ab OR “Tracheal Malignancy”.ti, ab OR “Tracheal Malignancies”.ti, ab OR “Thyroid Nodule”.ti, ab OR “Oral Leukoplakia”.ti, ab OR “Hairy Leukoplakia”.ti, ab OR exp *”Digestive System Tumor”/ OR “Gastrointestinal Neoplasms”.ti, ab OR “Gastrointestinal Neoplasm”.ti, ab OR “Stomach Neoplasms”.ti, ab OR “Stomach Neoplasm”.ti, ab OR “Gastric Neoplasms”.ti, ab OR “Gastric Neoplasm”.ti, ab OR “Esophageal Neoplasms”.ti, ab OR “Esophageal Neoplasm”.ti, ab OR “OEsophageal Neoplasms”.ti, ab OR “OEsophageal Neoplasm”.ti, ab OR “Esophagus Neoplasms”.ti, ab OR “Esophagus Neoplasm”.ti, ab OR “OEsophagus Neoplasms”.ti, ab OR “Esophagus Neoplasm”.ti, ab OR “Esophageal Squamus Cell Neoplasms”.ti, ab OR “Esophageal Squamus Cell Neoplasm”.ti, ab OR “OEsophageal Squamus Cell Neoplasms”.ti, ab OR “OEsophageal Squamus Cell Neoplasm”.ti, ab OR “Esophagus Squamus Cell Neoplasms”.ti, ab OR “Esophagus Squamus Cell Neoplasm”.ti, ab OR “OEsophagus Squamus Cell Neoplasms”.ti, ab OR “Esophagus Squamus Cell Neoplasm”.ti, ab OR “Intestinal Neoplasms”.ti, ab OR “Intestinal Neoplasm”.ti, ab OR “Cecal Neoplasms”.ti, ab OR “Cecal Neoplasm”.ti, ab OR “Appenciceal Neoplasms”.ti, ab OR “Appendiceal Neoplasm”.ti, ab OR “Colorectal Neoplasms”.ti, ab OR “Colorectal Neoplasm”.ti, ab OR “Colonic Neoplasms”.ti, ab OR “Colonic Neoplasm”.ti, ab OR “Colon Neoplasms”.ti, ab OR “Colon Neoplasm”.ti, ab OR “Sigmoidal Neoplasms”.ti, ab OR “Sigmoidal Neoplasm”.ti, ab OR “Sigmoid Neoplasms”.ti, ab OR “Sigmoid Neoplasm”.ti, ab OR “Rectal Neoplasms”.ti, ab OR “Rectal Neoplasm”.ti, ab OR “Anorectal Neoplasms”.ti, ab OR “Anorectal Neoplasm”.ti, ab OR “Anus Neoplasms”.ti, ab OR “Anus Neoplasm”.ti, ab OR “Anal Neoplasms”.ti, ab OR “Anal Neoplasm”.ti, ab OR “Anal Gland Neoplasms”.ti, ab OR “Anal Gland Neoplasm”.ti, ab OR “Duodenal Neoplasms”.ti, ab OR “Duodenal Neoplasm”.ti, ab OR “Duodenum Neoplasms”.ti, ab OR “Duodenum Neoplasm”.ti, ab OR “Ileal Neoplasms”.ti, ab OR “Ileal Neoplasm”.ti, ab OR “Ileum Neoplasms”.ti, ab OR “Ileum Neoplasm”.ti, ab OR “Jejunal Neoplasms”.ti, ab OR “Jejunal Neoplasm”.ti, ab OR “Jejunum Neoplasms”.ti, ab OR “Jejunum Neoplasm”.ti, ab OR “Gastrointestinal Cancers”.ti, ab OR “Gastrointestinal Cancer”.ti, ab OR “Stomach Cancers”.ti, ab OR “Stomach Cancer”.ti, ab OR “Gastric Cancers”.ti, ab OR “Gastric Cancer”.ti, ab OR “Esophageal Cancers”.ti, ab OR “Esophageal Cancer”.ti, ab OR “OEsophageal Cancers”.ti, ab OR “OEsophageal Cancer”.ti, ab OR “Esophagus Cancers”.ti, ab OR “Esophagus Cancer”.ti, ab OR “OEsophagus Cancers”.ti, ab OR “Esophagus Cancer”.ti, ab OR “Esophageal Squamus Cell Cancers”.ti, ab OR “Esophageal Squamus Cell Cancer”.ti, ab OR “OEsophageal Squamus Cell Cancers”.ti, ab OR “OEsophageal Squamus Cell Cancer”.ti, ab OR “Esophagus Squamus Cell Cancers”.ti, ab OR “Esophagus Squamus Cell Cancer”.ti, ab OR “OEsophagus Squamus Cell Cancers”.ti, ab OR “Esophagus Squamus Cell Cancer”.ti, ab OR “Intestinal Cancers”.ti, ab OR “Intestinal Cancer”.ti, ab OR “Cecal Cancers”.ti, ab OR “Cecal Cancer”.ti, ab OR “Appenciceal Cancers”.ti, ab OR “Appendiceal Cancer”.ti, ab OR “Colorectal Cancers”.ti, ab OR “Colorectal Cancer”.ti, ab OR “Colonic Cancers”.ti, ab OR “Colonic Cancer”.ti, ab OR “Colon Cancers”.ti, ab OR “Colon Cancer”.ti, ab OR “Sigmoidal Cancers”.ti, ab OR “Sigmoidal Cancer”.ti, ab OR “Sigmoid Cancers”.ti, ab OR “Sigmoid Cancer”.ti, ab OR “Rectal Cancers”.ti, ab OR “Rectal Cancer”.ti, ab OR “Anorectal Cancers”.ti, ab OR “Anorectal Cancer”.ti, ab OR “Anus Cancers”.ti, ab OR “Anus Cancer”.ti, ab OR “Anal Cancers”.ti, ab OR “Anal Cancer”.ti, ab OR “Anal Gland Cancers”.ti, ab OR “Anal Gland Cancer”.ti, ab OR “Duodenal Cancers”.ti, ab OR “Duodenal Cancer”.ti, ab OR “Duodenum Cancers”.ti, ab OR “Duodenum Cancer”.ti, ab OR “Ileal Cancers”.ti, ab OR “Ileal Cancer”.ti, ab OR “Ileum Cancers”.ti, ab OR “Ileum Cancer”.ti, ab OR “Jejunal Cancers”.ti, ab OR “Jejunal Cancer”.ti, ab OR “Jejunum Cancers”.ti, ab OR “Jejunum Cancer”.ti, ab OR “Gastrointestinal Carcinomas”.ti, ab OR “Gastrointestinal Carcinoma”.ti, ab OR “Stomach Carcinomas”.ti, ab OR “Stomach Carcinoma”.ti, ab OR “Gastric Carcinomas”.ti, ab OR “Gastric Carcinoma”.ti, ab OR “Esophageal Carcinomas”.ti, ab OR “Esophageal Carcinoma”.ti, ab OR “OEsophageal Carcinomas”.ti, ab OR “OEsophageal Carcinoma”.ti, ab OR “Esophagus Carcinomas”.ti, ab OR “Esophagus Carcinoma”.ti, ab OR “OEsophagus Carcinomas”.ti, ab OR “Esophagus Carcinoma”.ti, ab OR “Esophageal Squamus Cell Carcinomas”.ti, ab OR “Esophageal Squamus Cell Carcinoma”.ti, ab OR “OEsophageal Squamus Cell Carcinomas”.ti, ab OR “OEsophageal Squamus Cell Carcinoma”.ti, ab OR “Esophagus Squamus Cell Carcinomas”.ti, ab OR “Esophagus Squamus Cell Carcinoma”.ti, ab OR “OEsophagus Squamus Cell Carcinomas”.ti, ab OR “Esophagus Squamus Cell Carcinoma”.ti, ab OR “Intestinal Carcinomas”.ti, ab OR “Intestinal Carcinoma”.ti, ab OR “Cecal Carcinomas”.ti, ab OR “Cecal Carcinoma”.ti, ab OR “Appenciceal Carcinomas”.ti, ab OR “Appendiceal Carcinoma”.ti, ab OR “Colorectal Carcinomas”.ti, ab OR “Colorectal Carcinoma”.ti, ab OR “Colonic Carcinomas”.ti, ab OR “Colonic Carcinoma”.ti, ab OR “Colon Carcinomas”.ti, ab OR “Colon Carcinoma”.ti, ab OR “Sigmoidal Carcinomas”.ti, ab OR “Sigmoidal Carcinoma”.ti, ab OR “Sigmoid Carcinomas”.ti, ab OR “Sigmoid Carcinoma”.ti, ab OR “Rectal Carcinomas”.ti, ab OR “Rectal Carcinoma”.ti, ab OR “Anorectal Carcinomas”.ti, ab OR “Anorectal Carcinoma”.ti, ab OR “Anus Carcinomas”.ti, ab OR “Anus Carcinoma”.ti, ab OR “Anal Carcinomas”.ti, ab OR “Anal Carcinoma”.ti, ab OR “Anal Gland Carcinomas”.ti, ab OR “Anal Gland Carcinoma”.ti, ab OR “Duodenal Carcinomas”.ti, ab OR “Duodenal Carcinoma”.ti, ab OR “Duodenum Carcinomas”.ti, ab OR “Duodenum Carcinoma”.ti, ab OR “Ileal Carcinomas”.ti, ab OR “Ileal Carcinoma”.ti, ab OR “Ileum Carcinomas”.ti, ab OR “Ileum Carcinoma”.ti, ab OR “Jejunal Carcinomas”.ti, ab OR “Jejunal Carcinoma”.ti, ab OR “Jejunum Carcinomas”.ti, ab OR “Jejunum Carcinoma”.ti, ab OR “Gastrointestinal Adenocarcinomas”.ti, ab OR “Gastrointestinal Adenocarcinoma”.ti, ab OR “Stomach Adenocarcinomas”.ti, ab OR “Stomach Adenocarcinoma”.ti, ab OR “Gastric Adenocarcinomas”.ti, ab OR “Gastric Adenocarcinoma”.ti, ab OR “Esophageal Adenocarcinomas”.ti, ab OR “Esophageal Adenocarcinoma”.ti, ab OR “OEsophageal Adenocarcinomas”.ti, ab OR “OEsophageal Adenocarcinoma”.ti, ab OR “Esophagus Adenocarcinomas”.ti, ab OR “Esophagus Adenocarcinoma”.ti, ab OR “OEsophagus Adenocarcinomas”.ti, ab OR “Esophagus Adenocarcinoma”.ti, ab OR “Esophageal Squamus Cell Adenocarcinomas”.ti, ab OR “Esophageal Squamus Cell Adenocarcinoma”.ti, ab OR “OEsophageal Squamus Cell Adenocarcinomas”.ti, ab OR “OEsophageal Squamus Cell Adenocarcinoma”.ti, ab OR “Esophagus Squamus Cell Adenocarcinomas”.ti, ab OR “Esophagus Squamus Cell Adenocarcinoma”.ti, ab OR “OEsophagus Squamus Cell Adenocarcinomas”.ti, ab OR “Esophagus Squamus Cell Adenocarcinoma”.ti, ab OR “Intestinal Adenocarcinomas”.ti, ab OR “Intestinal Adenocarcinoma”.ti, ab OR “Cecal Adenocarcinomas”.ti, ab OR “Cecal Adenocarcinoma”.ti, ab OR “Appenciceal Adenocarcinomas”.ti, ab OR “Appendiceal Adenocarcinoma”.ti, ab OR “Colorectal Adenocarcinomas”.ti, ab OR “Colorectal Adenocarcinoma”.ti, ab OR “Colonic Adenocarcinomas”.ti, ab OR “Colonic Adenocarcinoma”.ti, ab OR “Colon Adenocarcinomas”.ti, ab OR “Colon Adenocarcinoma”.ti, ab OR “Sigmoidal Adenocarcinomas”.ti, ab OR “Sigmoidal Adenocarcinoma”.ti, ab OR “Sigmoid Adenocarcinomas”.ti, ab OR “Sigmoid Adenocarcinoma”.ti, ab OR “Rectal Adenocarcinomas”.ti, ab OR “Rectal Adenocarcinoma”.ti, ab OR “Anorectal Adenocarcinomas”.ti, ab OR “Anorectal Adenocarcinoma”.ti, ab OR “Anus Adenocarcinomas”.ti, ab OR “Anus Adenocarcinoma”.ti, ab OR “Anal Adenocarcinomas”.ti, ab OR “Anal Adenocarcinoma”.ti, ab OR “Anal Gland Adenocarcinomas”.ti, ab OR “Anal Gland Adenocarcinoma”.ti, ab OR “Duodenal Adenocarcinomas”.ti, ab OR “Duodenal Adenocarcinoma”.ti, ab OR “Duodenum Adenocarcinomas”.ti, ab OR “Duodenum Adenocarcinoma”.ti, ab OR “Ileal Adenocarcinomas”.ti, ab OR “Ileal Adenocarcinoma”.ti, ab OR “Ileum Adenocarcinomas”.ti, ab OR “Ileum Adenocarcinoma”.ti, ab OR “Jejunal Adenocarcinomas”.ti, ab OR “Jejunal Adenocarcinoma”.ti, ab OR “Jejunum Adenocarcinomas”.ti, ab OR “Jejunum Adenocarcinoma”.ti, ab OR “Gastrointestinal Tumors”.ti, ab OR “Gastrointestinal Tumor”.ti, ab OR “Stomach Tumors”.ti, ab OR “Stomach Tumor”.ti, ab OR “Gastric Tumors”.ti, ab OR “Gastric Tumor”.ti, ab OR “Esophageal Tumors”.ti, ab OR “Esophageal Tumor”.ti, ab OR “OEsophageal Tumors”.ti, ab OR “OEsophageal Tumor”.ti, ab OR “Esophagus Tumors”.ti, ab OR “Esophagus Tumor”.ti, ab OR “OEsophagus Tumors”.ti, ab OR “Esophagus Tumor”.ti, ab OR “Esophageal Squamus Cell Tumors”.ti, ab OR “Esophageal Squamus Cell Tumor”.ti, ab OR “OEsophageal Squamus Cell Tumors”.ti, ab OR “OEsophageal Squamus Cell Tumor”.ti, ab OR “Esophagus Squamus Cell Tumors”.ti, ab OR “Esophagus Squamus Cell Tumor”.ti, ab OR “OEsophagus Squamus Cell Tumors”.ti, ab OR “Esophagus Squamus Cell Tumor”.ti, ab OR “Intestinal Tumors”.ti, ab OR “Intestinal Tumor”.ti, ab OR “Cecal Tumors”.ti, ab OR “Cecal Tumor”.ti, ab OR “Appenciceal Tumors”.ti, ab OR “Appendiceal Tumor”.ti, ab OR “Colorectal Tumors”.ti, ab OR “Colorectal Tumor”.ti, ab OR “Colonic Tumors”.ti, ab OR “Colonic Tumor”.ti, ab OR “Colon Tumors”.ti, ab OR “Colon Tumor”.ti, ab OR “Sigmoidal Tumors”.ti, ab OR “Sigmoidal Tumor”.ti, ab OR “Sigmoid Tumors”.ti, ab OR “Sigmoid Tumor”.ti, ab OR “Rectal Tumors”.ti, ab OR “Rectal Tumor”.ti, ab OR “Anorectal Tumors”.ti, ab OR “Anorectal Tumor”.ti, ab OR “Anus Tumors”.ti, ab OR “Anus Tumor”.ti, ab OR “Anal Tumors”.ti, ab OR “Anal Tumor”.ti, ab OR “Anal Gland Tumors”.ti, ab OR “Anal Gland Tumor”.ti, ab OR “Duodenal Tumors”.ti, ab OR “Duodenal Tumor”.ti, ab OR “Duodenum Tumors”.ti, ab OR “Duodenum Tumor”.ti, ab OR “Ileal Tumors”.ti, ab OR “Ileal Tumor”.ti, ab OR “Ileum Tumors”.ti, ab OR “Ileum Tumor”.ti, ab OR “Jejunal Tumors”.ti, ab OR “Jejunal Tumor”.ti, ab OR “Jejunum Tumors”.ti, ab OR “Jejunum Tumor”.ti, ab OR “Gastrointestinal Tumours”.ti, ab OR “Gastrointestinal Tumour”.ti, ab OR “Stomach Tumours”.ti, ab OR “Stomach Tumour”.ti, ab OR “Gastric Tumours”.ti, ab OR “Gastric Tumour”.ti, ab OR “Esophageal Tumours”.ti, ab OR “Esophageal Tumour”.ti, ab OR “OEsophageal Tumours”.ti, ab OR “OEsophageal Tumour”.ti, ab OR “Esophagus Tumours”.ti, ab OR “Esophagus Tumour”.ti, ab OR “OEsophagus Tumours”.ti, ab OR “Esophagus Tumour”.ti, ab OR “Esophageal Squamus Cell Tumours”.ti, ab OR “Esophageal Squamus Cell Tumour”.ti, ab OR “OEsophageal Squamus Cell Tumours”.ti, ab OR “OEsophageal Squamus Cell Tumour”.ti, ab OR “Esophagus Squamus Cell Tumours”.ti, ab OR “Esophagus Squamus Cell Tumour”.ti, ab OR “OEsophagus Squamus Cell Tumours”.ti, ab OR “Esophagus Squamus Cell Tumour”.ti, ab OR “Intestinal Tumours”.ti, ab OR “Intestinal Tumour”.ti, ab OR “Cecal Tumours”.ti, ab OR “Cecal Tumour”.ti, ab OR “Appenciceal Tumours”.ti, ab OR “Appendiceal Tumour”.ti, ab OR “Colorectal Tumours”.ti, ab OR “Colorectal Tumour”.ti, ab OR “Colonic Tumours”.ti, ab OR “Colonic Tumour”.ti, ab OR “Colon Tumours”.ti, ab OR “Colon Tumour”.ti, ab OR “Sigmoidal Tumours”.ti, ab OR “Sigmoidal Tumour”.ti, ab OR “Sigmoid Tumours”.ti, ab OR “Sigmoid Tumour”.ti, ab OR “Rectal Tumours”.ti, ab OR “Rectal Tumour”.ti, ab OR “Anorectal Tumours”.ti, ab OR “Anorectal Tumour”.ti, ab OR “Anus Tumours”.ti, ab OR “Anus Tumour”.ti, ab OR “Anal Tumours”.ti, ab OR “Anal Tumour”.ti, ab OR “Anal Gland Tumours”.ti, ab OR “Anal Gland Tumour”.ti, ab OR “Duodenal Tumours”.ti, ab OR “Duodenal Tumour”.ti, ab OR “Duodenum Tumours”.ti, ab OR “Duodenum Tumour”.ti, ab OR “Ileal Tumours”.ti, ab OR “Ileal Tumour”.ti, ab OR “Ileum Tumours”.ti, ab OR “Ileum Tumour”.ti, ab OR “Jejunal Tumours”.ti, ab OR “Jejunal Tumour”.ti, ab OR “Jejunum Tumours”.ti, ab OR “Jejunum Tumour”.ti, ab OR “Gastrointestinal Malignancies”.ti, ab OR “Gastrointestinal Malignancy”.ti, ab OR “Stomach Malignancies”.ti, ab OR “Stomach Malignancy”.ti, ab OR “Gastric Malignancies”.ti, ab OR “Gastric Malignancy”.ti, ab OR “Esophageal Malignancies”.ti, ab OR “Esophageal Malignancy”.ti, ab OR “OEsophageal Malignancies”.ti, ab OR “OEsophageal Malignancy”.ti, ab OR “Esophagus Malignancies”.ti, ab OR “Esophagus Malignancy”.ti, ab OR “OEsophagus Malignancies”.ti, ab OR “Esophagus Malignancy”.ti, ab OR “Esophageal Squamus Cell Malignancies”.ti, ab OR “Esophageal Squamus Cell Malignancy”.ti, ab OR “OEsophageal Squamus Cell Malignancies”.ti, ab OR “OEsophageal Squamus Cell Malignancy”.ti, ab OR “Esophagus Squamus Cell Malignancies”.ti, ab OR “Esophagus Squamus Cell Malignancy”.ti, ab OR “OEsophagus Squamus Cell Malignancies”.ti, ab OR “Esophagus Squamus Cell Malignancy”.ti, ab OR “Intestinal Malignancies”.ti, ab OR “Intestinal Malignancy”.ti, ab OR “Cecal Malignancies”.ti, ab OR “Cecal Malignancy”.ti, ab OR “Appenciceal Malignancies”.ti, ab OR “Appendiceal Malignancy”.ti, ab OR “Colorectal Malignancies”.ti, ab OR “Colorectal Malignancy”.ti, ab OR “Colonic Malignancies”.ti, ab OR “Colonic Malignancy”.ti, ab OR “Colon Malignancies”.ti, ab OR “Colon Malignancy”.ti, ab OR “Sigmoidal Malignancies”.ti, ab OR “Sigmoidal Malignancy”.ti, ab OR “Sigmoid Malignancies”.ti, ab OR “Sigmoid Malignancy”.ti, ab OR “Rectal Malignancies”.ti, ab OR “Rectal Malignancy”.ti, ab OR “Anorectal Malignancies”.ti, ab OR “Anorectal Malignancy”.ti, ab OR “Anus Malignancies”.ti, ab OR “Anus Malignancy”.ti, ab OR “Anal Malignancies”.ti, ab OR “Anal Malignancy”.ti, ab OR “Anal Gland Malignancies”.ti, ab OR “Anal Gland Malignancy”.ti, ab OR “Duodenal Malignancies”.ti, ab OR “Duodenal Malignancy”.ti, ab OR “Duodenum Malignancies”.ti, ab OR “Duodenum Malignancy”.ti, ab OR “Ileal Malignancies”.ti, ab OR “Ileal Malignancy”.ti, ab OR “Ileum Malignancies”.ti, ab OR “Ileum Malignancy”.ti, ab OR “Jejunal Malignancies”.ti, ab OR “Jejunal Malignancy”.ti, ab OR “Jejunum Malignancies”.ti, ab OR “Jejunum Malignancy”.ti, ab OR “Adenomatous Polyposis Coli”.ti, ab OR “Gardner Syndrome”.ti, ab) AND (“resection”.mp OR “resect*”.mp OR “surgery”.mp OR exp “surgery”/ OR “surgical”.mp OR exp “gastrectomy”/ OR “gastrectomy”.mp OR “gastrectom*”.mp OR exp *”Preoperative Period”/ OR exp *”Preoperative Care”/ OR “Preoperative Period”.ti, ab OR “Preoperative Care”.ti, ab OR exp *”Perioperative Period”/ OR exp *”Perioperative Care”/ OR “Perioperative Period”.ti, ab OR “Perioperative Care”.ti, ab OR exp *”Postoperative Period”/ OR exp *”Postoperative Care”/ OR “Postoperative Period”.ti, ab OR “Postoperative Care”.ti, ab OR “Preoperative”.ti, ab OR “Pre operative”.ti, ab OR “Perioperative”.ti, ab OR “Peri operative”.ti, ab OR “Postoperative”.ti, ab OR “Post operative”.ti, ab) AND (“immunonutrition “/ OR “immunonutrition”.mp OR “immunonutr*”.mp OR “immuno nutrition”.mp OR “immuno nutr*”.mp OR ((exp “Immunomodulation”/ OR “Immunomodulation”.mp OR “Immunomodulat*”.mp OR exp “Immunotherapy”/ OR “immunother*”.mp) AND (“Nutrition Therapy”.ti, ab OR exp *”Diet Therapy”/ OR “Diet Therapy”.ti, ab OR “Caloric Restriction”.ti, ab OR “Carbohydrate Loading diet”.ti, ab OR “Carbohydrate-Restricted diet”.ti, ab OR “High-Protein Low-Carbohydrate diet”.ti, ab OR “Ketogenic diet”.ti, ab OR “Diabetic diet”.ti, ab OR “Fat-Restricted diet”.ti, ab OR “Gluten-Free diet”.ti, ab OR “High-Protein diet”.ti, ab OR “High-Protein Low-Carbohydrate diet”.ti, ab OR “Mediterranean diet”.ti, ab OR “Paleolithic diet”.ti, ab OR “Protein-Restricted diet”.ti, ab OR “Reducing diet”.ti, ab OR “Sodium-Restricted diet”.ti, ab OR “Vegetarian diet”.ti, ab OR “Macrobiotic diet”.ti, ab OR “Vegan diet”.ti, ab OR “Carbohydrate Loading diets”.ti, ab OR “Carbohydrate-Restricted diets”.ti, ab OR “High-Protein Low-Carbohydrate diets”.ti, ab OR “Ketogenic diets”.ti, ab OR “Diabetic diets”.ti, ab OR “Fat-Restricted diets”.ti, ab OR “Gluten-Free diets”.ti, ab OR “High-Protein diets”.ti, ab OR “High-Protein Low-Carbohydrate diets”.ti, ab OR “Mediterranean diets”.ti, ab OR “Paleolithic diets”.ti, ab OR “Protein-Restricted diets”.ti, ab OR “Reducing diets”.ti, ab OR “Sodium-Restricted diets”.ti, ab OR “Vegetarian diets”.ti, ab OR “Macrobiotic diets”.ti, ab OR “Vegan diets”.ti, ab OR “Nutritional Support”.ti, ab OR “Enteral Nutrition”.ti, ab OR “Parenteral Nutrition”.ti, ab OR “Enteral Feeding”.ti, ab OR “Parenteral Feeding”.ti, ab OR “nutritional supplementation”.ti, ab OR “artifical nutrition”.ti, ab))) AND (exp “clinical trial”/ OR “clinical trial”.ti, ab OR exp “clinical trials as topic”/ OR “clinical trials”.ti, ab OR exp “control group”/ OR “control group”.ti, ab OR “control groups”.ti, ab OR exp “controlled clinical trial”/ OR exp “controlled clinical trials as topic”/ OR exp “crossover procedure”/ OR “cross over study”.ti, ab OR “cross over studies”.ti, ab OR exp “double-blind method”/ OR “double blind”.ti, ab OR exp “evaluation study”/ OR “follow up”/ OR “follow up study”.ti, ab OR “follow up studies”.ti, ab OR exp “placebo”/ OR placebo*.ti, ab OR placebos*.ti, ab OR exp “pragmatic clinical trial”/ OR exp “prospective study”/ OR “prospective study”.ti, ab OR “prospective studies”.ti, ab OR “RaCT”.ti, ab OR “RaCTs”.ti, ab OR exp “randomization”/ OR “randomised”.ti, ab OR exp “randomized controlled trial”/ OR exp “randomized controlled trials as topic”/ OR “randomized”.ti, ab OR random*.ti, ab OR “RCT”.ti, ab OR “RCTs”.ti, ab OR “single blind”.ti, ab OR exp “single blind procedure”/ OR ((single*.ti, ab OR double*.ti, ab OR triple*.ti, ab) ADJ3 (blind*.ti, ab OR mask*.ti, ab)) OR volunteer*.ti, ab OR “trial”.ti OR “trials”.ti))

**Web of Science Core Collection**

(TS=(“Head and Neck Neoplasms” OR “Head and Neck Neoplasms” OR “Head and Neck Neoplasm” OR “Ear Neoplasm” OR “Ear Neoplasms” OR “Eyelid Neoplasm” OR “Eyelid Neoplasms” OR “Facial Neoplasm” OR “Facial Neoplasms” OR “Gingival Neoplasm” OR “Gingival Neoplasms” OR “Hypopharyngeal Neoplasm” OR “Hypopharyngeal Neoplasms” OR “Laryngeal Neoplasm” OR “Laryngeal Neoplasms” OR “Lip Neoplasm” OR “Lip Neoplasms” OR “Maxillary Sinus Neoplasm” OR “Maxillary Sinus Neoplasms” OR “Mouth Neoplasm” OR “Mouth Neoplasms” OR “Nasopharyngeal Neoplasm” OR “Nasopharyngeal Neoplasms” OR “Nose Neoplasm” OR “Nose Neoplasms” OR “Oropharyngeal Neoplasm” OR “Oropharyngeal Neoplasms” OR “Otorhinolaryngologic Neoplasm” OR “Otorhinolaryngologic Neoplasms” OR “Palatal Neoplasm” OR “Palatal Neoplasms” OR “Papillary Thyroid Neoplasm “ OR “Papillary Thyroid Neoplasms “ OR “Paranasal Sinus Neoplasm” OR “Paranasal Sinus Neoplasms” OR “Parathyroid Neoplasm” OR “Parathyroid Neoplasms” OR “Parotid Neoplasm” OR “Parotid Neoplasms” OR “Pharyngeal Neoplasm” OR “Pharyngeal Neoplasms” OR “Salivary Gland Neoplasm” OR “Salivary Gland Neoplasms” OR “Sublingual Gland Neoplasm” OR “Sublingual Gland Neoplasms” OR “Submandibular Gland Neoplasm” OR “Submandibular Gland Neoplasms” OR “Thyroid Neoplasm” OR “Thyroid Neoplasms” OR “Tongue Neoplasm” OR “Tongue Neoplasms” OR “Tonsillar Neoplasm” OR “Tonsillar Neoplasms” OR “Tracheal Neoplasm” OR “Tracheal Neoplasms” OR “Head and Neck Cancers” OR “Head and Neck Cancer” OR “Ear Cancer” OR “Ear Cancers” OR “Eyelid Cancer” OR “Eyelid Cancers” OR “Facial Cancer” OR “Facial Cancers” OR “Gingival Cancer” OR “Gingival Cancers” OR “Hypopharyngeal Cancer” OR “Hypopharyngeal Cancers” OR “Laryngeal Cancer” OR “Laryngeal Cancers” OR “Lip Cancer” OR “Lip Cancers” OR “Maxillary Sinus Cancer” OR “Maxillary Sinus Cancers” OR “Mouth Cancer” OR “Mouth Cancers” OR “Nasopharyngeal Cancer” OR “Nasopharyngeal Cancers” OR “Nose Cancer” OR “Nose Cancers” OR “Oropharyngeal Cancer” OR “Oropharyngeal Cancers” OR “Otorhinolaryngologic Cancer” OR “Otorhinolaryngologic Cancers” OR “Palatal Cancer” OR “Palatal Cancers” OR “Papillary Thyroid Cancer “ OR “Papillary Thyroid Cancers “ OR “Paranasal Sinus Cancer” OR “Paranasal Sinus Cancers” OR “Parathyroid Cancer” OR “Parathyroid Cancers” OR “Parotid Cancer” OR “Parotid Cancers” OR “Pharyngeal Cancer” OR “Pharyngeal Cancers” OR “Salivary Gland Cancer” OR “Salivary Gland Cancers” OR “Sublingual Gland Cancer” OR “Sublingual Gland Cancers” OR “Submandibular Gland Cancer” OR “Submandibular Gland Cancers” OR “Thyroid Cancer” OR “Thyroid Cancers” OR “Tongue Cancer” OR “Tongue Cancers” OR “Tonsillar Cancer” OR “Tonsillar Cancers” OR “Tracheal Cancer” OR “Tracheal Cancers” OR “Head and Neck Carcinomas” OR “Head and Neck Carcinoma” OR “Ear Carcinoma” OR “Ear Carcinomas” OR “Eyelid Carcinoma” OR “Eyelid Carcinomas” OR “Facial Carcinoma” OR “Facial Carcinomas” OR “Gingival Carcinoma” OR “Gingival Carcinomas” OR “Hypopharyngeal Carcinoma” OR “Hypopharyngeal Carcinomas” OR “Laryngeal Carcinoma” OR “Laryngeal Carcinomas” OR “Lip Carcinoma” OR “Lip Carcinomas” OR “Maxillary Sinus Carcinoma” OR “Maxillary Sinus Carcinomas” OR “Mouth Carcinoma” OR “Mouth Carcinomas” OR “Nasopharyngeal Carcinoma” OR “Nasopharyngeal Carcinomas” OR “Nose Carcinoma” OR “Nose Carcinomas” OR “Oropharyngeal Carcinoma” OR “Oropharyngeal Carcinomas” OR “Otorhinolaryngologic Carcinoma” OR “Otorhinolaryngologic Carcinomas” OR “Palatal Carcinoma” OR “Palatal Carcinomas” OR “Papillary Thyroid Carcinoma “ OR “Papillary Thyroid Carcinomas “ OR “Paranasal Sinus Carcinoma” OR “Paranasal Sinus Carcinomas” OR “Parathyroid Carcinoma” OR “Parathyroid Carcinomas” OR “Parotid Carcinoma” OR “Parotid Carcinomas” OR “Pharyngeal Carcinoma” OR “Pharyngeal Carcinomas” OR “Salivary Gland Carcinoma” OR “Salivary Gland Carcinomas” OR “Sublingual Gland Carcinoma” OR “Sublingual Gland Carcinomas” OR “Submandibular Gland Carcinoma” OR “Submandibular Gland Carcinomas” OR “Thyroid Carcinoma” OR “Thyroid Carcinomas” OR “Tongue Carcinoma” OR “Tongue Carcinomas” OR “Tonsillar Carcinoma” OR “Tonsillar Carcinomas” OR “Tracheal Carcinoma” OR “Tracheal Carcinomas” OR “Head and Neck Adenocarcinomas” OR “Head and Neck Adenocarcinoma” OR “Ear Adenocarcinoma” OR “Ear Adenocarcinomas” OR “Eyelid Adenocarcinoma” OR “Eyelid Adenocarcinomas” OR “Facial Adenocarcinoma” OR “Facial Adenocarcinomas” OR “Gingival Adenocarcinoma” OR “Gingival Adenocarcinomas” OR “Hypopharyngeal Adenocarcinoma” OR “Hypopharyngeal Adenocarcinomas” OR “Laryngeal Adenocarcinoma” OR “Laryngeal Adenocarcinomas” OR “Lip Adenocarcinoma” OR “Lip Adenocarcinomas” OR “Maxillary Sinus Adenocarcinoma” OR “Maxillary Sinus Adenocarcinomas” OR “Mouth Adenocarcinoma” OR “Mouth Adenocarcinomas” OR “Nasopharyngeal Adenocarcinoma” OR “Nasopharyngeal Adenocarcinomas” OR “Nose Adenocarcinoma” OR “Nose Adenocarcinomas” OR “Oropharyngeal Adenocarcinoma” OR “Oropharyngeal Adenocarcinomas” OR “Otorhinolaryngologic Adenocarcinoma” OR “Otorhinolaryngologic Adenocarcinomas” OR “Palatal Adenocarcinoma” OR “Palatal Adenocarcinomas” OR “Papillary Thyroid Adenocarcinoma “ OR “Papillary Thyroid Adenocarcinomas “ OR “Paranasal Sinus Adenocarcinoma” OR “Paranasal Sinus Adenocarcinomas” OR “Parathyroid Adenocarcinoma” OR “Parathyroid Adenocarcinomas” OR “Parotid Adenocarcinoma” OR “Parotid Adenocarcinomas” OR “Pharyngeal Adenocarcinoma” OR “Pharyngeal Adenocarcinomas” OR “Salivary Gland Adenocarcinoma” OR “Salivary Gland Adenocarcinomas” OR “Sublingual Gland Adenocarcinoma” OR “Sublingual Gland Adenocarcinomas” OR “Submandibular Gland Adenocarcinoma” OR “Submandibular Gland Adenocarcinomas” OR “Thyroid Adenocarcinoma” OR “Thyroid Adenocarcinomas” OR “Tongue Adenocarcinoma” OR “Tongue Adenocarcinomas” OR “Tonsillar Adenocarcinoma” OR “Tonsillar Adenocarcinomas” OR “Tracheal Adenocarcinoma” OR “Tracheal Adenocarcinomas” OR “Head and Neck Tumors” OR “Head and Neck Tumor” OR “Ear Tumor” OR “Ear Tumors” OR “Eyelid Tumor” OR “Eyelid Tumors” OR “Facial Tumor” OR “Facial Tumors” OR “Gingival Tumor” OR “Gingival Tumors” OR “Hypopharyngeal Tumor” OR “Hypopharyngeal Tumors” OR “Laryngeal Tumor” OR “Laryngeal Tumors” OR “Lip Tumor” OR “Lip Tumors” OR “Maxillary Sinus Tumor” OR “Maxillary Sinus Tumors” OR “Mouth Tumor” OR “Mouth Tumors” OR “Nasopharyngeal Tumor” OR “Nasopharyngeal Tumors” OR “Nose Tumor” OR “Nose Tumors” OR “Oropharyngeal Tumor” OR “Oropharyngeal Tumors” OR “Otorhinolaryngologic Tumor” OR “Otorhinolaryngologic Tumors” OR “Palatal Tumor” OR “Palatal Tumors” OR “Papillary Thyroid Tumor “ OR “Papillary Thyroid Tumors “ OR “Paranasal Sinus Tumor” OR “Paranasal Sinus Tumors” OR “Parathyroid Tumor” OR “Parathyroid Tumors” OR “Parotid Tumor” OR “Parotid Tumors” OR “Pharyngeal Tumor” OR “Pharyngeal Tumors” OR “Salivary Gland Tumor” OR “Salivary Gland Tumors” OR “Sublingual Gland Tumor” OR “Sublingual Gland Tumors” OR “Submandibular Gland Tumor” OR “Submandibular Gland Tumors” OR “Thyroid Tumor” OR “Thyroid Tumors” OR “Tongue Tumor” OR “Tongue Tumors” OR “Tonsillar Tumor” OR “Tonsillar Tumors” OR “Tracheal Tumor” OR “Tracheal Tumors” OR “Head and Neck Tumours” OR “Head and Neck Tumour” OR “Ear Tumour” OR “Ear Tumours” OR “Eyelid Tumour” OR “Eyelid Tumours” OR “Facial Tumour” OR “Facial Tumours” OR “Gingival Tumour” OR “Gingival Tumours” OR “Hypopharyngeal Tumour” OR “Hypopharyngeal Tumours” OR “Laryngeal Tumour” OR “Laryngeal Tumours” OR “Lip Tumour” OR “Lip Tumours” OR “Maxillary Sinus Tumour” OR “Maxillary Sinus Tumours” OR “Mouth Tumour” OR “Mouth Tumours” OR “Nasopharyngeal Tumour” OR “Nasopharyngeal Tumours” OR “Nose Tumour” OR “Nose Tumours” OR “Oropharyngeal Tumour” OR “Oropharyngeal Tumours” OR “Otorhinolaryngologic Tumour” OR “Otorhinolaryngologic Tumours” OR “Palatal Tumour” OR “Palatal Tumours” OR “Papillary Thyroid Tumour “ OR “Papillary Thyroid Tumours “ OR “Paranasal Sinus Tumour” OR “Paranasal Sinus Tumours” OR “Parathyroid Tumour” OR “Parathyroid Tumours” OR “Parotid Tumour” OR “Parotid Tumours” OR “Pharyngeal Tumour” OR “Pharyngeal Tumours” OR “Salivary Gland Tumour” OR “Salivary Gland Tumours” OR “Sublingual Gland Tumour” OR “Sublingual Gland Tumours” OR “Submandibular Gland Tumour” OR “Submandibular Gland Tumours” OR “Thyroid Tumour” OR “Thyroid Tumours” OR “Tongue Tumour” OR “Tongue Tumours” OR “Tonsillar Tumour” OR “Tonsillar Tumours” OR “Tracheal Tumour” OR “Tracheal Tumours” OR “Head and Neck Malignancies” OR “Head and Neck Malignancy” OR “Ear Malignancy” OR “Ear Malignancies” OR “Eyelid Malignancy” OR “Eyelid Malignancies” OR “Facial Malignancy” OR “Facial Malignancies” OR “Gingival Malignancy” OR “Gingival Malignancies” OR “Hypopharyngeal Malignancy” OR “Hypopharyngeal Malignancies” OR “Laryngeal Malignancy” OR “Laryngeal Malignancies” OR “Lip Malignancy” OR “Lip Malignancies” OR “Maxillary Sinus Malignancy” OR “Maxillary Sinus Malignancies” OR “Mouth Malignancy” OR “Mouth Malignancies” OR “Nasopharyngeal Malignancy” OR “Nasopharyngeal Malignancies” OR “Nose Malignancy” OR “Nose Malignancies” OR “Oropharyngeal Malignancy” OR “Oropharyngeal Malignancies” OR “Otorhinolaryngologic Malignancy” OR “Otorhinolaryngologic Malignancies” OR “Palatal Malignancy” OR “Palatal Malignancies” OR “Papillary Thyroid Malignancy “ OR “Papillary Thyroid Malignancies “ OR “Paranasal Sinus Malignancy” OR “Paranasal Sinus Malignancies” OR “Parathyroid Malignancy” OR “Parathyroid Malignancies” OR “Parotid Malignancy” OR “Parotid Malignancies” OR “Pharyngeal Malignancy” OR “Pharyngeal Malignancies” OR “Salivary Gland Malignancy” OR “Salivary Gland Malignancies” OR “Sublingual Gland Malignancy” OR “Sublingual Gland Malignancies” OR “Submandibular Gland Malignancy” OR “Submandibular Gland Malignancies” OR “Thyroid Malignancy” OR “Thyroid Malignancies” OR “Tongue Malignancy” OR “Tongue Malignancies” OR “Tonsillar Malignancy” OR “Tonsillar Malignancies” OR “Tracheal Malignancy” OR “Tracheal Malignancies” OR “Thyroid Nodule” OR “Oral Leukoplakia” OR “Hairy Leukoplakia” OR “Gastrointestinal Neoplasms” OR “Gastrointestinal Neoplasms” OR “Gastrointestinal Neoplasm” OR “Stomach Neoplasms” OR “Stomach Neoplasm” OR “Gastric Neoplasms” OR “Gastric Neoplasm” OR “Esophageal Neoplasms” OR “Esophageal Neoplasm” OR “OEsophageal Neoplasms” OR “OEsophageal Neoplasm” OR “Esophagus Neoplasms” OR “Esophagus Neoplasm” OR “OEsophagus Neoplasms” OR “Esophagus Neoplasm” OR “Esophageal Squamus Cell Neoplasms” OR “Esophageal Squamus Cell Neoplasm” OR “OEsophageal Squamus Cell Neoplasms” OR “OEsophageal Squamus Cell Neoplasm” OR “Esophagus Squamus Cell Neoplasms” OR “Esophagus Squamus Cell Neoplasm” OR “OEsophagus Squamus Cell Neoplasms” OR “Esophagus Squamus Cell Neoplasm” OR “Intestinal Neoplasms” OR “Intestinal Neoplasm” OR “Cecal Neoplasms” OR “Cecal Neoplasm” OR “Appenciceal Neoplasms” OR “Appendiceal Neoplasm” OR “Colorectal Neoplasms” OR “Colorectal Neoplasm” OR “Colonic Neoplasms” OR “Colonic Neoplasm” OR “Colon Neoplasms” OR “Colon Neoplasm” OR “Sigmoidal Neoplasms” OR “Sigmoidal Neoplasm” OR “Sigmoid Neoplasms” OR “Sigmoid Neoplasm” OR “Rectal Neoplasms” OR “Rectal Neoplasm” OR “Anorectal Neoplasms” OR “Anorectal Neoplasm” OR “Anus Neoplasms” OR “Anus Neoplasm” OR “Anal Neoplasms” OR “Anal Neoplasm” OR “Anal Gland Neoplasms” OR “Anal Gland Neoplasm” OR “Duodenal Neoplasms” OR “Duodenal Neoplasm” OR “Duodenum Neoplasms” OR “Duodenum Neoplasm” OR “Ileal Neoplasms” OR “Ileal Neoplasm” OR “Ileum Neoplasms” OR “Ileum Neoplasm” OR “Jejunal Neoplasms” OR “Jejunal Neoplasm” OR “Jejunum Neoplasms” OR “Jejunum Neoplasm” OR “Gastrointestinal Cancers” OR “Gastrointestinal Cancer” OR “Stomach Cancers” OR “Stomach Cancer” OR “Gastric Cancers” OR “Gastric Cancer” OR “Esophageal Cancers” OR “Esophageal Cancer” OR “OEsophageal Cancers” OR “OEsophageal Cancer” OR “Esophagus Cancers” OR “Esophagus Cancer” OR “OEsophagus Cancers” OR “Esophagus Cancer” OR “Esophageal Squamus Cell Cancers” OR “Esophageal Squamus Cell Cancer” OR “OEsophageal Squamus Cell Cancers” OR “OEsophageal Squamus Cell Cancer” OR “Esophagus Squamus Cell Cancers” OR “Esophagus Squamus Cell Cancer” OR “OEsophagus Squamus Cell Cancers” OR “Esophagus Squamus Cell Cancer” OR “Intestinal Cancers” OR “Intestinal Cancer” OR “Cecal Cancers” OR “Cecal Cancer” OR “Appenciceal Cancers” OR “Appendiceal Cancer” OR “Colorectal Cancers” OR “Colorectal Cancer” OR “Colonic Cancers” OR “Colonic Cancer” OR “Colon Cancers” OR “Colon Cancer” OR “Sigmoidal Cancers” OR “Sigmoidal Cancer” OR “Sigmoid Cancers” OR “Sigmoid Cancer” OR “Rectal Cancers” OR “Rectal Cancer” OR “Anorectal Cancers” OR “Anorectal Cancer” OR “Anus Cancers” OR “Anus Cancer” OR “Anal Cancers” OR “Anal Cancer” OR “Anal Gland Cancers” OR “Anal Gland Cancer” OR “Duodenal Cancers” OR “Duodenal Cancer” OR “Duodenum Cancers” OR “Duodenum Cancer” OR “Ileal Cancers” OR “Ileal Cancer” OR “Ileum Cancers” OR “Ileum Cancer” OR “Jejunal Cancers” OR “Jejunal Cancer” OR “Jejunum Cancers” OR “Jejunum Cancer” OR “Gastrointestinal Carcinomas” OR “Gastrointestinal Carcinoma” OR “Stomach Carcinomas” OR “Stomach Carcinoma” OR “Gastric Carcinomas” OR “Gastric Carcinoma” OR “Esophageal Carcinomas” OR “Esophageal Carcinoma” OR “OEsophageal Carcinomas” OR “OEsophageal Carcinoma” OR “Esophagus Carcinomas” OR “Esophagus Carcinoma” OR “OEsophagus Carcinomas” OR “Esophagus Carcinoma” OR “Esophageal Squamus Cell Carcinomas” OR “Esophageal Squamus Cell Carcinoma” OR “OEsophageal Squamus Cell Carcinomas” OR “OEsophageal Squamus Cell Carcinoma” OR “Esophagus Squamus Cell Carcinomas” OR “Esophagus Squamus Cell Carcinoma” OR “OEsophagus Squamus Cell Carcinomas” OR “Esophagus Squamus Cell Carcinoma” OR “Intestinal Carcinomas” OR “Intestinal Carcinoma” OR “Cecal Carcinomas” OR “Cecal Carcinoma” OR “Appenciceal Carcinomas” OR “Appendiceal Carcinoma” OR “Colorectal Carcinomas” OR “Colorectal Carcinoma” OR “Colonic Carcinomas” OR “Colonic Carcinoma” OR “Colon Carcinomas” OR “Colon Carcinoma” OR “Sigmoidal Carcinomas” OR “Sigmoidal Carcinoma” OR “Sigmoid Carcinomas” OR “Sigmoid Carcinoma” OR “Rectal Carcinomas” OR “Rectal Carcinoma” OR “Anorectal Carcinomas” OR “Anorectal Carcinoma” OR “Anus Carcinomas” OR “Anus Carcinoma” OR “Anal Carcinomas” OR “Anal Carcinoma” OR “Anal Gland Carcinomas” OR “Anal Gland Carcinoma” OR “Duodenal Carcinomas” OR “Duodenal Carcinoma” OR “Duodenum Carcinomas” OR “Duodenum Carcinoma” OR “Ileal Carcinomas” OR “Ileal Carcinoma” OR “Ileum Carcinomas” OR “Ileum Carcinoma” OR “Jejunal Carcinomas” OR “Jejunal Carcinoma” OR “Jejunum Carcinomas” OR “Jejunum Carcinoma” OR “Gastrointestinal Adenocarcinomas” OR “Gastrointestinal Adenocarcinoma” OR “Stomach Adenocarcinomas” OR “Stomach Adenocarcinoma” OR “Gastric Adenocarcinomas” OR “Gastric Adenocarcinoma” OR “Esophageal Adenocarcinomas” OR “Esophageal Adenocarcinoma” OR “OEsophageal Adenocarcinomas” OR “OEsophageal Adenocarcinoma” OR “Esophagus Adenocarcinomas” OR “Esophagus Adenocarcinoma” OR “OEsophagus Adenocarcinomas” OR “Esophagus Adenocarcinoma” OR “Esophageal Squamus Cell Adenocarcinomas” OR “Esophageal Squamus Cell Adenocarcinoma” OR “OEsophageal Squamus Cell Adenocarcinomas” OR “OEsophageal Squamus Cell Adenocarcinoma” OR “Esophagus Squamus Cell Adenocarcinomas” OR “Esophagus Squamus Cell Adenocarcinoma” OR “OEsophagus Squamus Cell Adenocarcinomas” OR “Esophagus Squamus Cell Adenocarcinoma” OR “Intestinal Adenocarcinomas” OR “Intestinal Adenocarcinoma” OR “Cecal Adenocarcinomas” OR “Cecal Adenocarcinoma” OR “Appenciceal Adenocarcinomas” OR “Appendiceal Adenocarcinoma” OR “Colorectal Adenocarcinomas” OR “Colorectal Adenocarcinoma” OR “Colonic Adenocarcinomas” OR “Colonic Adenocarcinoma” OR “Colon Adenocarcinomas” OR “Colon Adenocarcinoma” OR “Sigmoidal Adenocarcinomas” OR “Sigmoidal Adenocarcinoma” OR “Sigmoid Adenocarcinomas” OR “Sigmoid Adenocarcinoma” OR “Rectal Adenocarcinomas” OR “Rectal Adenocarcinoma” OR “Anorectal Adenocarcinomas” OR “Anorectal Adenocarcinoma” OR “Anus Adenocarcinomas” OR “Anus Adenocarcinoma” OR “Anal Adenocarcinomas” OR “Anal Adenocarcinoma” OR “Anal Gland Adenocarcinomas” OR “Anal Gland Adenocarcinoma” OR “Duodenal Adenocarcinomas” OR “Duodenal Adenocarcinoma” OR “Duodenum Adenocarcinomas” OR “Duodenum Adenocarcinoma” OR “Ileal Adenocarcinomas” OR “Ileal Adenocarcinoma” OR “Ileum Adenocarcinomas” OR “Ileum Adenocarcinoma” OR “Jejunal Adenocarcinomas” OR “Jejunal Adenocarcinoma” OR “Jejunum Adenocarcinomas” OR “Jejunum Adenocarcinoma” OR “Gastrointestinal Tumors” OR “Gastrointestinal Tumor” OR “Stomach Tumors” OR “Stomach Tumor” OR “Gastric Tumors” OR “Gastric Tumor” OR “Esophageal Tumors” OR “Esophageal Tumor” OR “OEsophageal Tumors” OR “OEsophageal Tumor” OR “Esophagus Tumors” OR “Esophagus Tumor” OR “OEsophagus Tumors” OR “Esophagus Tumor” OR “Esophageal Squamus Cell Tumors” OR “Esophageal Squamus Cell Tumor” OR “OEsophageal Squamus Cell Tumors” OR “OEsophageal Squamus Cell Tumor” OR “Esophagus Squamus Cell Tumors” OR “Esophagus Squamus Cell Tumor” OR “OEsophagus Squamus Cell Tumors” OR “Esophagus Squamus Cell Tumor” OR “Intestinal Tumors” OR “Intestinal Tumor” OR “Cecal Tumors” OR “Cecal Tumor” OR “Appenciceal Tumors” OR “Appendiceal Tumor” OR “Colorectal Tumors” OR “Colorectal Tumor” OR “Colonic Tumors” OR “Colonic Tumor” OR “Colon Tumors” OR “Colon Tumor” OR “Sigmoidal Tumors” OR “Sigmoidal Tumor” OR “Sigmoid Tumors” OR “Sigmoid Tumor” OR “Rectal Tumors” OR “Rectal Tumor” OR “Anorectal Tumors” OR “Anorectal Tumor” OR “Anus Tumors” OR “Anus Tumor” OR “Anal Tumors” OR “Anal Tumor” OR “Anal Gland Tumors” OR “Anal Gland Tumor” OR “Duodenal Tumors” OR “Duodenal Tumor” OR “Duodenum Tumors” OR “Duodenum Tumor” OR “Ileal Tumors” OR “Ileal Tumor” OR “Ileum Tumors” OR “Ileum Tumor” OR “Jejunal Tumors” OR “Jejunal Tumor” OR “Jejunum Tumors” OR “Jejunum Tumor” OR “Gastrointestinal Tumours” OR “Gastrointestinal Tumour” OR “Stomach Tumours” OR “Stomach Tumour” OR “Gastric Tumours” OR “Gastric Tumour” OR “Esophageal Tumours” OR “Esophageal Tumour” OR “OEsophageal Tumours” OR “OEsophageal Tumour” OR “Esophagus Tumours” OR “Esophagus Tumour” OR “OEsophagus Tumours” OR “Esophagus Tumour” OR “Esophageal Squamus Cell Tumours” OR “Esophageal Squamus Cell Tumour” OR “OEsophageal Squamus Cell Tumours” OR “OEsophageal Squamus Cell Tumour” OR “Esophagus Squamus Cell Tumours” OR “Esophagus Squamus Cell Tumour” OR “OEsophagus Squamus Cell Tumours” OR “Esophagus Squamus Cell Tumour” OR “Intestinal Tumours” OR “Intestinal Tumour” OR “Cecal Tumours” OR “Cecal Tumour” OR “Appenciceal Tumours” OR “Appendiceal Tumour” OR “Colorectal Tumours” OR “Colorectal Tumour” OR “Colonic Tumours” OR “Colonic Tumour” OR “Colon Tumours” OR “Colon Tumour” OR “Sigmoidal Tumours” OR “Sigmoidal Tumour” OR “Sigmoid Tumours” OR “Sigmoid Tumour” OR “Rectal Tumours” OR “Rectal Tumour” OR “Anorectal Tumours” OR “Anorectal Tumour” OR “Anus Tumours” OR “Anus Tumour” OR “Anal Tumours” OR “Anal Tumour” OR “Anal Gland Tumours” OR “Anal Gland Tumour” OR “Duodenal Tumours” OR “Duodenal Tumour” OR “Duodenum Tumours” OR “Duodenum Tumour” OR “Ileal Tumours” OR “Ileal Tumour” OR “Ileum Tumours” OR “Ileum Tumour” OR “Jejunal Tumours” OR “Jejunal Tumour” OR “Jejunum Tumours” OR “Jejunum Tumour” OR “Gastrointestinal Malignancies” OR “Gastrointestinal Malignancy” OR “Stomach Malignancies” OR “Stomach Malignancy” OR “Gastric Malignancies” OR “Gastric Malignancy” OR “Esophageal Malignancies” OR “Esophageal Malignancy” OR “OEsophageal Malignancies” OR “OEsophageal Malignancy” OR “Esophagus Malignancies” OR “Esophagus Malignancy” OR “OEsophagus Malignancies” OR “Esophagus Malignancy” OR “Esophageal Squamus Cell Malignancies” OR “Esophageal Squamus Cell Malignancy” OR “OEsophageal Squamus Cell Malignancies” OR “OEsophageal Squamus Cell Malignancy” OR “Esophagus Squamus Cell Malignancies” OR “Esophagus Squamus Cell Malignancy” OR “OEsophagus Squamus Cell Malignancies” OR “Esophagus Squamus Cell Malignancy” OR “Intestinal Malignancies” OR “Intestinal Malignancy” OR “Cecal Malignancies” OR “Cecal Malignancy” OR “Appenciceal Malignancies” OR “Appendiceal Malignancy” OR “Colorectal Malignancies” OR “Colorectal Malignancy” OR “Colonic Malignancies” OR “Colonic Malignancy” OR “Colon Malignancies” OR “Colon Malignancy” OR “Sigmoidal Malignancies” OR “Sigmoidal Malignancy” OR “Sigmoid Malignancies” OR “Sigmoid Malignancy” OR “Rectal Malignancies” OR “Rectal Malignancy” OR “Anorectal Malignancies” OR “Anorectal Malignancy” OR “Anus Malignancies” OR “Anus Malignancy” OR “Anal Malignancies” OR “Anal Malignancy” OR “Anal Gland Malignancies” OR “Anal Gland Malignancy” OR “Duodenal Malignancies” OR “Duodenal Malignancy” OR “Duodenum Malignancies” OR “Duodenum Malignancy” OR “Ileal Malignancies” OR “Ileal Malignancy” OR “Ileum Malignancies” OR “Ileum Malignancy” OR “Jejunal Malignancies” OR “Jejunal Malignancy” OR “Jejunum Malignancies” OR “Jejunum Malignancy” OR “Adenomatous Polyposis Coli” OR “Gardner Syndrome”) AND TS=(“resection” OR “resect*” OR “surgery” OR “surgical” OR “gastrectomy” OR “gastrectomy” OR “gastrectom*” OR “Preoperative Period” OR “Preoperative Care” OR “Preoperative Period” OR “Preoperative Care” OR “Perioperative Period” OR “Perioperative Care” OR “Perioperative Period” OR “Perioperative Care” OR “Postoperative Period” OR “Postoperative Care” OR “Postoperative Period” OR “Postoperative Care” OR “Preoperative” OR “Pre operative” OR “Perioperative” OR “Peri operative” OR “Postoperative” OR “Post operative”) AND TS=(“immunonutrition “ OR “immunonutrition” OR “immunonutr*” OR “immuno nutrition” OR “immuno nutr*” OR ((“Immunomodulation” OR “Immunomodulation” OR “Immunomodulat*” OR “Immunotherapy” OR “immunother*”) AND (“Nutrition Therapy” OR “Nutrition Therapy” OR “Diet Therapy” OR “Diet Therapy” OR “Caloric Restriction” OR “Carbohydrate Loading diet” OR “Carbohydrate Restricted diet” OR “High Protein Low Carbohydrate diet” OR “Ketogenic diet” OR “Diabetic diet” OR “Fat Restricted diet” OR “Gluten Free diet” OR “High Protein diet” OR “High Protein Low Carbohydrate diet” OR “Mediterranean diet” OR “Paleolithic diet” OR “Protein Restricted diet” OR “Reducing diet” OR “Sodium Restricted diet” OR “Vegetarian diet” OR “Macrobiotic diet” OR “Vegan diet” OR “Carbohydrate Loading diets” OR “Carbohydrate Restricted diets” OR “High Protein Low Carbohydrate diets” OR “Ketogenic diets” OR “Diabetic diets” OR “Fat Restricted diets” OR “Gluten Free diets” OR “High Protein diets” OR “High Protein Low Carbohydrate diets” OR “Mediterranean diets” OR “Paleolithic diets” OR “Protein Restricted diets” OR “Reducing diets” OR “Sodium Restricted diets” OR “Vegetarian diets” OR “Macrobiotic diets” OR “Vegan diets” OR “Nutritional Support” OR “Enteral Nutrition” OR “Parenteral Nutrition” OR “Enteral Feeding” OR “Parenteral Feeding” OR “nutritional supplementation” OR “artifical nutrition” OR “Diet, Food, and Nutrition”))) AND (TS=(“clinical trial” OR “clinical trial” OR “clinical trials as topic” OR “clinical trials” OR “control groups” OR “control group” OR “control groups” OR “controlled clinical trial” OR “controlled clinical trials as topic” OR “cross over studies” OR “cross over study” OR “cross over studies” OR “double blind method” OR “double blind” OR “evaluation studies as topic” OR “follow up studies” OR “follow up study” OR “follow up studies” OR “placebos” OR placebo* OR placebos* OR “pragmatic clinical trial” OR “prospective studies” OR “prospective study” OR “prospective studies” OR “RaCT” OR “RaCTs” OR “random allocation” OR “randomised” OR “randomized controlled trial” OR “randomized controlled trials as topic” OR “randomized” OR random* OR “RCT” OR “RCTs” OR “Research Design” OR “Research design” OR “Research designs” OR “single blind” OR “single blind method” OR ((single* OR double* OR triple*) AND (blind* OR mask*)) OR volunteer*) OR ti=(“trial” OR “trials”)))
